# The risk of COVID-19 death is much greater and age dependent with type I IFN autoantibodies

**DOI:** 10.1073/pnas.2200413119

**Published:** 2022-05-16

**Authors:** Jérémy Manry, Paul Bastard, Adrian Gervais, Tom Le Voyer, Jérémie Rosain, Quentin Philippot, Eleftherios Michailidis, Hans-Heinrich Hoffmann, Shohei Eto, Marina Garcia-Prat, Lucy Bizien, Alba Parra-Martínez, Rui Yang, Liis Haljasmägi, Mélanie Migaud, Karita Särekannu, Julia Maslovskaja, Nicolas de Prost, Yacine Tandjaoui-Lambiotte, Charles-Edouard Luyt, Blanca Amador-Borrero, Alexandre Gaudet, Julien Poissy, Pascal Morel, Pascale Richard, Fabrice Cognasse, Jesús Troya, Sophie Trouillet-Assant, Alexandre Belot, Kahina Saker, Pierre Garçon, Jacques G. Rivière, Jean-Christophe Lagier, Stéphanie Gentile, Lindsey B. Rosen, Elana Shaw, Tomohiro Morio, Junko Tanaka, David Dalmau, Pierre-Louis Tharaux, Damien Sene, Alain Stepanian, Bruno Mégarbane, Vasiliki Triantafyllia, Arnaud Fekkar, James R. Heath, José Luis Franco, Juan-Manuel Anaya, Jordi Solé-Violán, Luisa Imberti, Andrea Biondi, Paolo Bonfanti, Riccardo Castagnoli, Ottavia M. Delmonte, Yu Zhang, Andrew L. Snow, Steven M. Holland, Catherine M. Biggs, Marcela Moncada-Vélez, Andrés Augusto Arias, Lazaro Lorenzo, Soraya Boucherit, Dany Anglicheau, Anna M. Planas, Filomeen Haerynck, Sotirija Duvlis, Tayfun Ozcelik, Sevgi Keles, Ahmed A. Bousfiha, Jalila El Bakkouri, Carolina Ramirez-Santana, Stéphane Paul, Qiang Pan-Hammarström, Lennart Hammarström, Annabelle Dupont, Alina Kurolap, Christine N. Metz, Alessandro Aiuti, Giorgio Casari, Vito Lampasona, Fabio Ciceri, Lucila A. Barreiros, Elena Dominguez-Garrido, Mateus Vidigal, Mayana Zatz, Diederik van de Beek, Sabina Sahanic, Ivan Tancevski, Yurii Stepanovskyy, Oksana Boyarchuk, Yoko Nukui, Miyuki Tsumura, Loreto Vidaur, Stuart G. Tangye, Sonia Burrel, Darragh Duffy, Lluis Quintana-Murci, Adam Klocperk, Nelli Y. Kann, Anna Shcherbina, Yu-Lung Lau, Daniel Leung, Matthieu Coulongeat, Julien Marlet, Rutger Koning, Luis Felipe Reyes, Angélique Chauvineau-Grenier, Fabienne Venet, Guillaume Monneret, Michel C. Nussenzweig, Romain Arrestier, Idris Boudhabhay, Hagit Baris-Feldman, David Hagin, Joost Wauters, Isabelle Meyts, Adam H. Dyer, Sean P. Kennelly, Nollaig M. Bourke, Rabih Halwani, Fatemeh Saheb Sharif-Askari, Karim Dorgham, Jérôme Sallette, Souad Mehlal Sedkaoui, Suzan AlKhater, Raúl Rigo-Bonnin, Francisco Morandeira, Lucie Roussel, Donald C. Vinh, Christian Erikstrup, Antonio Condino-Neto, Carolina Prando, Anastasiia Bondarenko, András N. Spaan, Laurent Gilardin, Jacques Fellay, Stanislas Lyonnet, Kaya Bilguvar, Richard P. Lifton, Shrikant Mane, HGID Lab, COVID Clinicians, COVID-STORM Clinicians, NIAID Immune Response to COVID Group, NH-COVAIR Study Group, Danish CHGE, Danish Blood Donor Study, St. James's Hospital, SARS CoV2 Interest Group, French COVID Cohort Study Group, Imagine COVID-Group, The Milieu Intérieur Consortium, CoV-Contact Cohort, Amsterdam UMC Covid-19 Biobank Investigators, COVID Human Genetic Effort, CP-COVID-19 Group, CONSTANCES cohort, 3C-Dijon Study, Cerba Health-Care, Etablissement Français du Sang Study group, Mark S. Anderson, Bertrand Boisson, Vivien Béziat, Shen-Ying Zhang, Evangelos Andreakos, Olivier Hermine, Aurora Pujol, Pärt Peterson, Trine H. Mogensen, Lee Rowen, James Mond, Stéphanie Debette, Xavier de Lamballerie, Charles Burdet, Lila Bouadma, Marie Zins, Pere Soler-Palacin, Roger Colobran, Guy Gorochov, Xavier Solanich, Sophie Susen, Javier Martinez-Picado, Didier Raoult, Marc Vasse, Peter K. Gregersen, Lorenzo Piemonti, Carlos Rodríguez-Gallego, Luigi D. Notarangelo, Helen C. Su, Kai Kisand, Satoshi Okada, Anne Puel, Emmanuelle Jouanguy, Charles M. Rice, Pierre Tiberghien, Qian Zhang, Jean-Laurent Casanova, Laurent Abel, Aurélie Cobat

**Affiliations:** ^a^Laboratory of Human Genetics of Infectious Diseases, Necker Branch, INSERM U1163, Necker Hospital for Sick Children, 75015 Paris, France;; ^b^Imagine Institute, University of Paris, 75015 Paris, France;; ^c^St. Giles Laboratory of Human Genetics of Infectious Diseases, Rockefeller Branch, Rockefeller University, New York, NY 10065;; ^d^Laboratory of Virology and Infectious Disease, Rockefeller University, New York, NY 10065;; ^e^Department of Pediatrics, Graduate School of Biomedical and Health Sciences, Hiroshima University, Hiroshima 734-8553, Japan;; ^f^Pediatric Infectious Diseases and Immunodeficiencies Unit, Hospital Universitari Vall d’Hebron, Vall d’Hebron Research Institute, Universitat Autònoma de Barcelona, 08035 Barcelona, Spain;; ^g^Institute of Biomedicine and Translational Medicine, University of Tartu, 50090 Tartu, Estonia;; ^h^Service de Médecine Intensive Réanimation, Hôpitaux Universitaires Henri Mondor, Assistance Publique-Hôpitaux de Paris, 94010 Créteil, France;; ^i^Groupe de Recherche Clinique Cardiovascular and Respiratory Manifestations of Acute Lung Injury and Sepsis (CARMAS), Faculté de santé de Créteil, Université Paris Est Créteil, 94010 Créteil Cedex, France;; ^j^Hypoxia and Lung, INSERM U1272, Avicenne Hospital, Assistance Publique-Hôpitaux de Paris, 93022 Bobigny, France;; ^k^Sorbonne Université, Hôpital Pitié Salpêtrière, Médecine Intensive Réanimation, Assistance Publique-Hôpitaux de Paris, 75013 Paris, France;; ^l^INSERM, UMRS 1166-iCAN, Institute of Cardiometabolism and Nutrition, 75013 Paris, France;; ^m^Internal Medicine Department, Lariboisière Hospital, Assistance Publique-Hôpitaux de Paris, University of Paris, 75010 Paris, France;; ^n^INSERM U1019–CNRS UMR9017, Center for Infection and Immunity of Lille, Institut Pasteur de Lille, University of Lille, 59000 Lille, France;; ^o^Centre Hospitalier Universitaire, de Lille, Pôle de Réanimation, Hôpital Roger Salengro Lille, 59000 Lille, France;; ^p^Etablissement Français du Sang, 93218 La Plaine Saint-Denis, France;; ^q^Interactions Hôte-Greffon-Tumeur et Ingénierie Cellulaire et Génique (RIGHT), INSERM, Etablissement Français du Sang, Université de Franche-Comté, 25000 Besançon, France;; ^r^Santé Ingéniérie Biologie St-Etienne (SAINBIOSE), INSERM U1059, University of Lyon, Université Jean Monnet Saint-Etienne, 42000 Saint-Étienne, France;; ^s^Etablissement Français du Sang, Auvergne-Rhône-Alpes, 42000 Saint-Étienne, France;; ^t^Department of Internal Medicine, Infanta Leonor University Hospital, 28031 Madrid, Spain;; ^u^Hospices Civils de Lyon, 69002 Lyon, France;; ^v^International Center of Research in Infectiology, Lyon University, INSERM U1111, CNRS UMR 5308, ENS, Ecole Nationale Supérieure, Université Claude Bernard Lyon 1 (UCBL), 69365 Lyon, France;; ^w^Joint Research Unit, Hospices Civils de Lyon-BioMérieux, Hospices Civils de Lyon, Lyon Sud Hospital, 69495 Pierre-Bénite, France;; ^x^National Referee Centre for Rheumatic, and Autoimmune and Systemic Diseases in Children, 69000 Lyon, France;; ^y^Immunopathology Federation Lyon Immunopathology Federation (LIFE), Hospices Civils de Lyon, 69002 Lyon, France;; ^z^Intensive Care Unit, Grand Hôpital de l’Est Francilien Site de Marne-La-Vallée, 77600 Jossigny, France;; ^aa^Microbes, Evolution, Phylogénie et Infection (MEPHI), Institut Hospitalo-Universitaire Méditerranée Infection, Institut de Recherche pour le Développement, Assistance Publique Hôpitaux de Marseille, Aix-Marseille Université, 13005 Marseille, France;; ^bb^Service d’Evaluation Médicale, Hôpitaux Universitaires de Marseille Assistance Publique Hôpitaux de Marseille, 13005 Marseille, France;; ^cc^Aix-Marseille University, School of Medicine, EA 3279, Centre d'Études et de Recherche sur les Services de Santé et la Qualité de vie (CEReSS)–Health Service Research and Quality of Life Center, 13385 Marseille, France;; ^dd^Laboratory of Clinical Immunology and Microbiology, Division of Intramural Research, National Institute of Allergy and Infectious Diseases (NIAID), NIH, Bethesda, MD 20892;; ^ee^Department of Pediatrics and Developmental Biology, Graduate School of Medical and Dental Sciences, Tokyo Medical and Dental University, Tokyo 113-8510, Japan;; ^ff^Department of Epidemiology, Infectious Disease Control and Prevention, Graduate School of Biomedical and Health Sciences, Hiroshima University, Hiroshima 734-8553, Japan;; ^gg^Hospital Universitari MútuaTerrassa, Universitat de Barcelona, 08193 Barcelona, Spain;; ^hh^Fundació Docència i Recerca Mutua Terrassa, 08221 Terrassa, Spain;; ^ii^Paris Cardiovascular Research Center (PARCC), INSERM, Université de Paris, 75015 Paris, France;; ^jj^Service d’Hématologie Biologique, Hôpital Lariboisière, Assistance Publique-Hôpitaux de Paris, Université de Paris, 75010 Paris, France;; ^kk^EA3518, Institut Universitaire d’Hématologie-Hôpital Saint Louis, Université de Paris, 75010 Paris, France;; ^ll^Réanimation Médicale et Toxicologique, Hôpital Lariboisière Assistance Publique-Hôpitaux de Paris, Université de Paris, INSERM, UMRS-1144, 75010 Paris, France;; ^mm^Laboratory of Immunobiology, Center for Clinical, Experimental Surgery, and Translational Research, Biomedical Research Foundation of the Academy of Athens, 11527 Athens, Greece;; ^nn^Service de Parasitologie-Mycologie, Groupe Hospitalier Pitié Salpêtrière, Assistance Publique-Hôpitaux de Paris, 75013 Paris, France;; ^oo^Institute for Systems Biology, Seattle, WA 98109;; ^pp^Primary Immunodeficiencies Group, Department of Microbiology and Parasitology, School of Medicine, University of Antioquia UdeA, 050010 Medellín, Colombia;; ^qq^Center for Autoimmune Disease Research, School of Medicine and Health Sciences, Universidad del Rosario, 110111 Bogotá, Colombia;; ^rr^Intensive Care Medicine, University Hospital of Gran Canaria Dr. Negrín, Canarian Health System, 35010 Las Palmas de Gran Canaria, Spain;; ^ss^Centro de Investigación Biomédica en Red (CIBER) de Enfermedades Respiratorias, Instituto de Salud Carlos III, 28029 Madrid, Spain;; ^tt^Department of Clinical Sciences, Universidad Fernando Pessoa Canarias, 35450 Las Palmas de Gran Canaria, Spain;; ^uu^CHemato-oncology Research Laboratory of Associazione italiana contro le leucemie-linfomi e mieloma, Diagnostic Departement, Azienda Socio Sanitaria Territoriale, Spedali Civili di Brescia, 25123 Brescia, Italy;; ^vv^Pediatric Department and Centro Tettamanti-European Reference Network PaedCan, EuroBloodNet, European Reference Network for Rare Hereditary Metabolic Disorders (MetabERN), University of Milano Bicocca, Fondazione Monza Brianza Bambino Mamma (MBBM), Ospedale San Gerardo, 20900 Monza, Italy;; ^ww^Department of Infectious Diseases, San Gerardo Hospital, University of Milano Bicocca, 20900 Monza, Italy;; ^xx^Pediatric Clinic, Fondazione Istituto di Ricovero e Cura a carattere scientifico (IRCCS) Policlinico San Matteo, Department of Clinical, Surgical, Diagnostic and Pediatric Sciences, University of Pavia, 27100 Pavia, Italy;; ^yy^National Institute of Allergy and Infectious Diseases (NIAID) Clinical Genomics Program, NIH, Bethesda, MD 20892;; ^zz^Department of Pharmacology and Molecular Therapeutics, Uniformed Services University of the Health Sciences, Bethesda, MD 20814;; ^aaa^Department of Pediatrics, British Columbia Children’s Hospital, University of British Columbia, Vancouver, BC V6H 0B3, Canada;; ^bbb^Primary Immunodeficiencies Group, University of Antioquia UdeA, 050010 Medellin, Colombia;; ^ccc^School of Microbiology, University of Antioquia UdeA, 050010 Medellin, Colombia;; ^ddd^Department of Nephrology and Transplantation, Necker University Hospital, Assistance Publique-Hôpitaux de Paris, 75743 Paris, France;; ^eee^Institut Necker Enfants Malades, INSERM U1151–CNRS UMR 8253, Université de Paris, 75015 Paris, France;; ^fff^Institute for Biomedical Research, Spanish National Research Council, 08036 Barcelona, Spain;; ^ggg^Institut d’Investigacions Biomèdiques August Pi i Sunyer, 08036 Barcelona, Spain;; ^hhh^Department of Paediatric Immunology and Pulmonology, Center for Primary Immunodeficiency Ghent, Jeffrey Modell Diagnosis and Research Center, Ghent University Hospital, 9000 Ghent, Belgium;; ^iii^Faculty of Medical Sciences, University “Goce Delchev,” Štip 2000, Republic of North Macedonia;; ^jjj^Institute of Public Health of the Republic of North Macedonia, Skopje 1000, Republic of North Macedonia;; ^kkk^Department of Molecular Biology and Genetics, Bilkent University, 06800 Ankara, Turkey;; ^lll^Meram Faculty of Medicine, Necmettin Erbakan University, 42080 Konya, Turkey;; ^mmm^Clinical Immunology Unit, Department of Pediatric Infectious Disease, Centre Hospitalier-Universitaire Ibn Roucshd, 20360 Casablanca, Morocco;; ^nnn^Laboratoire d’Immunologie Clinique, Inflammation et Allergie (LICIA), Faculty of Medicine and Pharmacy, Hassan II University, 20250 Casablanca, Morocco;; ^ooo^Center for Autoimmune Disease Research, School of Medicine and Health Sciences, Universidad del Rosario, 111211 Bogotá, Colombia;; ^ppp^Department of Immunology, CIC1408, Groupe sur l’Immunité des Muqueuses et des Agents Pathogènes (GIMAP) Centre International de Recherche en Infectiologie, INSERM U1111, University Hospital of Saint-Étienne, 42000 Saint-Étienne, France;; ^qqq^Department of Biosciences and Nutrition, Karolinska Institutet, 171 77 Stockholm, Sweden;; ^rrr^University of Lille, INSERM, Centre Hospitalier Universitaire de Lille, Institut Pasteur de Lille, U1011-European Genomic Institute for Diabetes (EGID), F-59000 Lille, France;; ^sss^The Genetics Institute and Genomics Center, Tel Aviv Sourasky Medical Center, 6423906 Tel Aviv, Israel;; ^ttt^Feinstein Institutes for Medical Research, Northwell Health, Manhasset, NY 11030;; ^uuu^Vita-Salute San Raffaele University, and Clinical Genomics, Istituto di Ricovero e Cura a Carattere Scientifico (IRCCS) Ospedale San Raffaele, 20132 Milan, Italy;; ^vvv^Diabetes Research Institute, Istituto di Ricovero e Cura a Carattere Scientifico (IRCCS) San Raffaele Scientific Institute, 20132 Milan, Italy;; ^www^Hematology and Bone Marrow Transplantation Unit, Istituto di Ricovero e Cura a Carattere Scientifico (IRCCS) Ospedale San Raffaele University Vita-Salute San Raffaele, 20132 Milano, Italy;; ^xxx^Department of Immunology, Institute of Biomedical Sciences, University of São Paulo, 05508-060 São Paulo, Brazil;; ^yyy^Fundación Rioja Salud, Centro de Investigación Biomédica de La Rioja, 26006 Logroño, Spain;; ^zzz^University of São Paulo, 05508-060 São Paulo, Brazil;; ^aaaa^Department of Neurology, Amsterdam UMC, Amsterdam Neuroscience, University of Amsterdam, Amsterdam, 1105 AZ, The Netherlands;; ^bbbb^Department of Internal Medicine II, Medical University of Innsbruck, 6020 Innsbruck, Austria;; ^cccc^Shupyk National Healthcare University of Ukraine, 04112 Kyiv, Ukraine;; ^dddd^Department of Children’s Diseases and Pediatric Surgery, I. Horbachevsky Ternopil National Medical University, 46022 Ternopil, Ukraine;; ^eeee^Department of Infection Control and Prevention, Medical Hospital, Tokyo Medical and Dental University, Tokyo 113-8655, Japan;; ^ffff^Intensive Care Medicine, Donostia University Hospital, Biodonostia Institute of Donostia, 20014 San Sebastián, Spain;; ^gggg^Centro de Investigación Biomédica en Red (CIBER) de Enfermedades Respiratorias, Instituto de Salud Carlos III, 28029 Madrid, Spain;; ^hhhh^Garvan Institute of Medical Research, Sydney, NWS 2010, Australia;; ^iiii^St Vincent’s Clinical School, Faculty of Medicine and Health, University of New South Wales, Sydney, NWS 2010, Australia;; ^jjjj^Sorbonne Université, INSERM U1136, Institut Pierre Louis d'Epidémiologie et de Santé Publique, Assistance Publique-Hôpitaux de Paris, Hôpital Pitié Salpêtrière, Service de Virologie, 75013 Paris, France;; ^kkkk^Translational Immunology Unit, Institut Pasteur, Université Paris Cité, 75015 Paris, France;; ^llll^Human Evolutionary Genetics Unit, Institut Pasteur, CNRS UMR 2000, 75015 Paris, France;; ^mmmm^Department of Human Genomics and Evolution, Collège de France, 75231 Paris, France;; ^nnnn^Department of Immunology, 2nd Faculty of Medicine, Charles University and University Hospital in Motol, 150 06 Prague, Czech Republic;; ^oooo^Department of Immunology, Dmitry Rogachev National Medical Research Center of Pediatric Hematology, Oncology and Immunology, Moscow, Russia 117997;; ^pppp^Department of Paediatrics and Adolescent Medicine, University of Hong Kong, Hong Kong 999077, China;; ^qqqq^Division of Geriatric Medicine, Tours University Medical Center, 37044 Tours, France;; ^rrrr^INSERM U1259, Morphogenèse et Antigénicité du VIH et des Virus des Hépatites (MAVIVH), Université de Tours, 37044 Tours, France;; ^ssss^Service de Bactériologie, Virologie et Hygiène Hospitalière, Centre Hospitalier Universitaire de Tours, 37044 Tours, France;; ^tttt^Department of Microbiology, Universidad de La Sabana, 250001 Chía, Colombia;; ^uuuu^Department of Critical Care Medicine, Clínica Universidad de La Sabana, 250001 Chía, Colombia;; ^vvvv^Service de Biologie Médicale, Centre Hospitalier Intercommunal Robert Ballanger, 93600 Aulnay-sous-Bois, France;; ^wwww^Laboratoire d’Immunologie, Hospices Civils de Lyon, Hôpital Edouard Herriot, 69437 Lyon, France;; ^xxxx^Centre International de Recherche en Infectiologie, INSERM U1111, CNRS, UMR5308, Ecole Normale Supérieure de Lyon, Université Claude Bernard Lyon 1, 69007 Lyon, France;; ^yyyy^EA 7426, Pathophysiology of Injury-Induced Immunosuppression, Université Claude Bernard Lyon 1, Hospices Civils de Lyon, BioMérieux, Hôpital Edouard Herriot, 69437 Lyon, France;; ^zzzz^Laboratory of Molecular Immunology, Rockefeller University, New York, NY 10065;; ^aaaaa^HHMI, Rockefeller University, New York, NY 10065;; ^bbbbb^Sackler Faculty of Medicine, Tel Aviv University, 6997801 Tel Aviv, Israel;; ^ccccc^Allergy and Clinical Immunology Unit, Department of Medicine, Tel Aviv Sourasky Medical Center, 6423906 Tel Aviv, Israel;; ^ddddd^Medical Intensive Care Unit, University Hospitals Leuven, 3000 Leuven, Belgium;; ^eeeee^Laboratory of Inborn Errors of Immunity, Department of Microbiology, Immunology and Transplantation, Katholieke Universiteit Leuven, 3000 Leuven, Belgium;; ^fffff^Department of Pediatrics, Jeffrey Modell Diagnostic and Research Network Center, University Hospitals Leuven, 3000 Leuven, Belgium;; ^ggggg^Department of Age-Related Healthcare, Tallaght University Hospital, Dublin D24 NR0A, Ireland;; ^hhhhh^Department of Medical Gerontology, School of Medicine, Trinity College Dublin, Dublin D08 W9RT, Ireland;; ^iiiii^Sharjah Institute for Medical Research, College of Medicine, University of Sharjah, 27272 Sharjah, United Arab Emirates;; ^jjjjj^Immunology Research Lab, College of Medicine, King Saud University, 11362 Riyadh, Saudi Arabia;; ^kkkkk^Sorbonne Université, INSERM, Centre d’Immunologie et des Maladies Infectieuses, 75013 Paris, France;; ^lllll^Cerba HealthCare, 92130 Issy-les-Moulineaux, France;; ^mmmmm^Department of Pediatrics, King Fahad Hospital of the University, Al Khobar 34445, Saudi Arabia;; ^nnnnn^College of Medicine, Imam Abdulrahman Bin Faisal University, Dammam 34212, Saudi Arabia;; ^ooooo^Department of Clinical Laboratory, Hospital Universitari de Bellvitge, The Bellvitge Biomedical Research Institute (IDIBELL), 08908 Barcelona, Spain;; ^ppppp^Department of Immunology, Hospital Universitari de Bellvitge, The Bellvitge Biomedical Research Institute (IDIBELL), 08908 Barcelona, Spain;; ^qqqqq^Department of Medicine, Division of Infectious Diseases, McGill University Health Centre, Montréal, QC H4A 3J1, Canada;; ^rrrrr^Infectious Disease Susceptibility Program, Research Institute of the McGill University Health Centre, Montréal, QC H4A 3J1, Canada;; ^sssss^Department of Clinical Immunology, Aarhus University Hospital, 8000 Aarhus, Denmark;; ^ttttt^Faculdades Pequeno Príncipe, Instituto de Pesquisa Pelé Pequeno Príncipe, 80250-200 Curitiba, Brazil;; ^uuuuu^Department of Medical Microbiology, University Medical Center Utrecht, 3584 CX Utrecht, The Netherlands;; ^vvvvv^Service de Médecine Interne, Hôpital Universitaire Jean-Verdier, Assistance Publique-Hôpitaux de Paris, 93140 Bondy, France;; ^wwwww^INSERM U1138, Centre de Recherche des Cordeliers, 75006 Paris, France;; ^xxxxx^School of Life Sciences, École Polytechnique Fédérale de Lausanne, 1015 Lausanne, Switzerland;; ^yyyyy^Precision Medicine Unit, Lausanne University Hospital and University of Lausanne, 1011 Lausanne, Switzerland;; ^zzzzz^Swiss Institute of Bioinformatics, 1015 Lausanne, Switzerland;; ^aaaaaa^Imagine Institute, Université de Paris, INSERM, UMR 1163, 75015 Paris, France;; ^bbbbbb^Yale Center for Genome Analysis, Yale School of Medicine, New Haven, CT 06511;; ^cccccc^Department of Genetics, Yale University School of Medicine, New Haven, CT 06520;; ^dddddd^Department of Neurosurgery, Yale University School of Medicine, New Haven, CT 06510;; ^eeeeee^Department of Medical Genetics, Acibadem University School of Medicine, 34750 Istanbul, Turkey;; ^ffffff^Laboratory of Human Genetics and Genomics, Rockefeller University, New York, NY 10065;; ^gggggg^Diabetes Center, University of California, San Francisco, CA 94143;; ^hhhhhh^Department of Hematology, Necker Hospital, Assistance Publique-Hôpitaux de Paris, 75015 Paris, France;; ^iiiiii^Neurometabolic Diseases Laboratory, The Bellvitge Biomedical Research Institute (IDIBELL), 08908 Barcelona, Spain;; ^jjjjjj^Centre for Biomedical Research on Rare Diseases (CIBERER) U759, Instituto de Salud Carlos III, 28029 Madrid, Spain;; ^kkkkkk^Catalan Institution of Research and Advanced Studies (ICREA), 08010 Barcelona, Spain;; ^llllll^Department of Infectious Diseases, Aarhus University Hospital, 8000 Aarhus, Denmark;; ^mmmmmm^Department of Biomedicine, Aarhus University, 8000 Aarhus, Denmark;; ^nnnnnn^ADMA Biologics Inc., Ramsey, NJ 07446;; ^oooooo^University of Bordeaux, INSERM, Bordeaux Population Health Center, UMR1219, F-33000 Bordeaux, France;; ^pppppp^Department of Neurology, Institute of Neurodegenerative Diseases, Bordeaux University Hospital, F-33000 Bordeaux, France;; ^qqqqqq^Institut Hospitalo-Universitaire Méditerranée Infection, Unité des Virus Émergents, Aix-Marseille University, Institut pour la Recherche et le Développment (IRD) 190, INSERM 1207, 13005 Marseille, France;; ^rrrrrr^Epidémiologie clinique du Centre d’Investigation Clinique (CIC-EP), INSERM CIC 1425, Hôpital Bichat, 75018 Paris, France;; ^ssssss^Université de Paris, Infection Antimicrobials Modelling Evolution (IAME), UMR 1137, INSERM, 75870 Paris, France;; ^tttttt^Département Epidémiologie, Biostatistiques et Recherche Clinique, Hôpital Bichat, Assistance Publique-Hôpitaux de Paris, 75018 Paris, France;; ^uuuuuu^Service de Réanimation Médicale et des Maladies Infectieuses, Hôpital Bichat, Assistance Publique-Hôpitaux de Paris, Nord Université de Paris, F-75018 Paris, France;; ^vvvvvv^Cohorte Constances Groupe Hospitalier Universitaire centre, Assistance Publique-Hôpitaux de Paris, Université de Paris, 94800 Villejuif, France;; ^wwwwww^Immunology Division, Genetics Department, Hospital Universitari Vall d’Hebron, Vall d’Hebron Research Institute, Universitat Autònoma de Barcelona, 08035 Barcelona, Spain;; ^xxxxxx^Département d’Immunologie, Assistance Publique-Hôpitaux de Paris, Hôpital Pitié-Salpétrière, 75015 Paris, France;; ^yyyyyy^Department of Internal Medicine, Hospital Universitari de Bellvitge, The Bellvitge Biomedical Research Institute (IDIBELL), 08908 Barcelona, Spain;; ^zzzzzz^IrsiCaixa AIDS Research Institute, 08916 Badalona, Spain;; ^aaaaaaa^Institute for Health Science Research Germans Trias i Pujol (IGTP), 08916 Badalona, Spain;; ^bbbbbbb^Department of Infectious Diseases and Immunity, University of Vic-Central University of Catalonia, 08500 Vic, Spain;; ^ccccccc^Catalan Institution of Research and Advanced Studies (ICREA), 08010 Barcelona, Spain;; ^ddddddd^Consorcio Centro de Investigación Biomédica en Red de Enfermedades Infecciosas (CIBERINFEC), Instituto de Salud Carlos III, 28029 Madrid, Spain;; ^eeeeeee^Service de Biologie Clinique and UMR-S 1176, Hôpital Foch, 92150 Suresnes, France;; ^fffffff^Department of Immunology, University Hospital of Gran Canaria Dr. Negrin, Canarian Health System, 35010 Las Palmas de Gran Canaria, Spain;; ^ggggggg^Department of Pathology and Laboratory Medicine, Perelman School of Medicine, University of Pennsylvania, Philadelphia, PA 19104;

**Keywords:** COVID-19, type I IFNs, autoantibodies, relative risk, infection fatality rate

## Abstract

Severe acute respiratory syndrome coronavirus 2 (SARS-CoV-2) infection fatality rate (IFR) doubles with every 5 y of age from childhood onward. Circulating autoantibodies neutralizing IFN-α, IFN-ω, and/or IFN-β are found in ∼20% of deceased patients across age groups, and in ∼1% of individuals aged <70 y and in >4% of those >70 y old in the general population. With a sample of 1,261 unvaccinated deceased patients and 34,159 individuals of the general population sampled before the pandemic, we estimated both IFR and relative risk of death (RRD) across age groups for individuals carrying autoantibodies neutralizing type I IFNs, relative to noncarriers. The RRD associated with any combination of autoantibodies was higher in subjects under 70 y old. For autoantibodies neutralizing IFN-α2 or IFN-ω, the RRDs were 17.0 (95% CI: 11.7 to 24.7) and 5.8 (4.5 to 7.4) for individuals <70 y and ≥70 y old, respectively, whereas, for autoantibodies neutralizing both molecules, the RRDs were 188.3 (44.8 to 774.4) and 7.2 (5.0 to 10.3), respectively. In contrast, IFRs increased with age, ranging from 0.17% (0.12 to 0.31) for individuals <40 y old to 26.7% (20.3 to 35.2) for those ≥80 y old for autoantibodies neutralizing IFN-α2 or IFN-ω, and from 0.84% (0.31 to 8.28) to 40.5% (27.82 to 61.20) for autoantibodies neutralizing both. Autoantibodies against type I IFNs increase IFRs, and are associated with high RRDs, especially when neutralizing both IFN-α2 and IFN-ω. Remarkably, IFRs increase with age, whereas RRDs decrease with age. Autoimmunity to type I IFNs is a strong and common predictor of COVID-19 death.

## Significance

There is growing evidence that preexisting autoantibodies neutralizing type I interferons (IFNs) are strong determinants of life-threatening COVID-19 pneumonia. It is important to estimate their quantitative impact on COVID-19 mortality upon SARS-CoV-2 infection, by age and sex, as both the prevalence of these autoantibodies and the risk of COVID-19 death increase with age and are higher in men. Using an unvaccinated sample of 1,261 deceased patients and 34,159 individuals from the general population, we found that autoantibodies against type I IFNs strongly increased the SARS-CoV-2 infection fatality rate at all ages, in both men and women. Autoantibodies against type I IFNs are strong and common predictors of life-threatening COVID-19. Testing for these autoantibodies should be considered in the general population.

There have already been more than 250 million severe acute respiratory syndrome coronavirus 2 (SARS-CoV-2) infections and at least 5 million deaths from COVID-19 worldwide. Interindividual clinical variability in the course of infection with SARS-CoV-2 is immense, ranging from silent infection in about 40% of cases to acute respiratory distress syndrome in ∼3% of cases (1–5). Death occurs in ∼1% of cases (6). Age is the strongest epidemiological predictor of COVID-19 death, with the risk of death doubling every 5 y of age from childhood onward (6, 7). Men are also at greater risk of death than women (5, 8). Based on previously identified inborn errors of type I interferon (IFN) immunity (9), the COVID Human Genetic Effort (10) has shown that type I IFN immunity is essential for protective immunity to respiratory infection with SARS-CoV-2 (11–14). We have reported that inborn errors of Toll-like receptor 3 (TLR3)-dependent type I IFN immunity can underlie life-threatening COVID-19 pneumonia in a small subset of patients (14). Biochemically deleterious mutations of eight genes were found in 23 patients with critical COVID-19 (3.5% of 659 patients), including 18 patients under 60 y old. Remarkably, four unrelated patients, aged 25 y to 50 y, had autosomal recessive (AR) deficiencies of IFNAR1 or IRF7, including three homozygotes (two for *IFNAR1* and one for *IRF7*) and one compound heterozygote (for *IRF7*). Three other patients with AR IFNAR1 or TBK1 deficiency were independently reported (15–17). The penetrance of those defects is unknown, but it is probably higher for AR than for autosomal dominant disorders. We then reported that X-linked recessive TLR7 deficiency accounted for 1.8% of cases of life-threatening COVID-19 in men under 60 y old (13, 18). The penetrance of this disorder is apparently high but incomplete, especially in children. Deficiencies of IFNAR1 and IRF7 blunt type I IFN immunity across cell types, whereas defects of the TLR3 and TLR7 pathway preferentially affect respiratory epithelial cells and plasmacytoid dendritic cells, respectively (13, 19).

We have also reported the presence of autoantibodies (auto-Abs) neutralizing high concentrations (10 ng/mL, with plasma diluted 1/10) of IFN-α2 and/or IFN-ω in about 10% of patients with critical COVID-19 pneumonia but not in individuals with asymptomatic or mild infection (12). This finding has already been replicated in 14 other cohorts (20–35). We then detected auto-Abs neutralizing lower, more physiological concentrations (100 pg/mL, with plasma diluted 1/10) of IFN-α2 and/or IFN-ω in 13.6% of patients with life-threatening COVID-19, and 18% of deceased patients (11). The proportion of male patients was greater in patients with auto-Abs than in patients without auto-Abs (11, 12). In addition, 1.3% of patients with critical COVID-19 had auto-Abs neutralizing IFN-β (10 ng/mL, with plasma diluted 1/10), most without auto-Abs neutralizing IFN-α2 or IFN-ω. The prevalence of auto-Abs neutralizing IFN-α2 and/or IFN-ω in the general population increased with age, from 0.18% for 10 ng/mL and 1% for 100 pg/mL in individuals between 18 y and 69 y old to 3.4% for 10 ng/mL and 6.3% for 100 pg/mL for individuals over 80 y old (11). The prevalence of auto-Abs against IFN-β did not increase with age. The crude odds ratios (ORs) for critical COVID-19 as opposed to asymptomatic or mild infection in auto-Ab carriers relative to noncarriers ranged from 3 to 67, depending on the type I IFNs recognized and the concentrations neutralized (11). At least 12 lines of evidence strongly suggest that auto-Abs against type I IFNs are strong determinants of COVID-19 death (Table 1). The specific impact of these auto-Abs on COVID-19 mortality according to age and sex remains unknown and is of major interest (52, 53), as both the prevalence of these auto-Abs and the risk of death increase with age and are higher in men. Here, using data reported by Bastard et al. (11), we estimated the relative risk of COVID-19 death (RRD) for type I IFN auto-Ab carriers relative to noncarriers and the corresponding SARS-CoV-2 infection fatality rate (IFR), by sex and age category.

**Table 1.** Lines of evidence suggesting that auto-Abs against type I IFNs are strong determinants of the risk of life-threatening COVID-19

Evidence	Examples	References
Auto-Abs against type I IFNs are present before SARS-CoV-2 infection	In patients for whom a sample collected before the COVID-19 pandemic was available, the auto-Abs were found to preexist infection.	(36)
	These auto-Abs are found in the uninfected general population, and their prevalence increases after the age of 65 y.	(11)
Auto-Abs are associated with COVID-19 severity	Patients with inborn errors underlying these auto-Abs from infancy onward (e.g., APS-1) have a very high risk of developing critical COVID-19 pneumonia.	(36)
	The population of patients with critical disease includes a higher proportion of individuals producing these auto-Abs than the population of patients with silent or mild infection (ORs depending on the nature, number, and concentrations of type I IFN neutralized).	(11)
	The results concerning the proportions of critical cases with auto-Abs against type I IFNs have already been replicated in >15 different cities (Americas, Europe, Asia).	(20, 23–35)
Auto-Abs against type I IFNs neutralize host antiviral activity	These auto-Abs neutralize the antiviral activity of type I IFNs against SARS-CoV-2 in vitro.	(12)
	These auto-Abs are found in vivo in the blood of SARS-CoV-2-infected patients, where they neutralize type I IFN.	(37)
	These auto-Abs are found in vivo in the respiratory tract of patients, where they neutralize type I IFN.	(38–40)
	A key virulence factor of SARS-CoV-2 in vitro is its capacity to impair type I IFN immunity.	(41)
	Animals with type I IFN deficiency develop critical disease, including animals treated with mAbs that neutralize type I IFNs.	(42)
Auto-Abs against cytokines are clinical phenocopies of the corresponding inborn errors	Patients with auto-Abs against type I IFNs are phenocopies of IFNAR1^−/−^, IFNAR2^−/−^, and IRF7^−/−^ patients with critical COVID-19 pneumonia.	(14)
	Patients with auto-Abs against IL-6, IL-17, GM-CSF, and type II IFN are phenocopies of the corresponding inborn errors and underlie staphylococcal disease, mucocutaneous candidiasis, nocardiosis, and mycobacterial diseases, respectively.	(43–51)

## Results

### Patients and Controls.

We estimated the RRD of individuals carrying auto-Abs neutralizing type I IFNs relative to noncarriers by Firth’s logistic regression, using large samples of 1,261 patients who died from COVID-19 and 34,159 individuals from the general population from whom samples were collected before the pandemic. In this study design, in which controls are sampled from the baseline population regardless of disease status, the ORs obtained by logistic regression approximate the relative risks (RRs) in the absence of the assumption of rare disease (54) (*SI Appendix*, *Supplementary Materials and Methods*). We confirmed that this statement remains valid in our study design, using Firth’s logistic regression by a simulation study (*SI Appendix*, *Supplementary Materials and Methods* and Fig. S1). For auto-Abs neutralizing low concentrations (100 pg/mL) of IFN-α2 and/or IFN-ω, we used 1,121 patients who died from COVID-19, and 10,778 individuals from the general population (Table 2). Assessments of auto-Abs neutralizing high concentrations (10 ng/mL) of IFN-α2 and/or IFN-ω were available for 1,094 deceased patients, and 34,159 individuals from the general population (Table 2). We also had assessments of auto-Abs neutralizing 10 ng/mL of IFN-β for a subsample of 636 deceased patients, and 9,126 individuals from the general population (Table 2). RRDs were estimated by means of Firth’s bias-corrected logistic regression, considering death as a binary outcome and adjusting for sex and age in six classes (20 y to 39 y, 40 y to 49 y, 50 y to 59 y, 60 y to 69 y, 70 y to 79 y, and ≥80 y). For assessment of the effect of age and sex on RRD, we added interaction terms between auto-Abs and age, and auto-Abs and sex terms to the logistic model (*Materials and Methods* and *SI Appendix*, *Supplementary Materials and Methods*).

**Table 2.** Characteristics of the general population cohort and of the cohort of patients who died from COVID-19

	Neutralization 100 pg/mL		Neutralization 10 ng/mL	
Characteristics	General population (*n* = 10,778)	Deceased patients (*n* = 1,121)	General population (*n* = 34,159)	Deceased patients (*n* = 1,094)
Male – no. (percent)	5,429 (50.4)*	821 (73.2)	17,859 (52.3)	805 (73.5)
Mean age ± SD* – years	62.3 ± 17.2	70.7 ± 13.0	52.7 ± 18.2	70.6 ± 13.1
Age distribution – no. (percent)				
20 y to 39 y	1,251 (11.6)	17 (1.5)	9,102 (26.6)	15 (1.4)
40 y to 49 y	1,459 (13.5)	43 (3.8)	5,403 (15.8)	47 (4.3)
50 y to 59 y	1,736 (16.1)	144 (12.8)	6,414 (18.9)	152 (13.9)
60 y to 69 y	2,475 (23.0)	307 (27.4)	6,881 (20.1)	289 (26.4)
70 y to 79 y	1,790 (16.6)	307 (27.4)	3,721 (10.9)	296 (27.1)
≥80 y	2,067 (19.2)	303 (27.0)	2,638 (7.7)	295 (27.0)
Auto-Ab – no. of carriers (percent)				
IFN-α2 and IFN-ω	65 (0.6)	102 (9.1)	45 (0.1)	75 (6.8)
IFN-α2 or IFN-ω	246 (2.3)	203 (18.1)	181 (0.5)	130 (11.9)
IFN-α2	151 (1.4)	140 (12.5)	117 (0.3)	118 (10.8)
IFN-ω	160 (1.5)	165 (14.7)	109 (0.3)	87 (8.0)
IFN-β^†^	NA	NA	24 (0.3)	6 (0.9)

NA, not available.

*Age is given in years and corresponds to age at the time of recruitment for members of the general population cohort (controls) and age at death for COVID-19 patients.

^†^IFN-β neutralization experiments were performed only for a concentration of 10 ng/mL, on 9,126 individuals (49.5% male, mean age 60.6 y) from the general population and 636 COVID-19 patients (71.1% male, mean age 72.9 y).

### RRD for Carriers of Auto-Abs Neutralizing Low Concentrations of Type I IFNs.

**Fig. 1.**
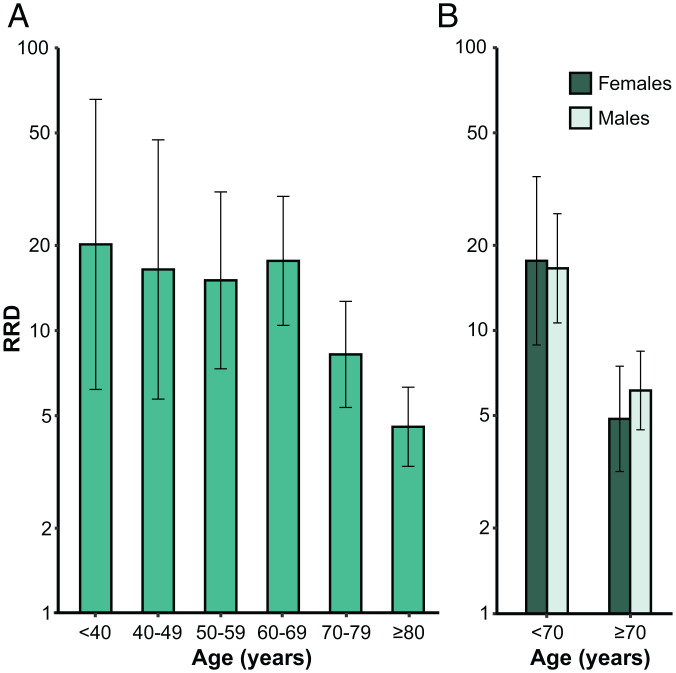
RRDs for individuals with auto-Abs neutralizing low concentrations of IFN-α2 or IFN-ω relative to individuals without such auto-Abs, by age and sex. RRDs are displayed on a logarithmic scale (*A*) for six age classes and (*B*) for male and female subjects under and over the age of 70 y. Vertical bars represent the 95% CI.

**Fig. 2.**
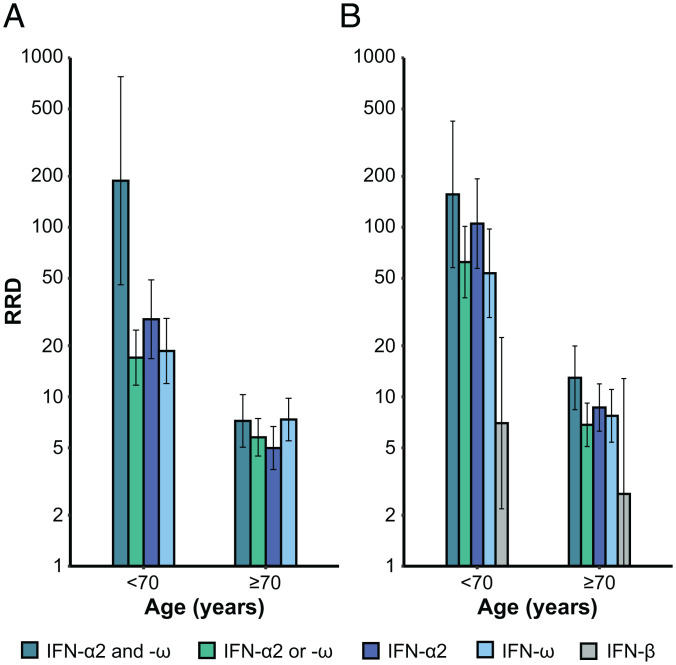
RRDs for individuals with auto-Abs neutralizing different combinations of type I IFNs relative to individuals without such auto-Abs, by age. RRDs are displayed on a logarithmic scale for individuals under and over 70 y of age with (*A*) auto-Abs neutralizing low concentrations of IFN-α2 and IFN-ω, IFN-α2 or IFN-ω, IFN-α2, and IFN-ω and (*B*) auto-Abs neutralizing high concentrations of IFN-α2 and IFN-ω, IFN-α2 or IFN-ω, IFN-α2, IFN-ω, and IFN-β, relative to individuals without such combinations of auto-Abs. Vertical bars represent the 95% CI.

We first estimated the RRD for individuals carrying auto-Abs neutralizing low concentrations of IFN-α2 or IFN-ω. As expected, increasing age and maleness were highly significantly associated with greater risk of COVID-19 death (*P* values ≤ 10^−16^; *SI Appendix*, Table S1). Different age classes were used to test the interaction with the presence of auto-Abs, and the best fit was obtained with a two-age class model (20 y to 69 y and ≥70 y; *SI Appendix*, Table S2) with a significant effect of the interaction term between auto-Abs and age (*P* value = 4 × 10^−6^). The RRD associated with auto-Abs did not vary significantly with sex (*P* value = 0.81). These interaction results are fully consistent with the distribution of RRD according to age (Fig. 1*A*) and sex (Fig. 1*B*), with a clear decrease in RRD after the age of 70 y, and no sex effect. Overall, the RRD for individuals carrying auto-Abs neutralizing IFN-α2 or IFN-ω decreased from 17.0 (95% CI: 11.7 to 24.7) before the age of 70 y to 5.8 (4.5 to 7.4) for individuals ≥70 y old (Fig. 2*A* and *SI Appendix*, Table S3). We then applied the same strategy to other combinations of auto-Abs neutralizing low concentrations of IFN, and observed similar age effects on RRDs (*SI Appendix*, Table S1). The presence of auto-Abs neutralizing both IFN-α2 and IFN-ω was associated with the highest RRD, estimated at 188.3 (45.8 to 774.4) for individuals under the age of 70 y and 7.2 (5.0 to 10.3) for those over 70 y old (Fig. 2*A* and *SI Appendix*, Table S3). We also estimated the population attributable fraction (PAF), to assess the proportion of COVID-19 deaths attributable to auto-Abs (*SI Appendix*, *Supplemental Materials and Methods*). Given the high RRD estimated for all combinations of auto-Abs neutralizing low concentrations of type I IFNs, the PAF was very close to the prevalence of these auto-Abs in deceased patients (*SI Appendix*, Table S3).

### RRD for Carriers of Auto-Abs Neutralizing High Concentrations of Type I IFNs.

We then estimated the RRD for the presence versus the absence of auto-Abs neutralizing high concentrations (10 ng/mL) of type I IFN. The effect of age on RRD was similar to that observed with auto-Abs neutralizing low concentrations of type I IFN, with the use of two age classes providing the best fit (*SI Appendix*, Tables S2 and S4), and a decrease of RRD with age (Fig. 2*B* and *SI Appendix*, Table S5). The RRD for carriers of IFN-α2 or IFN-ω auto-Abs decreased from 62.4 (38.4 to 101.3) before the age of 70 y to 6.8 (5.1 to 9.2) after the age of 70 y, whereas carriers of auto-Abs against both IFN-α2 and IFN-ω had the highest RRD, estimated at 156.5 (57.8 to 423.4) and 12.9 (8.4 to 19.9) for subjects <70 y and ≥70 y old, respectively (Fig. 2*B* and *SI Appendix*, Table S5). Individuals carrying auto-Abs neutralizing high concentrations of IFN-α2 and/or IFN-ω had a significantly higher RRD than individuals carrying only auto-Abs neutralizing low concentrations (*SI Appendix*, *Supplemental Materials and Methods*). This finding, consistent with the higher proportion of auto-Abs neutralizing high concentrations in deceased patients than in the general population (*SI Appendix*, Fig S2), suggests a more deleterious impact of auto-Abs neutralizing high concentrations of IFN-α2 and/or IFN-ω on COVID-19 outcomes. Finally, auto-Abs neutralizing high doses of IFN-β had the lowest RRD before 70 y (7.0 [2.2 to 22.4]), with no significant age-dependent association (*P* value = 0.37). The PAF for auto-Abs neutralizing high concentrations of type I IFNs was also close to the prevalence of these auto-Abs in deceased patients (*SI Appendix*, Table S5).

### IFR in Individuals Carrying Auto-Abs Neutralizing Low Concentrations of Type I IFNs.

**Fig. 3.**
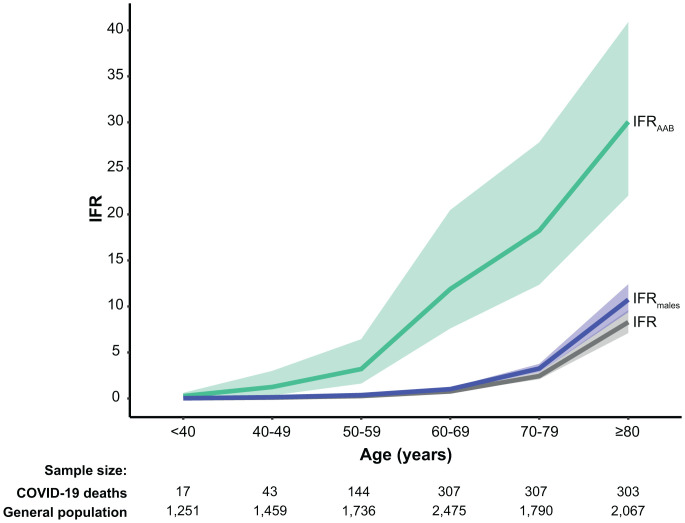
SARS-CoV-2 IFRs by age. IFRs are provided for the general population for both sexes (gray) and for males only (blue), from the data of O’Driscoll et al. (6); IFR_AAB_ (green) are shown for individuals carrying auto-Abs neutralizing low concentrations of IFN-α2 or IFN-ω. Auto-Abs against type I IFNs are associated with high RRDs and strongly increase the IFR, to a much greater extent than being male, and, by inference, than other common classical risk factors providing ORs of death similar to that for being male (around two), such as certain comorbid conditions, or the most significant common genetic variant on chromosome 3 (5).

**Fig. 4.**
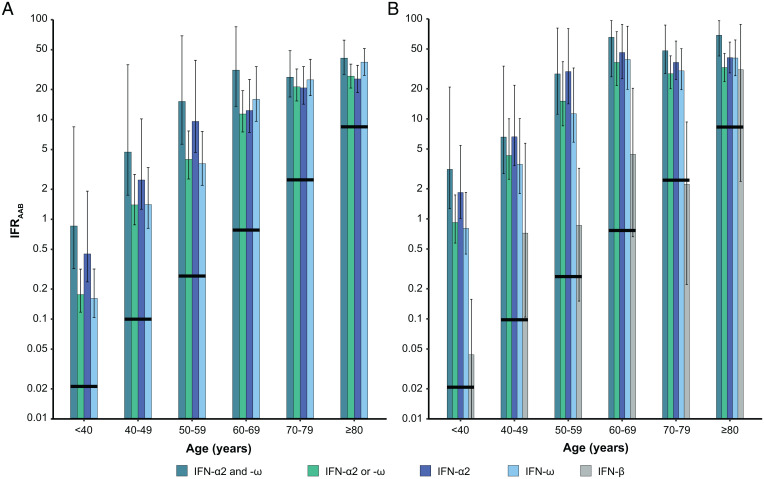
SARS-CoV-2 IFRs for carriers of various combinations of neutralizing auto-Abs, by age. IFR_AAB_ values (percent) are displayed, on a logarithmic scale, by age, for individuals with (*A*) auto-Abs neutralizing low concentrations of IFN-α2 and IFN-ω, IFN-α2 or IFN-ω, IFN-α2, and IFN-ω and (*B*) auto-Abs neutralizing high concentrations of IFN-α2 and IFN-ω, IFN-α2 or IFN-ω, IFN-α2, IFN-ω, and IFN-β. Vertical bars represent the 95% CI. Horizontal black lines represent the IFR provided by O’Driscoll et al. (6).

We then estimated the IFR in SARS-CoV-2–infected individuals carrying auto-Abs neutralizing low concentrations of type I IFNs (IFR_AAB_). According to Bayes’ theorem, IFR_AAB_ can be expressed as a function of the age-dependent prevalence of auto-Abs in deceased patients and in the general population together with the reported age-specific IFR (6) (*SI Appendix*). For all combinations of auto-Abs, the IFR_AAB_ was much higher than the overall IFR. Fig. 3 illustrates this much higher IFR for carriers of auto-Abs neutralizing low concentrations of IFN-α2 or IFN-ω; it exceeded 1% and 10% for subjects over the ages of 40 y and 60 y, respectively. Considering other combinations of auto-Abs, the highest IFR_AAB_ was observed for carriers of auto-Abs neutralizing both IFN-α2 and IFN-ω, reaching 40.5% (27.8 to 61.2) in individuals over 80 y old (Fig. 4*A* and *SI Appendix*, Table S6). IFR_AAB_ values were similar for all other combinations of auto-Abs. For example, the IFR_AAB_ for individuals carrying auto-Abs neutralizing either IFN-α2 or IFN-ω ranged from 0.17% (0.12 to 0.31) in individuals under 40 y old to 26.7% (20.3 to 35.2) in individuals over 80 y old. An exception was noted for the IFR_AAB_ of carriers of anti-IFN-α2 auto-Abs, which was 1.8 to 2.6 times higher than that for carriers of auto-Abs neutralizing IFN-α2 or IFN-ω in subjects under 60 y old. The IFR_AAB_ was also generally higher in male subjects than in female subjects, particularly in individuals carrying auto-Abs neutralizing both IFN-α2 and IFN-ω (∼2.7 times higher) (*SI Appendix*, Fig. S3).

### IFR in Individuals Carrying Auto-Abs Neutralizing High Concentrations of Type I IFNs.

The age-, sex-, and type I IFN–dependent patterns of IFR_AAB_ observed for carriers of auto-Abs neutralizing high concentrations of IFN-α2 and/or IFN-ω were similar to those previously obtained for carriers of auto-Abs neutralizing low concentrations of these molecules, but with higher values. For example, IFR_AAB_ ranged from 3.1% (1.3 to 20.8) before 40 y of age to 68.7% (42.5 to 95.8) in those over 80 y old for carriers of auto-Abs neutralizing high concentrations of both IFN-α2 and IFN-ω (Fig. 4*B* and *SI Appendix*, Table S7). IFR_AAB_ values were ∼5 times higher in male than in female subjects, across all age groups and auto-Abs combinations (*SI Appendix*, Fig. S4). For carriers of auto-Abs neutralizing IFN-β (tested only at high concentration), IFR_AAB_ was lower (by a factor of 6 to 71) than for individuals under the age of 80 y with auto-Abs neutralizing IFN-α2 and/or IFN-ω. It ranged from 0.04% (0.01 to 0.16) for individuals under the age of 40 y to 2.2% (0.2 to 9.3) for the 70- to 79-y age group. In the oldest age class, IFR_AAB_ was 31.0% (2.4 to 88.1), similar to that for carriers of auto-Abs against IFN-α2 or IFN-ω, albeit with a large confidence interval.

## Discussion

In this study, we took advantage of our previous data (11) to estimate RRDs associated with auto-Abs across age groups. We also confirmed, by a simulation study, that, in our design, ORs obtained by Firth’s logistic regression were reliable estimates of RR. In addition, we used IFR values previously reported for the general population (6) to estimate IFR_AAB_ under the plausible hypothesis that the prevalence of auto-Abs in the general population is a reliable estimation of the prevalence of auto-Abs in infected individuals (*SI Appendix*, *Supplemental Materials and Methods*). We report high RRDs for carriers of auto-Abs neutralizing type I IFNs, ranging from 2.6 for auto-Abs neutralizing IFN-β (high concentration) in subjects over 70 y old to >150 for auto-Abs neutralizing both IFN-α2 and IFN-ω in subjects under 70 y old. For all types of auto-Abs, RRDs were 3 to 26 times higher in subjects under 70 y old than in older individuals. This is consistent with the increasing prevalence of auto-Abs in the general population with age (∼1% under 70 y of age and >4% over 70 y of age), whereas the proportion of deceased patients with these auto-Abs is stable across age categories (∼15 to 20%). The lower RRD observed in the elderly may be partly explained epidemiologically, by the larger contribution of other mortality risk factors, such as comorbid conditions, which become more frequent with increasing age. At the cellular level, aging is associated with immunosenescence, which may contribute to a defective innate and adaptive response to SARS-CoV-2 infection, thereby conferring a predisposition to severe COVID-19 (55). At the molecular level, global type I IFN immunity in the blood (plasmacytoid dendritic cells) and respiratory tract (respiratory epithelial cells) has been shown to decline with age (56–59). These epidemiological, cellular, and molecular factors probably overlap. Thus, despite their increasing prevalence with age, auto-Abs against type I IFNs make a decreasing contribution to the risk of COVID-19 death with age, due to the progressive development of additional age-dependent risk factors, including other mechanisms of type I IFN deficiency. However, for the very same reasons, IFR_AAB_ increases dramatically with age in patients with auto-Abs, reaching 68.7% for carriers of auto-Abs neutralizing high concentrations of both IFN-α2 and IFN-ω.

RRD and IFR_AAB_ varied considerably with the IFNs recognized and the concentrations neutralized by auto-Abs. For combinations involving auto-Abs against IFN-α2 and/or IFN-ω, the neutralization of low concentrations was associated with a lower RRD and a lower IFR_AAB_ than the neutralization of high concentrations, suggesting that residual type I IFN activity may be beneficial in at least some patients. Blood IFN-α concentrations during acute asymptomatic or paucisymptomatic SARS-CoV-2 infection typically range from 1 pg/mL to 100 pg/mL (11). In addition, the presence of auto-Abs neutralizing both IFN-α2 and IFN-ω was associated with the highest RRD and IFR_AAB_ values. Interestingly, IFN-α2 and IFN-ω are encoded by two genes, *IFNA2* and *IFNW1*, that have been shown to have evolved under strong selective constraints (60), consistent with their neutralization being harmful to the host. In addition, patients with auto-Abs against IFN-α2 have been shown to neutralize all 13 IFN-α subtypes (11, 12), rendering any potential IFN-α redundancy inoperative (11, 12). Accordingly, the IFR_AAB_ values for carriers of auto-Abs against IFN-α2 were higher than those for carriers of auto-Abs against IFN-ω in subjects under 60 y of age. In older age groups, this difference tended to disappear, consistent with the lower impact of auto-Abs in the elderly, as discussed above. Finally, auto-Abs neutralizing IFN-β were less common, and associated with lower RRD and IFR_AAB_ values (by about one order of magnitude) than auto-Abs against IFN-α2 and/or IFN-ω, in all age groups except the over-80s. This less deleterious effect of auto-Abs neutralizing IFN-β is consistent with a mouse study showing that the blockade of IFN-β alone does not alter the early dissemination of lymphocytic choriomeningitis virus (61). Overall, auto-Abs against type I IFNs are associated with very high RRD and IFR values, and the magnitude of this effect appears to be much larger than that of other known common risk factors apart from age, such as maleness (Fig. 4), comorbidities, or the most significant common genetic variant on chromosome 3, all of which have been associated with life-threatening COVID-19 with ORs of about two (5).

Despite the lower prevalence of these auto-Abs in younger than in older individuals, the much higher IFR_AAB_ observed in individuals with these auto-Abs suggests that the testing of infected individuals in all age groups is warranted. Particular attention should be paid to patients, especially children, with known autoimmune or genetic conditions associated with the production of auto-Abs against type I IFNs. Early treatments could be provided (62), including monoclonal antibodies (63), new antiviral drugs, and/or IFN-β in the absence of auto-Abs against IFN-β (64, 65). Rescue treatment by plasma exchange is a therapeutic option in patients who already have pneumonia (36). A screening of uninfected elderly people could be considered, given that these auto-Abs are found in 4% of individuals over 70 y old. Carriers of auto-Abs should be vaccinated against SARS-CoV-2 as a priority, and should benefit from a booster, whatever their age, and, ideally, from a monitoring of their antibody response to the vaccine. They should not receive live-attenuated vaccines, including the yellow fever vaccine (YFV-17D) and anti-SARS-CoV-2 vaccines based on the YFV-17D backbone (66). In cases of SARS-CoV-2 infection, vaccinated patients should be closely monitored. As SARS-CoV-2 vaccination coverage increases and mortality due to COVID-19 decreases over time, it will be important to reevaluate the risk of fatal COVID-19 in vaccinated individuals with and without auto-Abs. It is currently unclear whether these auto-Abs impair antibody responses to vaccines, and whether a vaccine-triggered antibody response can overcome type I IFN deficiency in response to large or even medium-sized viral inocula. Finally, further investigations are required to determine the contribution of these auto-Abs to other severe viral diseases, and to elucidate the mechanisms underlying their development, which may be age dependent. In the meantime, auto-Abs against type I IFNs should be considered as a leading common predictor of life-threatening COVID-19, after age, as their detection appears to have a much greater predictive value for death, and, by inference, hospitalization and critical COVID-19, than sex, comorbidities, and common genetic variants (Fig. 3).

## Materials and Methods

### Study Design.

We enrolled 1,261 patients aged 20 y to 99 y old who died from COVID-19 pneumonia before SARS-CoV-2 vaccines became available, and 34,159 controls from the adult general population from whom samples were collected before the COVID-19 pandemic, as previously described (11). The experiments involving human subjects were performed in accordance with institutional, local, and national ethical guidelines. Approval was obtained from the French Ethics Committee “Comité de Protection des Personnes,” the French National Agency for Medicine and Health Product Safety, and the “Institut National de la Santé et de la Recherche Médicale,” in France (protocol C10-13, ID-RCB number 2010-A00634-35), and the Rockefeller University Institutional Review Board in New York (protocol JCA-0700). Participants were consented prior to sampling and collection of clinical data. Auto-Ab determinations were performed as described by Bastard et al. (11, 66), and were classified as neutralizing high concentrations (10 ng/mL) of IFN-α2, IFN-ω, or IFN-β, or low concentrations (100 pg/mL) of IFN-α2 or IFN-ω (*SI Appendix*, *Supplemental Materials and Methods*).

### RRDs and IFRs for Carriers of Neutralizing Autoantibodies.

We estimated the RRD in individuals carrying auto-Abs neutralizing type I IFNs relative to noncarriers, using large samples of patients who died from COVID-19 and of individuals from the general population. For each combination of auto-Abs, a Firth’s bias-corrected logistic regression model, including auto-Ab status, sex, and age, was fitted (*SI Appendix*, Table S1). For assessments of the effect of age and sex on the RRD due to auto-Abs, we added interaction terms between auto-Abs and sex, and auto-Abs and age (*SI Appendix*, *Supplemental Materials and Methods*). A similar Firth’s logistic regression model was used in the subsample of carriers of auto-Abs, to assess the deleteriousness of auto-Abs neutralizing high concentrations relative to those neutralizing low concentrations of type I IFNs (*SI Appendix*, *Supplemental Materials and Methods*). From the RRD, we calculated the PAF to assess the proportion of COVID-19 deaths attributable to auto-Abs. The PAF can be estimated as follows: P(auto-Abs/death) * (1 − 1/RRD) (67), where P(auto-Abs/death) is the prevalence of auto-Abs in deceased patients.

Our goal was also to estimate the fatality rate upon infection with SARS-CoV-2 (IFR) in unvaccinated subjects carrying auto-Abs against type I IFNs across age groups and sexes. To this end, we used the fatality rate upon infection with SARS-CoV-2 in the general unvaccinated population provided by O’Driscoll et al. (6). We estimated the IFR for carriers of neutralizing auto-Abs infected with SARS-CoV-2 (IFR_AAB_) following Bayes’ theorem, and using the age-dependent prevalence of auto-Abs in deceased patients and in the general population together with the reported age-specific IFR (6) as detailed in *SI Appendix*, *Supplemental Materials and Methods*.

## Supplementary Material

Supplementary File

## Data Availability

All the data are available in the manuscript or in the supporting information. Plasma, cells, and genomic DNA are available from J.-L.C. under a material transfer agreement (MTA) with The Rockefeller University or the Imagine Institute. Huh-7.5 cells are available on request from C.M.R. under an MTA with The Rockefeller University and Apath LLC. The materials and reagents used are almost exclusively commercially available and nonproprietary. Materials derived from human samples may be made available on request, subject to any underlying restrictions concerning such samples.

## References

[1] B. Hu, H. Guo, P. Zhou, Z. L. Shi, Characteristics of SARS-CoV-2 and COVID-19. Nat. Rev. Microbiol. 19, 141–154 (2021). 33024307 10.1038/s41579-020-00459-7 PMC7537588

[2] A. Telenti , After the pandemic: Perspectives on the future trajectory of COVID-19. Nature 596, 495–504 (2021). 34237771 10.1038/s41586-021-03792-w

[3] Q. Zhang, P. Bastard, A. Cobat, J. L. Casanova; COVID Human Genetic Effort, Human genetic and immunological determinants of critical COVID-19 pneumonia. Nature 603, 587–598 (2022). 35090163 10.1038/s41586-022-04447-0 PMC8957595

[4] S. Pei, T. K. Yamana, S. Kandula, M. Galanti, J. Shaman, Burden and characteristics of COVID-19 in the United States during 2020. Nature 598, 338–341 (2021). 34438440 10.1038/s41586-021-03914-4

[5] Q. Zhang ; COVID Human Genetic Effort, Life-threatening COVID-19: Defective interferons unleash excessive inflammation. Med (N Y) 1, 14–20 (2020). 33363283 10.1016/j.medj.2020.12.001 PMC7748410

[6] M. O’Driscoll , Age-specific mortality and immunity patterns of SARS-CoV-2. Nature 590, 140–145 (2021). 33137809 10.1038/s41586-020-2918-0

[7] A. T. Levin , Assessing the age specificity of infection fatality rates for COVID-19: Systematic review, meta-analysis, and public policy implications. Eur. J. Epidemiol. 35, 1123–1138 (2020). 33289900 10.1007/s10654-020-00698-1 PMC7721859

[8] P. Brodin, Immune determinants of COVID-19 disease presentation and severity. Nat. Med. 27, 28–33 (2021). 33442016 10.1038/s41591-020-01202-8

[9] C. J. A. Duncan, R. E. Randall, S. Hambleton, Genetic lesions of Type I interferon signalling in human antiviral immunity. Trends Genet. 37, 46–58 (2021). 32977999 10.1016/j.tig.2020.08.017 PMC7508017

[10] J. L. Casanova, H. C. Su; COVID Human Genetic Effort, A global effort to define the human genetics of protective immunity to SARS-CoV-2 infection. Cell 181, 1194–1199 (2020). 32405102 10.1016/j.cell.2020.05.016 PMC7218368

[11] P. Bastard ; HGID Lab; COVID Clinicians; COVID-STORM Clinicians; NIAID Immune Response to COVID Group; NH-COVAIR Study Group; Danish CHGE; Danish Blood Donor Study; St. James’s Hospital; SARS CoV2 Interest group; French COVID Cohort Study Group; Imagine COVID-Group; Milieu Intérieur Consortium; CoV-Contact Cohort; Amsterdam UMC Covid-19; Biobank Investigators; COVID Human Genetic Effort; CONSTANCES cohort; 3C-Dijon Study; Cerba Health-Care; Etablissement du Sang study group, Autoantibodies neutralizing type I IFNs are present in **∼**4% of uninfected individuals over 70 years old and account for **∼**20% of COVID-19 deaths. Sci. Immunol. 6, eabl4340 (2021). 34413139 10.1126/sciimmunol.abl4340 PMC8521484

[12] P. Bastard ; HGID Lab; NIAID-USUHS Immune Response to COVID Group; COVID Clinicians; COVID-STORM Clinicians; Imagine COVID Group; French COVID Cohort Study Group; Milieu Intérieur Consortium; CoV-Contact Cohort; Amsterdam UMC Covid-19 Biobank; COVID Human Genetic Effort, Autoantibodies against type I IFNs in patients with life-threatening COVID-19. Science 370, eabd4585 (2020). 32972996 10.1126/science.abd4585 PMC7857397

[13] T. Asano ; COVID Human Genetic Effort; COVID-STORM Clinicians; COVID Clinicians; Imagine COVID Group; French COVID Cohort Study Group; CoV-Contact Cohort; Amsterdam UMC Covid-; Biobank; NIAID-USUHS COVID Study Group, X-linked recessive TLR7 deficiency in ∼1% of men under 60 years old with life-threatening COVID-19. Sci. Immunol. 6, eabl4348 (2021). 34413140 10.1126/sciimmunol.abl4348 PMC8532080

[14] Q. Zhang ; COVID-STORM Clinicians; COVID Clinicians; Imagine COVID Group; French COVID Cohort Study Group; CoV-Contact Cohort; Amsterdam UMC Covid-19 Biobank; COVID Human Genetic Effort; NIAID-USUHS/TAGC COVID Immunity Group, Inborn errors of type I IFN immunity in patients with life-threatening COVID-19. Science 370, eabd4570 (2020). 32972995 10.1126/science.abd4570 PMC7857407

[15] S. Khanmohammadi, N. Rezaei, M. Khazaei, A. Shirkani, A case of autosomal recessive interferon alpha/beta receptor alpha chain (IFNAR1) deficiency with severe COVID-19. J. Clin. Immunol. 42, 19–24 (2021). 34713375 10.1007/s10875-021-01166-5 PMC8553400

[16] H. Abolhassani , Inherited IFNAR1 deficiency in a child with both critical COVID-19 pneumonia and multisystem inflammatory syndrome. J. Clin. Immunol., 10.1007/s10875-022-01215-7 (2022). PMC8798309 35091979

[17] A. Schmidt , TBK1 and TNFRSF13B mutations and an autoinflammatory disease in a child with lethal COVID-19. NPJ Genom. Med. 6, 55 (2021). 34210994 10.1038/s41525-021-00220-w PMC8249618

[18] H. Abolhassani , X-linked TLR7 deficiency underlies critical COVID-19 pneumonia in a male patient with ataxia-telangiectasia. J. Clin. Immunol. 42, 1–9 (2021). 34686943 10.1007/s10875-021-01151-y PMC8536475

[19] J. L. Casanova, L. Abel, Mechanisms of viral inflammation and disease in humans. Science 374, 1080–1086 (2021). 34822298 10.1126/science.abj7965 PMC8697421

[20] D. Goncalves , Antibodies against type I interferon: Detection and association with severe clinical outcome in COVID-19 patients. Clin. Transl. Immunology 10, e1327 (2021). 34429968 10.1002/cti2.1327 PMC8370568

[21] E. R. Shaw , Temporal dynamics of anti-type 1 interferon autoantibodies in COVID-19 patients. Clin. Infect. Dis., 10.1093/cid/ciab1002 (2021). PMC8689695 34875033

[22] E. Savvateeva , Microarray-based detection of antibodies against SARS-CoV-2 proteins, common respiratory viruses and Type I interferons. Viruses 13, 2553 (2021). 34960822 10.3390/v13122553 PMC8705234

[23] R. Koning, P. Bastard, J. L. Casanova, M. C. Brouwer, D. van de Beek; with the Amsterdam U.M.C. COVID-19 Biobank Investigators, Autoantibodies against type I interferons are associated with multi-organ failure in COVID-19 patients. Intensive Care Med. 47, 704–706 (2021). 33835207 10.1007/s00134-021-06392-4 PMC8034036

[24] J. Troya , Neutralizing autoantibodies to Type I IFNs in >10% of patients with severe COVID-19 pneumonia hospitalized in Madrid, Spain. J. Clin. Immunol. 41, 914–922 (2021). 33851338 10.1007/s10875-021-01036-0 PMC8043439

[25] M. G. P. van der Wijst ; UCSF COMET consortium, Type I interferon autoantibodies are associated with systemic immune alterations in patients with COVID-19. Sci. Transl. Med. 13, eabh2624 (2021). 34429372 10.1126/scitranslmed.abh2624 PMC8601717

[26] S. E. Vazquez , Neutralizing autoantibodies to Type I interferons in COVID-19 convalescent donor plasma. J. Clin. Immunol. 41, 1169–1171 (2021). 34009544 10.1007/s10875-021-01060-0 PMC8132742

[27] E. Y. Wang ; Yale IMPACT Team, Diverse functional autoantibodies in patients with COVID-19. Nature 595, 283–288 (2021). 34010947 10.1038/s41586-021-03631-y PMC13130511

[28] M. S. Abers , Neutralizing type-I interferon autoantibodies are associated with delayed viral clearance and intensive care unit admission in patients with COVID-19. Immunol. Cell Biol. 99, 917–921 (2021). 34309902 10.1111/imcb.12495 PMC8444766

[29] A. Chauvineau-Grenier , Autoantibodies neutralizing type I interferons in 20% of COVID-19 deaths in a French hospital. *J. Clin. Immunol.*, https://doi.org/10.1007/s10875-021-01203-3 (2022). 10.1007/s10875-021-01203-3 PMC8791677 35083626

[30] X. Solanich , Pre-existing autoantibodies neutralizing high concentrations of type I interferons in almost 10% of COVID-19 patients admitted to intensive care in Barcelona. J. Clin. Immunol. 41, 1733–1744 (2021). 34570326 10.1007/s10875-021-01136-x PMC8475877

[31] M. P. Raadsen , Interferon-α2 auto-antibodies in convalescent plasma therapy for COVID-19. J. Clin. Immunol. 42, 232–239 (2021). 34767118 10.1007/s10875-021-01168-3 PMC8586830

[32] S. E. Chang , New-onset IgG autoantibodies in hospitalized patients with COVID-19. Nat. Commun. 12, 5417 (2021). 34521836 10.1038/s41467-021-25509-3 PMC8440763

[33] C. G. K. Ziegler , Impaired local intrinsic immunity to SARS-CoV-2 infection in severe COVID-19. Cell 184, 4713–4733.e22 (2021). 34352228 10.1016/j.cell.2021.07.023 PMC8299217

[34] Y. Acosta-Ampudia ; CP-COVID-19 group, COVID-19 convalescent plasma composition and immunological effects in severe patients. J. Autoimmun. 118, 102598 (2021). 33524876 10.1016/j.jaut.2021.102598 PMC7826092

[35] R. Carapito , Identification of driver genes for critical forms of COVID-19 in a deeply phenotyped young patient cohort. Sci. Transl. Med. 14, eabj7521 (2022). 34698500 10.1126/scitranslmed.abj7521

[36] P. Bastard , Preexisting autoantibodies to type I IFNs underlie critical COVID-19 pneumonia in patients with APS-1. J. Exp. Med. 218, e20210554 (2021). 33890986 10.1084/jem.20210554 PMC8077172

[37] M. G. P. van der Wijst , Type I interferon autoantibodies are associated with systemic immune alterations in patients with COVID-19. Sci. Transl. Med. 13, eabh2624 (2021). 34429372 10.1126/scitranslmed.abh2624 PMC8601717

[38] I. E. Galani , Untuned antiviral immunity in COVID-19 revealed by temporal type I/III interferon patterns and flu comparison. Nat. Immunol. 22, 32–40 (2021). 33277638 10.1038/s41590-020-00840-x

[39] B. Sposito , The interferon landscape along the respiratory tract impacts the severity of COVID-19. Cell 184, 4953–4968.e16 (2021). 34492226 10.1016/j.cell.2021.08.016 PMC8373821

[40] J. Lopez , Early nasal type I IFN immunity against SARS-CoV-2 is compromised in patients with autoantibodies against type I IFNs. J. Exp. Med. 218, e20211211 (2021). 34357402 10.1084/jem.20211211 PMC8352718

[41] D. Blanco-Melo , Imbalanced host response to SARS-CoV-2 drives development of COVID-19. Cell 181, 1036–1045.e9 (2020). 32416070 10.1016/j.cell.2020.04.026 PMC7227586

[42] B. Israelow , Mouse model of SARS-CoV-2 reveals inflammatory role of type I interferon signaling. J. Exp. Med. 217, e20201241 (2020). 32750141 10.1084/jem.20201241 PMC7401025

[43] R. Döffinger , Autoantibodies to interferon-gamma in a patient with selective susceptibility to mycobacterial infection and organ-specific autoimmunity. Clin. Infect. Dis. 38, e10–e14 (2004). 14679469 10.1086/380453

[44] C. Höflich , Naturally occurring anti-IFN-gamma autoantibody and severe infections with *Mycobacterium cheloneae* and *Burkholderia cocovenenans*. Blood 103, 673–675 (2004). 12947000 10.1182/blood-2003-04-1065

[45] B. Kampmann , Acquired predisposition to mycobacterial disease due to autoantibodies to IFN-gamma. J. Clin. Invest. 115, 2480–2488 (2005). 16127458 10.1172/JCI19316 PMC1190367

[46] A. Puel , Recurrent staphylococcal cellulitis and subcutaneous abscesses in a child with autoantibodies against IL-6. J. Immunol. 180, 647–654 (2008). 18097067 10.4049/jimmunol.180.1.647

[47] A. Puel , Autoantibodies against IL-17A, IL-17F, and IL-22 in patients with chronic mucocutaneous candidiasis and autoimmune polyendocrine syndrome type I. J. Exp. Med. 207, 291–297 (2010). 20123958 10.1084/jem.20091983 PMC2822614

[48] C. L. Ku, C. Y. Chi, H. von Bernuth, R. Doffinger, Autoantibodies against cytokines: Phenocopies of primary immunodeficiencies? Hum. Genet. 139, 783–794 (2020). 32419033 10.1007/s00439-020-02180-0 PMC7272486

[49] K. Kisand , Chronic mucocutaneous candidiasis in APECED or thymoma patients correlates with autoimmunity to Th17-associated cytokines. J. Exp. Med. 207, 299–308 (2010). 20123959 10.1084/jem.20091669 PMC2822605

[50] L. B. Rosen , Nocardia-induced granulocyte macrophage colony-stimulating factor is neutralized by autoantibodies in disseminated/extrapulmonary nocardiosis. Clin. Infect. Dis. 60, 1017–1025 (2015). 25472947 10.1093/cid/ciu968 PMC4366584

[51] J. L. Casanova, L. Abel, Lethal infectious diseases as inborn errors of immunity: Toward a synthesis of the germ and genetic theories. Annu. Rev. Pathol. 16, 23–50 (2021). 32289233 10.1146/annurev-pathol-031920-101429 PMC7923385

[52] P. Bastard, Why do people die from COVID-19? Science 375, 829–830 (2022). 35201875 10.1126/science.abn9649

[53] P. Bastard, Q. Zhang, S. Y. Zhang, E. Jouanguy, J. L. Casanova, Type I interferons and SARS-CoV-2: From cells to organisms. Curr. Opin. Immunol. 74, 172–182 (2022). 35149239 10.1016/j.coi.2022.01.003 PMC8786610

[54] A. Morabia, T. Ten Have, J. R. Landis, Empirical evaluation of the influence of control selection schemes on relative risk estimation: The Welsh nickel workers study. Occup. Environ. Med. 52, 489–493 (1995). 7670625 10.1136/oem.52.7.489 PMC1128269

[55] J. M. Bartleson , SARS-CoV-2, COVID-19 and the ageing immune system. Nat Aging 1, 769–782 (2021). 34746804 10.1038/s43587-021-00114-7 PMC8570568

[56] M. V. Splunter , Plasmacytoid dendritic cell and myeloid dendritic cell function in ageing: A comparison between elderly and young adult women. PLoS One 14, e0225825 (2019). 31830086 10.1371/journal.pone.0225825 PMC6907850

[57] J. L. Schultze, A. C. Aschenbrenner, COVID-19 and the human innate immune system. Cell 184, 1671–1692 (2021). 33743212 10.1016/j.cell.2021.02.029 PMC7885626

[58] G. R. Stark, J. E. Darnell Jr., The JAK-STAT pathway at twenty. Immunity 36, 503–514 (2012). 22520844 10.1016/j.immuni.2012.03.013 PMC3909993

[59] C. A. Pierce , Natural mucosal barriers and COVID-19 in children. JCI Insight 6, 148694 (2021). 33822777 10.1172/jci.insight.148694 PMC8262299

[60] J. Manry , Evolutionary genetic dissection of human interferons. J. Exp. Med. 208, 2747–2759 (2011). 22162829 10.1084/jem.20111680 PMC3244034

[61] C. T. Ng , Blockade of interferon Beta, but not interferon alpha, signaling controls persistent viral infection. Cell Host Microbe 17, 653–661 (2015). 25974304 10.1016/j.chom.2015.04.005 PMC4432251

[62] D. C. Vinh ; COVID Human Genetic Effort, Harnessing Type I IFN immunity against SARS-CoV-2 with early administration of IFN-β. J. Clin. Immunol. 41, 1425–1442 (2021). 34101091 10.1007/s10875-021-01068-6 PMC8186356

[63] D. M. Weinreich ; Trial Investigators, REGN-COV2, a neutralizing antibody cocktail, in outpatients with Covid-19. N. Engl. J. Med. 384, 238–251 (2021). 33332778 10.1056/NEJMoa2035002 PMC7781102

[64] P. Bastard , Interferon-β therapy in a patient with incontinentia pigmenti and autoantibodies against Type I IFNs infected with SARS-CoV-2. J. Clin. Immunol. 41, 931–933 (2021). 33763778 10.1007/s10875-021-01023-5 PMC7990897

[65] P. D. Monk ; Inhaled Interferon Beta COVID-19 Study Group, Safety and efficacy of inhaled nebulised interferon beta-1a (SNG001) for treatment of SARS-CoV-2 infection: A randomised, double-blind, placebo-controlled, phase 2 trial. Lancet Respir. Med. 9, 196–206 (2021). 33189161 10.1016/S2213-2600(20)30511-7 PMC7836724

[66] P. Bastard , Auto-antibodies to type I IFNs can underlie adverse reactions to yellow fever live attenuated vaccine. J. Exp. Med. 218, e20202486 (2021). 33544838 10.1084/jem.20202486 PMC7871457

[67] M. von Cube, J. F. Timsit, M. Schumacher, E. Motschall, M. Schumacher, Quantification and interpretation of attributable mortality in core clinical infectious disease journals. Lancet Infect. Dis. 20, e299–e306 (2020). 32916101 10.1016/S1473-3099(20)30485-0

