# Supplementary material for: The risk of COVID-19 death is much greater and age dependent with type I IFN autoantibodies
Source: Proc Natl Acad Sci U S A. 2022 May 16;119(21):e2200413119. doi: 10.1073/pnas.2200413119 (PMC9173764; doi:10.1073/pnas.2200413119)
Supplement: Supplementary File [file pnas.2200413119.sapp.pdf]

PNAS
www.pnas.org

Supplementary Information for

The risk of COVID-19 death is much greater and age-dependent with type I IFN autoantibodies.

Jérémy Manry, Paul Bastard, Adrian Gervais, Tom Le Voyer, Jérémie Rosain, Quentin Philippot, Eleftherios Michailidis, Hans-Heinrich Hoffmann, Shohei Eto, Marina Garcia-Prat, Lucy Bizien, Alba Parra-Martínez, Rui Yang, Liis Haljasmägi, Mélanie Migaud, Karita Särekannu, Julia Maslovskaja, Nicolas de Prost, Yacine Tandjaoui-Lambiotte, Charles-Edouard Luyt, Blanca Amador-Borrero, Alexandre Gaudet, Julien Poissy, Pascal Morel, Pascale Richard, Fabrice Cognasse, Jesús Troya, Sophie Trouillet-Assant, Alexandre Belot, Kahina Saker, Pierre Garçon, Jacques G. Rivière, Jean-Christophe Lagier, Stéphanie Gentile, Lindsey B. Rosen, Elana Shaw, Tomohiro Morio, Junko Tanaka, David Dalmau, Pierre-Louis Tharaux, Damien Sene, Alain Stepanian, Bruno Mégarbane, Vasiliki Triantafyllia, Arnaud Fekkar, James R. Heath, José Luis Franco, Juan-Manuel Anaya, Jordi Solé-Violán, Luisa Imberti, Andrea Biondi, Paolo Bonfanti, Riccardo Castagnoli, Ottavia M. Delmonte, Yu Zhang, Andrew L. Snow, Steven M. Holland, Catherine M. Biggs, Marcela Moncada-Vélez, Andrés Augusto Arias, Lazaro Lorenzo, Soraya Boucherit, Dany Anglicheau, Anna M. Planas, Filomeen Haerynck, Sotirija Duvlis, Tayfun Ozcelik, Sevgi Keles, Ahmed A. Bousfiha, Jalila El Bakkouri, Carolina Ramirez-Santana, Stéphane Paul, Qiang Pan-Hammarström, Lennart Hammarström, Annabelle Dupont, Alina Kurolap, Christine N. Metz, Alessandro Aiuti, Giorgio Casari, Vito Lampasona, Fabio Ciceri, Lucila A. Barreiros, Elena Dominguez-Garrido, Mateus Vidigal, Mayana Zatz, Diederik van de Beek, Sabina Sahanic, Ivan Tancevski, Yurii Stepanovskyy, Oksana Boyarchuk, Yoko Nukui, Miyuki Tsumura, Loreto Vidaur, Stuart G. Tangye, Sonia Burrel, Darragh Duffy, Lluis Quintana-Murci, Adam Klocperk, Nelli Y. Kann, Anna Shcherbina, Yu-Lung Lau, Daniel Leung, Matthieu Coulongeat, Julien Marlet, Rutger Koning, Luis Felipe Reyes, Angélique Chauvineau-Grenier, Fabienne Venet, Guillaume Monneret, Michel C. Nussenzweig, Romain Arrestier, Idris Boudhabhay, Hagit Baris-Feldman, David Hagin, Joost Wauters, Isabelle Meyts, Adam H. Dyer, Sean P. Kennelly, Nollaig M. Bourke, Rabih Halwani, Fatemeh Saheb Sharif-Askari, Karim Dorgham, Jérome Sallette, Souad Mehlal Sedkaoui, Suzan AlKhater, Raúl Rigo-Bonnin, Francisco Morandeira, Lucie Roussel, Donald C. Vinh, Christian Erikstrup, Antonio Condino-Neto, Carolina Prando, Anastasiia Bondarenko, András N. Spaan, Laurent Gilardin, Jacques Fellay, Stanislas Lyonnet, Kaya Bilguvar , Richard P. Lifton, Shrikant Mane, HGID Lab, COVID Clinicians, COVID-STORM Clinicians, NIAID Immune Response to COVID Group, NH-COVAIR Study Group, Danish CHGE, Danish Blood Donor Study, St. James's Hospital, SARS CoV2 Interest group, French COVID Cohort Study Group, Imagine COVID-Group, The Milieu Intérieur Consortium, CoV-Contact Cohort, Amsterdam UMC Covid-19 Biobank Investigators, COVID Human Genetic Effort, CP-COVID-19 Group, CONSTANCES cohort, 3C-Dijon Study, Cerba Health-Care, Etablissement du Sang study group, Mark S. Anderson, Bertrand Boisson,

Vivien Béziat, Shen-Ying Zhang, Evangelos Andreakos, Olivier Hermine, Aurora Pujol, Pärt Peterson, Trine H. Mogensen, Lee Rowen, James Mond, Stéphanie Debette, Xavier de Lamballerie, Charles Burdet, Lila Bouadma, Marie Zins, Pere Soler-Palacin, Roger Colobran, Guy Gorochov, Xavier Solanich, Sophie Susen, Javier Martinez-Picado, Didier Raoult, Marc Vasse, Peter K. Gregersen, Lorenzo Piemonti, Carlos Rodríguez-Gallego, Luigi D. Notarangelo, Helen C. Su, Kai Kisand, Satoshi Okada, Anne Puel, Emmanuelle Jouanguy, Charles M. Rice, Pierre Tiberghien, Qian Zhang, Jean-Laurent Casanova, Laurent Abel, Aurélie Cobat

Jérémy Manry; Jean-Laurent Casanova; Aurélie Cobat
Emails: jeremy.manry@inserm.fr; jean-laurent.casanova@mail.rockefeller.edu; aurelie.cobat@inserm.fr

This PDF file includes:

Supplementary text
Figures S1 to S4
Tables S1 to S7
SI References
Lists of consortia members

Supplementary Information Text

Supplementary Materials and Methods

Autoantibodies neutralizing type-I IFN. Auto-Ab determinations were performed as described by Bastard *et al.*(1, 2). Auto-Ab titers were determined by ELISA (manual or automated). The blocking activity of anti-IFN-α2, anti-IFN-ω and anti-IFN-β auto-Abs was determined on the basis of reporter luciferase activity. Briefly, HEK293T cells were transfected with a plasmid containing the firefly luciferase gene under the control of the human ISRE promoter in the pGL4.45 backbone and a plasmid constitutively expressing the *Renilla* luciferase for normalization (pRL-SV40). Cells were either left unstimulated or were stimulated with IFN-α2 and IFN-ω at a concentration of 10 ng/mL ('high' *i.e.* supraphysiological concentration) or 100 pg/mL ('low', *i.e.* more physiological concentration), or with IFN-β at a concentration of 10 ng/mL ('high concentration') for 16 hours at 37°C. We have already shown that auto-Abs neutralizing IFN-α2 are also able to neutralize most of the other 12 subtypes of IFN-α(1).

Relative risks of fatal COVID-19. We estimated the relative risk of death (RRD) in patients carrying auto-Abs neutralizing IFN-α2 and/or IFN-ω, or auto-Abs neutralizing IFN-β relative to patients without such auto-Abs, using large samples of patients who died from COVID-19 and of individuals from the general population. In this study design, in which controls are sampled from the baseline at-risk population regardless of disease status, odds ratios (ORs) provide an approximation for relative risks (RRs) in the absence of the assumption of a rare disease(3). All analyses were performed with Firth's bias-corrected logistic regression (4, 5). This method provides bias-reduction for small sample size, rare events, and yields finite and consistent estimates even in case of separation that may for example occur when a given combination of auto-Abs are observed in deceased patients and not in the general population for a given age class of a given gender. For each combination of auto-Abs, a Firth's logistic regression model, including auto-Ab status, sex and age in six classes (20-39, 40-49, 50-59, 60-69, 70-79, ≥80 years, Table S1), was fitted with the logistf package of R software (https://CRAN.R-project.org/package=logistf). For assessments of the effect of age and sex on the RRD due to auto-Abs, we added first order auto-Abs*sex and auto-Abs*age interaction terms to the Firth logistic regression model. For the auto-Abs*age interaction term, age was considered in either six (as defined above) or two (20-69 and ≥70 years) classes, and the model providing the best fit was selected based on the Akaïke information criterion for Firth's penalized partial likelihood (AICF). Nagashima and Sato used the AICF only in Cox regression models(6). We therefore adapted the AICF for use in logistic regression models by fixing the coefficients obtained with our Firth logistic regression models to a classic logistic regression framework with the fix.coef function implemented in R and extracting the resulting AICF (Table S2). *P* values were obtained in penalized likelihood-ratio tests.

We further investigated whether the risk of COVID-19 death was significantly higher for carriers of auto-Abs neutralizing high concentrations (10 ng/mL) of type I IFNs than for carriers of auto-Abs neutralizing low concentrations (100 pg/mL). For each combination of auto-Abs, we selected deceased individuals and individuals from the general population carrying auto-Abs neutralizing low concentrations of the given combination. In these subsamples of auto-Ab carriers, we tested for an association between auto-Abs neutralizing high concentrations of type I IFNs and death, by Firth's logistic regression, as described above. Analyses were systematically adjusted for age and sex and included an auto-Abs*age interaction term. Given the small sample size, we considered only two classes for age (20-69 and ≥70 years) (Fig. S2).

Simulation study. We assessed the validity of the RR approximation based on the OR obtained by Firth's logistic regression in our case-cohort design by simulation studies. We simulated a general population aged 20 to 100 years with equal number of individuals in the age classes 20-39, 40-49, 50-59, 60-69, 70-79, ≥80 years. We randomly assigned auto-Ab status to each individual with a probability equal to the estimated prevalence of auto-Abs for the corresponding age class in the general population. We used estimates of the prevalence of auto-Abs neutralizing low concentrations of IFN-α2 or IFN-ω, or of the prevalence of auto-Abs neutralizing low concentrations of IFN-α2 and IFN-ω (as provided in Table S6). We further infected the population and randomly assigned "deceased" status with a probability depending on auto-Abs status, age class, the age-

specific RRD for auto-Ab carriers, the age-specific prevalence of auto-Abs in the general population and the age-specific infection fatality rate (IFR) in the general population. For each specific age class, we defined:

$$P(Death/\text{ auto-Abs}) = RRD \times P(Death/\overline{auto\text{-}Abs}), \text{and}$$

$$P(Death/\overline{auto\text{-}Abs}) = \frac{IFR}{1 - P(auto\text{-}Abs) + RRD \times P(auto\text{-}Abs)}$$

IFR and P(auto-Abs) were taken from Table S6, according to the age of the individual. RRDs for individuals <70 years and ≥70 years were taken from Table S3. We randomly sampled 1,200 deceased individuals. From an independent general population simulated with the same parameters, we randomly sampled 10,000 individuals (corresponding to a general population sampled before the pandemic). We performed Firth's logistic regression on the simulated samples of the 1,200 deceased individuals and the 10,000 individuals from the general population using the same covariates as described above. We generated a total of 10,000 replicates for each combination of auto-Abs. The results are presented in Fig. S1. They confirm that the OR estimated by the age-adjusted Firth's logistic regression model in our design is a valid estimator of the RRD.

IFR for carriers of neutralizing autoantibodies. We estimated the IFR for carriers of neutralizing auto-Abs (IFR_{AAB}) infected with SARS-CoV-2, using Bayes' theorem, as follows:

$$IFR_{AAB} = P(Death/auto\text{-}Abs) = \frac{P(Death) \times P(auto\text{-}Abs/Death)}{P(auto\text{-}Abs)}$$

Where P(Death) is the general SARS-CoV-2 IFR as estimated by O'Driscoll *et al.*(7), P(auto-Abs/Death) is the prevalence of auto-Abs in deceased patients, and P(auto-Abs) is the prevalence of neutralizing auto-Abs in our general population sample. This later prevalence is a reasonable estimation of the prevalence of auto-Abs in infected individuals (regardless of their COVID-19 phenotypes) with respect to the reported prevalence of auto-Abs in asymptomatic and pauci-symptomatic SARS-CoV-2 infected subjects(1) which represents the large majority of infected individuals. IFR_{AAB} was then calculated for each auto-Ab combination and by age class. The IFR_{AAB} and P(auto-Abs) were estimated for the six age classes defined above, but P(auto-Abs/Death) was estimated for two age classes only (20-69 and ≥70 years) (Table S6, Table S7). Indeed, consistent with our RRD estimations for fatal COVID-19 in patients carrying auto-Abs *versus* non-carriers, the best-fit model for the effect of age on the prevalence of auto-Abs in deceased patients was obtained with the two age classes 20-69 and ≥70 years. The prevalence of neutralizing auto-Abs in the general population were estimated by Agresti-Coull adjustment, to avoid null values(8). The confidence intervals of the IFR_{AAB} were estimated by Monte-Carlo simulation. We estimated the empirical distribution of IFR_{AAB}, by randomly drawing values for IFR, P(auto-Abs/Death) and P(auto-Abs) based on their observed means and variances, assuming a normal distribution, and recomputing IFR_{AAB}. We simulated 10,000 replicates for which IFR_{AAB} (%) lay within the (0, 100] interval, and determined empirical 95% confidence intervals.

Fig. S1.

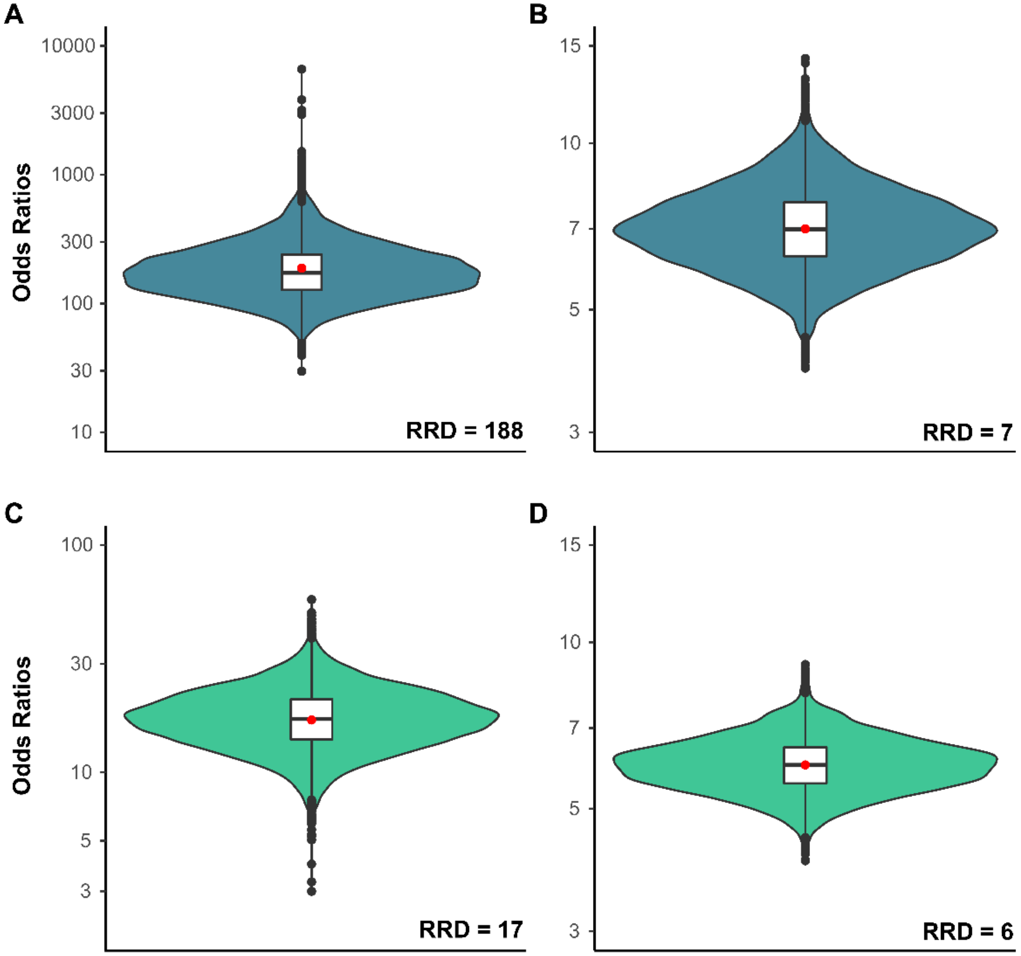

Violin plots of odds ratios obtained in the simulation study with the age-dependent prevalence of auto-Abs and RRD parameters, as estimated in our study. ORs were obtained by Firth's logistic regression analysis of 10,000 replicates of simulated deceased patients and individuals from the general population carrying auto-Abs neutralizing low concentrations of (1) IFN-α2 and IFN-ω with an RRD fixed at 188 in individuals <70 years of age (A), and with an RRD fixed at 7 in individuals ≥70 years of age (B), or (2) IFN-α2 or IFN-ω with an RRD fixed at 17 in individuals <70 years of age (C), and with an RRD fixed at 6 in individuals ≥70 years of age (D). Boxplots show the minimum and maximum simulated OR values, median, first and third quartiles on a logarithmic scale. The red dots represent the fixed RRD.

Fig. S2.

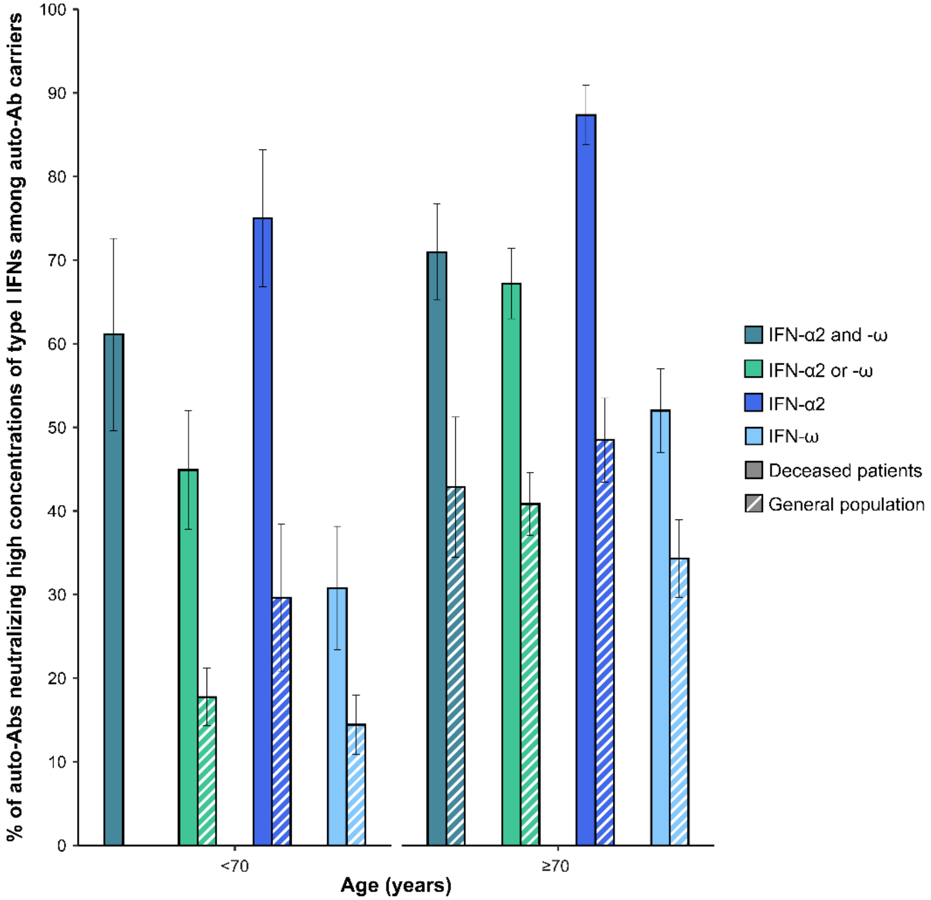

Sample size:								
Deceased patients	18	49	28	39	62	125	87	100
General population	0	124	27	97	35	169	99	105

Percentage of carriers of various combinations of auto-Abs neutralizing high concentrations of type I IFNs among all carriers of the same combinations of auto-Abs, by age. The analysis was restricted to auto-Ab carriers among deceased patients (plain bars) and in the general population (hatched bars). Auto-Abs neutralizing high concentrations of type I IFNs also neutralize low concentrations of type I IFNs. By fitting Firth's logistic regression models, we confirmed that the risk of death was significantly higher for individuals carrying auto-Abs neutralizing high concentrations than for those carrying auto-Abs neutralizing only low concentrations of type I IFNs, with P values of 0.05, 2.4×10^{-8}, 7.7×10^{-7} and 5.5×10^{-5} for auto-Abs neutralizing IFN-α and -ω, IFN-α or -ω, IFN-α, and IFN-ω, respectively.

Fig. S3.

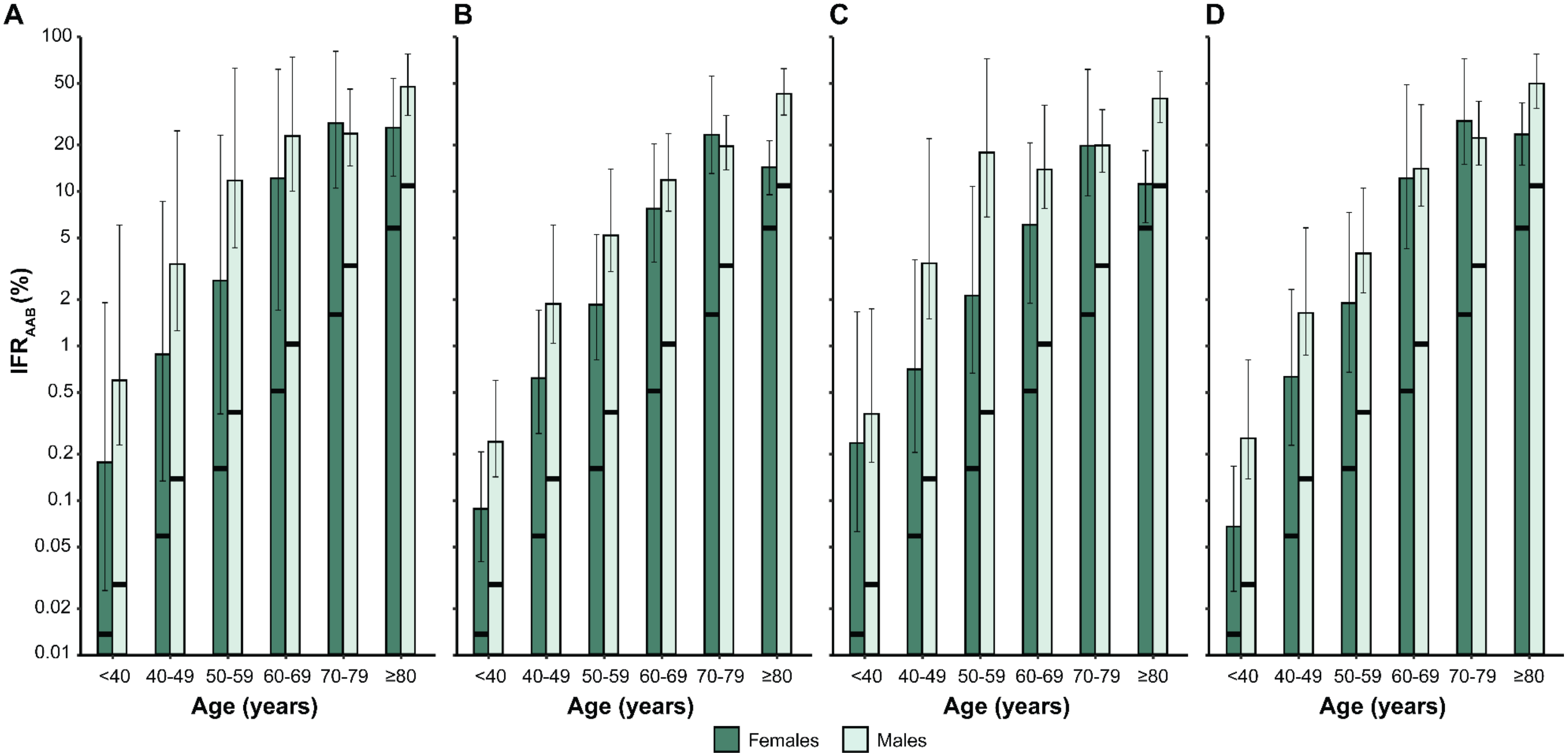

SARS-CoV-2 infection fatality rates for carriers of various combinations of auto-Abs (IFR_{AAB}) neutralizing low IFN concentrations, by age and sex. IFR_{AAB} are displayed, by age and sex, for individuals with auto Abs neutralizing low concentrations of (A) IFN α2 and IFN ω, (B) IFN α2 or IFN ω, (C) IFN α2, and (D) IFN ω. Vertical bars represent the 95% CI. Horizontal black lines represent the IFR reported by O'Driscoll et al. (7)

Fig. S4.

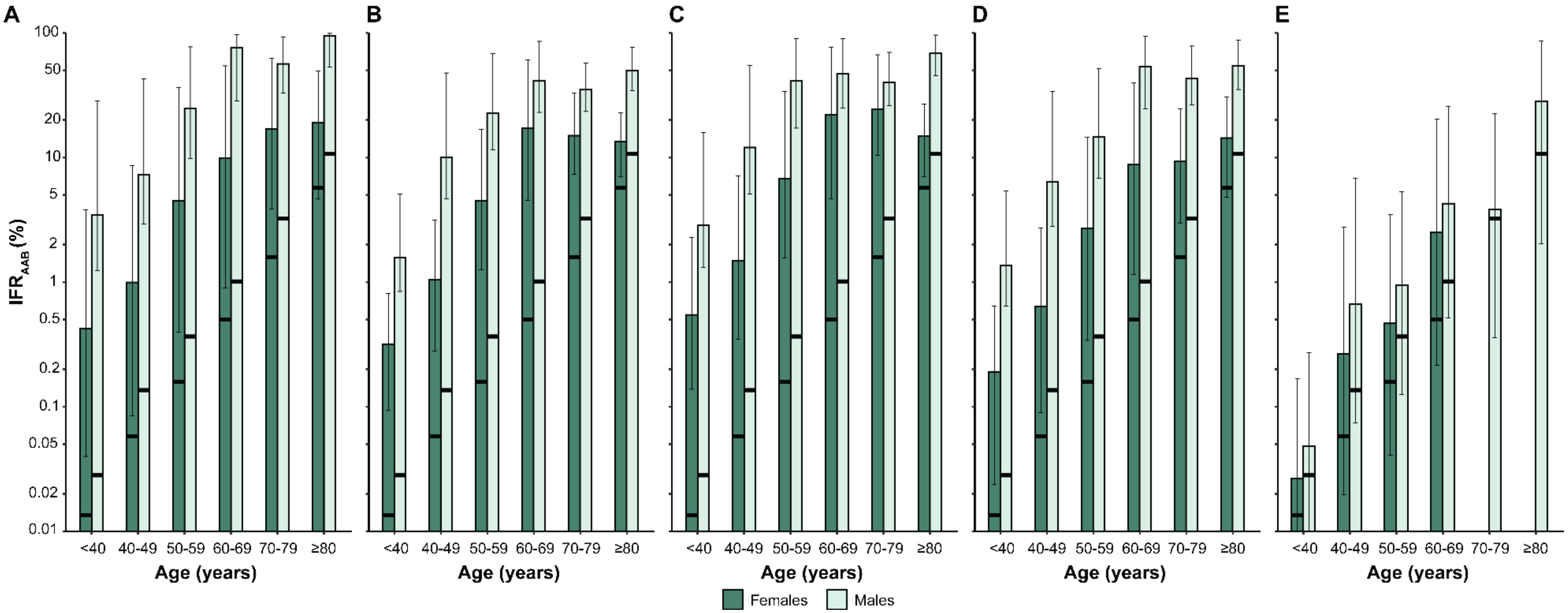

SARS-CoV-2 infection fatality rates for carriers of various combinations of auto-Abs (IFR$_{AAB}$) neutralizing high IFN concentrations, by age and sex. IFR$_{AAB}$ are displayed, by age and sex, for individuals with auto Abs neutralizing high concentrations of (A) IFN α2 and IFN ω, (B) IFN α2 or IFN ω, (C) IFN α2, (D) IFN ω and (E) IFN-β. Vertical bars represent the 95% CI. Horizontal black lines represent the IFR reported by O'Driscoll et al. (7) No woman over the age of 70 years carrying auto-Abs neutralizing IFN-β was identified among the patients who had died from COVID-19.

Table S1. Best fitting model for multivariate analysis according to various combinations of auto-Abs neutralizing low concentrations of type I IFNs

		IFN-α2 and IFN-ω			IFN-α2 or IFN-ω			IFN-α2			IFN-ω		
**Covariate**		**β (SE)**	**RRD**	***P* value**	**β (SE)**	**RRD**	***P* value**	**β (SE)**	**RRD**	***P* value**	**β (SE)**	**RRD**	***P* value**
**Sex**	**Female *vs.* Male**	-0.90 (0.07)	0.41	$<10^{-16}$	-0.93 (0.07)	0.40	$<10^{-16}$	-0.92 (0.07)	0.40	$<10^{-16}$	-0.92 (0.07)	0.40	$<10^{-16}$
**Age (years)**	**20-39 years**	ref	ref	-	ref	ref	-	ref	ref	-	ref	ref	-
	**40-49 years**	0.82 (0.30)	2.27	0.0041	0.83 (0.29)	2.29	0.0029	0.77 (0.29)	2.17	0.0056	0.84 (0.29)	2.31	0.0027
	**50-59 years**	1.82 (0.27)	6.16	10^{-16}	1.84 (0.26)	6.28	$<10^{-16}$	1.79 (0.26)	6.02	10^{-16}	1.82 (0.26)	6.19	$<10^{-16}$
	**60-69 years**	2.21 (0.26)	9.15	$<10^{-16}$	2.21 (0.25)	9.09	$<10^{-16}$	2.14 (0.25)	8.52	$<10^{-16}$	2.24 (0.25)	9.36	$<10^{-16}$
	**70-79 years**	2.51 (0.26)	12.27	$<10^{-16}$	2.54 (0.26)	12.70	$<10^{-16}$	2.48 (0.24)	11.98	$<10^{-16}$	2.53 (0.26)	12.50	$<10^{-16}$
	**≥80 years**	2.32 (0.26)	10.22	$<10^{-16}$	2.30 (0.26)	9.97	$<10^{-16}$	2.27 (0.25)	9.72	$<10^{-16}$	2.31 (0.26)	10.10	$<10^{-16}$
**auto-Abs**	**yes *vs.* no**	5.25 (0.71)	189.68[a]	$<10^{-16}$	2.82 (0.19)	16.71[a]	$<10^{-16}$	3.32 (0.27)	27.78[a]	$<10^{-16}$	2.92 (0.22)	18.57[a]	$<10^{-16}$
**auto-Abs*Age[a]**	**≥70 *vs.* <70**	-3.30 (0.73)	7.03[b]	8×10^{-10}	-1.05 (0.23)	5.85[b]	4×10^{-6}	-1.72 (0.31)	4.96[b]	6×10^{-9}	-0.92 (0.27)	7.41[b]	0.0005

Age-stratified analysis using Firth's logistic regression model integrating sex and auto-Abs*Age (<70 and ≥70 years) as covariates.

β, Relative risks of COVID-19 death (RRD) and *P* values for each covariate are displayed. Male sex, and <40 years age-class were used as reference.

NA: not applicable; SE: standard error, ref: reference.

[a]RRD for <70y.o. individuals displaying auto-Abs neutralizing IFN relative to those who do not as reference, computed as $\exp(\beta_{auto\text{-}Abs})$.

[b]RRD for individuals ≥70 y.o. individuals displaying auto-Abs neutralizing IFN relative to those who do not as reference, the interaction term 'auto-Abs*Age' was calculated as follows: $\exp(\beta_{auto\text{-}Abs} + \beta_{auto\text{-}Abs*Age})$.

Table S2. Model selection for the age effect of type I IFN auto-Abs on RRD based on the Aikake Information Criterion for Firth's penalized partial likelihood (AICF)

Neutralized IFN	Dose	AICF.6[a]	AICF.2[b]
IFN-α2 and -ω	100 pg/mL	680.92	**676.69**
IFN-α2 or -ω		1063.74	**1060.59**
IFN-α2		844.08	**840.37**
IFN-ω		939.02	**934.72**
IFN-α2 and -ω	10 ng/mL	533.82	**533.10**
IFN-α2 or -ω		796.76	**791.93**
IFN-α2		747.42	**742.17**
IFN-ω		595.11	**595.65**
IFN-β		69.91	**71.71**

The age effect of type I IFN auto-Abs on RRD was estimated through the interaction term auto-Abs*age with age considered in two (Age.2) or six (Age.6) classes.

Age.2 corresponds to [20, 70) and ≥70 years old classes; Age.6 corresponds to 20-39, 40-49, 50-59, 60-69, 70-79, ≥80 years old classes.

The best fitting models were selected according to the smallest AICF values, and are shown in bold. When ΔAICF < 2, we chose the most parsimonious model, thus displaying the smaller number of parameters (i.e. with 2 age classes only), as usually done.

[a]AICF obtained using the following logistic model: Death ~ auto-Abs status + Sex + Age.6 + auto-Abs*Age.6.

[b]AICF obtained using the following logistic model: Death ~ auto-Abs status + Sex + Age.6 + auto-Abs*Age.2.

Table S3. Relative risks of COVID-19 death (RRDs) associated with auto-Abs neutralizing low concentrations of various combinations of type I IFNs, by age

IFN neutralized	Age class	*P* value	RRD	RRD lower limit of 95% CI	RRD upper limit of 95% CI	P(auto-Abs/Death)[a] (%)	PAF[b] (%)
IFN-α2 and -ω	20-69 years	$<10^{-16}$	188.32	45.79	774.41	6.46	6.42
	≥70 years	$<10^{-16}$	7.20	5.04	10.30	11.31	9.74
IFN-α2 or -ω	20-69 years	$<10^{-16}$	17.01	11.69	24.75	15.26	14.37
	≥70 years	$<10^{-16}$	5.76	4.47	7.44	20.49	16.94
IFN-α2	20-69 years	$<10^{-16}$	28.66	16.78	48.96	10.18	9.82
	≥70 years	$<10^{-16}$	4.98	3.72	6.68	14.43	11.53
IFN-ω	20-69 years	$<10^{-16}$	18.62	11.97	28.98	11.55	10.93
	≥70 years	$<10^{-16}$	7.34	5.50	9.79	17.38	15.01

Age-stratified analysis with Firth's logistic regression model integrating sex and age classes (20-39, 40-49, 50-59, 60-69 for patients <70 years old; and 70-79 and ≥80 for patients ≥70 years old) as covariates. RRDs are displayed for individuals under and over 70 years of age.
95% CI: 95% confidence interval.
[a]Prevalence of auto-Abs among patients dying from COVID-19
[b]Population attributable fraction, i.e. quantification of the proportion of deaths attributable to auto-Abs against type I IFNs, approximated by P(auto-Abs/Death) * (1-1/RRD), as described by von Cube et al. (7). The higher the RRD, the closer the PAF to the prevalence of auto-Abs in deceased individuals.

Table S4. Best fitting model for multivariate analysis according to various combinations of auto-Abs neutralizing high concentrations of type I IFNs

Covariate		IFN-α2 and IFN-ω			IFN-α2 or IFN-ω			IFN-α2			IFN-ω			IFN-β		
		β (SE)	RRD	*P* value	β (SE)	RRD	*P* value	β (SE)	RRD	*P* value	β (SE)	RRD	*P* value	β (SE)	RRD	*P* value
**Sex**	**Female *vs* Male**	-0.84 (0.07)	0.43	$<10^{-16}$	-0.86 (0.07)	0.42	$<10^{-16}$	-0.86 (0.07)	0.42	$<10^{-16}$	-0.85 (0.07)	0.43	$<10^{-16}$	-1.07 (0.12)	0.34	$<10^{-16}$
**Age (years)**	**20-39 years**	ref	ref	-	ref	ref	-	ref	ref	-	ref	ref	-	ref	ref	-
	**40-49 years**	1.60 (0.29)	4.98	2×10^{-9}	1.63 (0.29)	5.10	10^{-9}	1.61 (0.30)	5.00	2×10^{-9}	1.62 (0.29)	5.07	10^{-9}	0.53 (0.49)	1.70	0.27
	**50-59 years**	2.55 (0.27)	12.84	$<10^{-16}$	2.60 (0.27)	13.43	$<10^{-16}$	2.59 (0.27)	13.27	$<10^{-16}$	2.57 (0.27)	13.12	$<10^{-16}$	2.06 (0.41)	7.87	6×10^{-11}
	**60-69 years**	3.09 (0.26)	22.08	$<10^{-16}$	3.12 (0.26)	22.56	$<10^{-16}$	3.10 (0.26)	22.14	$<10^{-16}$	3.12 (0.26)	22.66	$<10^{-16}$	2.56 (0.40)	12.88	$<10^{-16}$
	**70-79 years**	3.74 (0.26)	41.93	$<10^{-16}$	3.79 (0.26)	44.47	$<10^{-16}$	3.77 (0.26)	43.45	$<10^{-16}$	3.77 (0.26)	43.38	$<10^{-16}$	3.07 (0.40)	21.56	$<10^{-16}$
	**≥80 years**	4.11 (0.25)	60.80	$<10^{-16}$	4.12 (0.26)	61.44	$<10^{-16}$	4.10 (0.26)	60.55	$<10^{-16}$	4.13 (0.26)	62.00	$<10^{-16}$	NA	NA	NA
**auto-Abs**	**yes vs no**	5.05 (0.50)	156.99[a]	$<10^{-16}$	4.11 (0.24)	60.80[a]	$<10^{-16}$	4.63 (0.31)	102.79[a]	$<10^{-16}$	3.96 (0.30)	52.70[a]	$<10^{-16}$	1.93 (0.59)	6.91[a]	0.004
**auto-Abs*Age[a]**	**<70 vs ≥70**	-2.51 (0.55)	12.68[b]	2×10^{-7}	-2.19 (0.29)	6.81[b]	10^{-14}	-2.48 (0.35)	8.62[b]	5×10^{-14}	-1.94 (0.35)	7.61[b]	4×10^{-8}	-0.84 (1.00)	2.97[b]	0.37

Age-stratified analysis using Firth's logistic regression model integrating sex and auto-Abs*Age (<70 and ≥70 years) as covariates.

β, Relative risks of COVID-19 death (RRD) and P values for each covariate are displayed. Male sex, and <40 years age-classes were used as reference.

NA: not applicable; SE: standard error, ref: reference.

[a]RRD for <70y.o. individuals displaying auto-Abs neutralizing IFN relative to those who do not as reference, computed as $\exp(\beta_{auto\text{-}Abs})$.

[b]RRD for individuals ≥70 y.o. individuals displaying auto-Abs neutralizing IFN relative to those who do not as reference, the interaction term 'auto-Abs*Age' was calculated as follows: $\exp(\beta_{auto\text{-}Abs} + \beta_{auto\text{-}Abs*Age})$.

Table S5. Relative risks of COVID-19 death (RRDs) associated with auto-Abs neutralizing high concentrations of various combinations of type I IFNs, by age

IFN neutralized	Age class	*P* value	RRD	RRD lower limit of 95% CI	RRD upper limit of 95% CI	P(auto-Abs/Death)[a] (%)	PAF[b] (%)
IFN-α2 and -ω	20-69 years	$<10^{-16}$	156.47	57.82	423.37	4.97	4.94
	≥70 years	$<10^{-16}$	12.93	8.39	19.94	8.46	7.81
IFN-α2 or -ω	20-69 years	$<10^{-16}$	62.36	38.37	101.35	9.74	9.59
	≥70 years	$<10^{-16}$	6.83	5.08	9.17	13.71	11.70
IFN-α2	20-69 years	$<10^{-16}$	105.10	57.18	193.20	8.75	8.66
	≥70 years	$<10^{-16}$	8.64	6.26	11.92	12.52	11.07
IFN-ω	20-69 years	$<10^{-16}$	53.58	29.37	97.74	5.96	5.85
	≥70 years	$<10^{-16}$	7.72	5.39	11.04	9.64	8.39
IFN-β	20-69 years	0.004	6.98	2.18	22.36	1.62	1.39
	≥70 years	0.229	2.67	0.56	12.81	0.51	0.32

Age-stratified analysis with Firth's logistic regression model integrating sex and age classes (20-39, 40-49, 50-59, 60-69 for patients <70 years old; and 70-79 and ≥80 for patients ≥70 years old) as covariates. RRDs are displayed for individuals under and over 70 years of age.
95% CI: 95% confidence interval.
[a]Prevalence of auto-Abs in patients dying from COVID-19.
[b]Population attributable fraction, i.e. quantification of the proportion of deaths attributable to auto-Abs against type I IFNs, approximated by P(auto-Abs/Death) * (1-1/RRD), as described by von Cube et al. (7). The higher the RRD, the closer the PAF to the prevalence of auto-Abs in deceased individuals.

Table S6. ***SARS-CoV-2 infection fatality rates for individuals with auto-Abs (IFR_{AAB}) neutralizing low concentration of type I IFNs.***

IFN neutralized	Age group	IFR_{AAB}[a] (%)	IFR_{AAB} lower limit of 95% CI	IFR_{AAB} upper limit of 95% CI	IFR[b] (%)	P(auto-Abs)[c] (%)	P(auto-Abs/Death)[d] (%)
IFN-α2 and IFN-ω	20-39 years	0.84	0.31	8.28	0.02	0.16	6.46
	40-49 years	4.63	1.71	34.71	0.10	0.14	6.46
	50-59 years	14.89	5.55	67.61	0.27	0.11	6.46
	60-69 years	30.64	13.31	83.29	0.77	0.16	6.46
	70-79 years	26.04	16.49	48.01	2.44	1.06	11.31
	≥80 years	40.47	27.82	61.20	8.29	2.32	11.31
IFN-α2 or IFN-ω	20-39 years	0.17	0.12	0.31	0.02	1.83	15.26
	40-49 years	1.37	0.86	2.77	0.10	1.09	15.26
	50-59 years	3.91	2.49	7.53	0.27	1.03	15.26
	60-69 years	11.14	7.36	19.07	0.77	1.05	15.26
	70-79 years	20.85	15.04	31.46	2.44	2.40	20.49
	≥80 years	26.66	20.28	35.20	8.29	6.37	20.49
IFN-α2	20-39 years	0.44	0.23	1.88	0.02	0.48	10.18
	40-49 years	2.43	1.24	9.95	0.10	0.41	10.18
	50-59 years	9.38	4.59	38.49	0.27	0.29	10.18
	60-69 years	12.07	7.28	24.73	0.77	0.65	10.18
	70-79 years	20.36	13.98	33.26	2.44	1.73	14.43
	≥80 years	25.02	18.23	34.25	8.29	4.78	14.43
IFN-ω	20-39 years	0.16	0.10	0.31	0.02	1.51	11.55
	40-49 years	1.38	0.80	3.24	0.10	0.82	11.55
	50-59 years	3.55	2.15	7.46	0.27	0.86	11.55
	60-69 years	15.65	9.41	33.23	0.77	0.56	11.55
	70-79 years	24.52	17.03	39.35	2.44	1.73	17.38
	≥80 years	36.84	27.17	50.46	8.29	3.91	17.38

[a]COVID-19 fatality rate for individuals with auto-Abs.
[b]IFR provided by O'Driscoll *et al.*(7)
[c]Prevalence of auto-Abs in the general population.
[d]Prevalence of auto-Abs in patients dying from COVID-19.
95% CI: 95% confidence interval.

Table S7. SARS-CoV-2 infection fatality rates for individuals with auto-Abs (IFR_{AAB}) neutralizing high concentration of type I IFNs.

IFN neutralized	Age group	IFR_{AAB}[a] (%)	IFR_{AAB} lower limit of 95% CI	IFR_{AAB} upper limit of 95% CI	IFR[b] (%)	P(auto-Abs)[c] (%)	P(auto-Abs/Death)[d] (%)
IFN-α2 and IFN-ω	20-39 years	3.13	1.26	20.82	0.02	0.03	4.97
	40-49 years	6.58	2.83	33.71	0.10	0.07	4.97
	50-59 years	28.18	11.12	80.90	0.27	0.05	4.97
	60-69 years	65.49	26.37	96.22	0.77	0.06	4.97
	70-79 years	48.03	28.40	86.69	2.44	0.43	8.46
	≥80 years	68.65	42.52	95.81	8.29	1.02	8.46
IFN-α2 or IFN-ω	20-39 years	0.92	0.57	1.74	0.02	0.22	9.74
	40-49 years	4.30	2.49	10.02	0.10	0.22	9.74
	50-59 years	15.06	8.52	37.28	0.27	0.17	9.74
	60-69 years	36.67	21.54	74.41	0.77	0.20	9.74
	70-79 years	28.29	19.99	42.69	2.44	1.18	13.71
	≥80 years	32.64	23.56	45.17	8.29	3.48	13.71
IFN-α2	20-39 years	1.84	1.01	5.43	0.02	0.10	8.75
	40-49 years	6.62	3.41	21.70	0.10	0.13	8.75
	50-59 years	29.76	14.14	79.91	0.27	0.08	8.75
	60-69 years	46.10	25.20	87.94	0.77	0.15	8.75
	70-79 years	36.69	24.81	60.02	2.44	0.83	12.52
	≥80 years	40.94	28.85	58.60	8.29	2.54	12.52
IFN-ω	20-39 years	0.80	0.44	1.84	0.02	0.15	5.96
	40-49 years	3.51	1.79	10.05	0.10	0.17	5.96
	50-59 years	11.27	5.85	32.27	0.27	0.14	5.96
	60-69 years	39.29	19.76	84.43	0.77	0.12	5.96
	70-79 years	30.21	19.59	50.32	2.44	0.78	9.64
	≥80 years	40.63	27.03	61.55	8.29	1.97	9.64
IFN-β	20-39 years	0.04	0.01	0.16	0.02	0.77	1.62
	40-49 years	0.72	0.10	5.71	0.10	0.22	1.62
	50-59 years	0.86	0.15	3.21	0.27	0.50	1.62
	60-69 years	4.43	0.66	20.25	0.77	0.28	1.62
	70-79 years	2.21	0.22	9.32	2.44	0.57	0.51
	≥80 years	31.04	2.37	88.06	8.29	0.14	0.51

[a]COVID-19 fatality rate for individuals with auto-Abs

[b]IFR provided by O'Driscoll *et al*. (7)

[c]Prevalence of auto-Abs in the general population

[d]Prevalence of auto-Abs in patients dying from COVID-19.

95% CI: 95% confidence interval.

SI References

1. Bastard P*, et al.* (2021) Autoantibodies neutralizing type I IFNs are present in ~4% of uninfected individuals over 70 years old and account for ~20% of COVID-19 deaths. *Sci Immunol* 6(62).
2. Bastard P*, et al.* (2021) Auto-antibodies to type I IFNs can underlie adverse reactions to yellow fever live attenuated vaccine. *J Exp Med* 218(4).
3. Morabia A, Ten Have T, & Landis JR (1995) Empirical evaluation of the influence of control selection schemes on relative risk estimation: the Welsh nickel workers study. *Occup Environ Med* 52(7):489-493.
4. Heinze G & Schemper M (2002) A solution to the problem of separation in logistic regression. *Stat Med* 21(16):2409-2419.
5. Firth D (1993) Bias reduction of maximum likelihood estimates. *Biometrika* 80(1):27-38.
6. Nagashima K & Sato Y (2017) Information criteria for Firth's penalized partial likelihood approach in Cox regression models. *Stat Med* 36(21):3422-3436.
7. O'Driscoll M*, et al.* (2021) Age-specific mortality and immunity patterns of SARS-CoV-2. *Nature* 590(7844):140-145.
8. Agresti A & Coull BA (1998) Approximate is better than “exact” for interval estimation of binomial proportions. *Am Stat* 52:119-126.

Lists of consortia members

Members of the HGID Lab: Peng Zhang[1], Yoann Seeleuthner[2,3], Estelle Talouarn[2,3], Astrid Marchal[2,3], Daniela Matuozzo[2,3], Aliénor de la Chapelle[2], Jie Chen[1], Maya Chrabieh[2,3], Dana Liu[1], Yelena Nemirowskaya[1], Inés Marín Cruz[2], Marie Materna[2,3], Sophie Pelet[2], Chloé Thibault[2,3], Zhiyong Liu[1]

[1]St. Giles Laboratory of Human Genetics of Infectious Diseases, Rockefeller Branch, The Rockefeller University, New York, NY, USA. [2]Laboratory of Human Genetics of Infectious Diseases, Necker Branch, INSERM U1163, Necker Hospital for Sick Children, Paris, France. [3]University of Paris, Imagine Institute, Paris, France.

Members of the COVID Clinicians: Jorge Abad[1], Giulia Accordino[2], Cristian Achille[3], Sergio Aguilera-Albesa[4], Aina Aguiló-Cucurull[5], Alessandro Aiuti[6], Esra Akyüz Özkan[7], Ilad Alavi Darazam[8], Jonathan Antonio Roblero Albisures[9], Juan C. Aldave[10], Miquel Alfonso Ramos[11], Taj Ali Khan[12], Anna Aliberti[13], Seyed Alireza Nadji[14], Gulsum Alkan[15], Suzan A. AlKhater[16], Jerome Allardet-Servent[17], Luis M. Allende[18], Rebeca Alonso-Arias[19], Mohammed S. Alshahrani[20], Laia Alsina[21], Marie-Alexandra Alyanakian[22], Blanca Amador Borrero[23], Zahir Amoura[24], Arnau Antolí[25], Romain Arrestier[26], Mélodie Aubart[27], Teresa Auguet[28], Iryna Avramenko[29], Gökhan Aytekin[30], Axelle Azot[31], Seiamak Bahram[32], Fanny Bajolle[33], Fausto Baldanti[34], Aurélie Baldolli[35], Maite Ballester[36], Hagit Baris Feldman[37], Benoit Barrou[38], Federica Barzaghi[6], Sabrina Basso[39], Gulsum Iclal Bayhan[40], Alexandre Belot[41], Liliana Bezrodnik[42], Agurtzane Bilbao[43], Geraldine Blanchard-Rohner[44], Ignacio Blanco[45], Adeline Blandinières[46], Daniel Blázquez-Gamero[47], Alexandre Bleibtreu[48], Marketa Bloomfield[49], Mireia Bolivar-Prados[50], Anastasiia Bondarenko[51], Alessandro Borghesi[3], Raphael Borie[52], Elisabeth Botdhlo-Nevers[53], Ahmed A. Bousfiha[54], Aurore Bousquet[55], David Boutolleau[56], Claire Bouvattier[57], Oksana Boyarchuk[58], Juliette Bravais[59], M. Luisa Briones[60], Marie-Eve Brunner[61], Raffaele Bruno[62], Maria Rita P. Bueno[63], Huda Bukhari[64], Jacinta Bustamante[33], Juan José Cáceres Agra[65], Ruggero Capra[66], Raphael Carapito[67], Maria Carrabba[68], Giorgio Casari[6], Carlos Casasnovas[69], Marion Caseris[70], Irene Cassaniti[34], Martin Castelle[71], Francesco Castelli[72], Martín Castillo de Vera[73], Mateus V. Castro[63], Emilie Catherinot[74], Jale Bengi Celik[75], Alessandro Ceschi[76], Martin Chalumeau[77], Bruno Charbit[78], Matthew P. Cheng[79], Pere Clavé[50], Bonaventura Clotet[80], Anna Codina[81], Yves Cohen[82], Roger Colobran[83], Cloé Comarmond[84], Alain Combes[85], Patrizia Comoli[39], Angelo G. Corsico[2], Taner Coşkuner[86], Aleksandar Cvetkovski[87], Cyril Cyrus[88], David Dalmau[89], François Danion[90], David Ross Darley[91], Vincent Das[92], Nicolas Dauby[93], Stéphane Dauger[94], Paul De Munter[95], Loic de Pontual[96], Amin Dehban[97], Geoffroy Delplancq[98], Alexandre Demoule[99], Isabelle Desguerre[100], Antonio Di Sabatino[101], Jean-Luc Diehl[102], Stephanie Dobbelaere[103], Elena Domínguez-Garrido[104], Clément Dubost[105], Olov Ekwall[106], Şefika Elmas Bozdemir[107], Marwa H. Elnagdy[108], Melike Emiroglu[15], Akifumi Endo[109], Emine Hafize Erdeniz[110], Selma Erol Aytekin[111], Maria Pilar Etxart Lasa[112], Romain Euvrard[113], Giovanna Fabio[68], Laurence Faivre[114], Antonin Falck[115], Muriel Fartoukh[116], Morgane Faure[117], Miguel Fernandez Arquero[118], Ricard Ferrer[119], Jose Ferreres[120], Carlos Flores[121], Bruno Francois[122], Victoria Fumadó[123], Kitty S. C. Fung[124], Francesca Fusco[125], Alenka Gagro[126], Blanca Garcia Solis[127], Pascale Gaussem[128], Zeynep Gayretli[129], Juana Gil-Herrera[130], Laurent Gilardin[131], Audrey Giraud Gatineau[132], Mònica Girona-Alarcón[133], Karen Alejandra Cifuentes Godínez[134], Jean-Christophe Goffard[135], Nacho Gonzales[136], Luis I. Gonzalez-Granado[137], Rafaela González-Montelongo[138], Antoine Guerder[139], Belgin Gülhan[140], Victor Daniel Gumucio[141], Leif Gunnar Hanitsch[142], Jan Gunst[143], Marta Gut[144], Jérôme Hadjadj[145], Filomeen Haerynck[146], Rabih Halwani[147], Lennart Hammarström[148], Selda HANCERLI[149], Tetyana Hariyan[150], Nevin Hatipoglu[151], Deniz Heppekcan[152], Elisa Hernandez-Brito[153], Po-ki Ho[154], María Soledad Holanda-Peña[155], Juan P. Horcajada[156], Sami Hraiech[157], Linda Humbert[158], Ivan F. N. Hung[159], Alejandro D. Iglesias[160], Antonio Íñigo-Campos[138], Matthieu Jamme[161], María Jesús Arranz[89], Marie-Thérèse Jimeno[162], Iolanda Jordan[133], Saliha Kanık-Yüksek[163], Yalcin Burak Kara[164], Aydın Karahan[165], Adem Karbuz[166], Kadriye Kart Yasar[167], Ozgur Kasapcopur[168], Kenichi Kashimada[169], Sevgi Keles[111], Yasemin Kendir Demirkol[170], Yasutoshi Kido[171], Can Kizil[172], Ahmet Osman Kılıç[173], Adam Klocperk[174], Maria Daganou[175], Evangelia Koukaki[175], Antonia Koutsoukou[175], Vasiliki Rapti[175], Konstantinos Syrigos[175], Zbigniew J. Król[176], Hatem Ksouri[177], Paul Kuentz[178], Arthur M. C. Kwan[179], Yat Wah M. Kwan[180], Janette S. Y. Kwok[181],

Jean-Christophe Lagier[182], David S. Y. Lam[183], Francesca Conti[184], Andrea Pession[184], Vicky Lampropoulou[310], Fanny Lanternier[185], Yu-Lung Lau[186], Fleur Le Bourgeois[94], Yee-Sin Leo[187], Rafael Leon Lopez[188], Daniel Leung[186], Michael Levin[189], Michael Levy[94], Romain Lévy[33], Zhi Li[78], Daniele Lilleri[34], Edson Jose Adrian Bolanos Lima[190], Agnes Linglart[191], Eduardo López-Collazo[192], José M. Lorenzo-Salazar[138], Céline Louapre[193], Catherine Lubetzki[193], Kwok-Cheung Lung[194], Charles-Edouard Luyt[195], David C. Lye[196], Cinthia Magnone[197], Davood Mansouri[198], Enrico Marchioni[199], Carola Marioli[2], Majid Marjani[200], Laura Marques[201], Jesus Marquez Pereira[202], Andrea Martín-Nalda[203], David Martínez Pueyo[204], Javier Martinez-Picado[205], Iciar Marzana[206], Carmen Mata-Martínez[207], Alexis Mathian[24], Larissa RB Matos[63], Gail V. Matthews[208], Julien Mayaux[209], Raquel McLaughlin-Garcia[210], Philippe Meersseman[211], Jean-Louis Mège[212], Armand Mekontso-Dessap[213], Isabelle Melki[115], Federica Meloni[2], Jean-François Meritet[214], Paolo Merlani[215], Özge Metin Akcan[216], Isabelle Meyts[217], Mehdi Mezidi[218], Isabelle Migeotte[219], Maude Millereux[220], Matthieu Million[221], Tristan Mirault[222], Clotilde Mircher[223], Mehdi Mirsaeidi[224], Yoko Mizoguchi[225], Bhavi P. Modi[226], Francesco Mojoli[13], Elsa Moncomble[227], Abián Montesdeoca Melián[228], Antonio Morales Martinez[229], Francisco Morandeira[230], Pierre-Emmanuel Morange[231], Clémence Mordacq[158], Guillaume Morelle[232], Stéphane J. Mouly[233], Adrián Muñoz-Barrera[138], Cyril Nafati[234], Shintaro Nagashima[235], Yu Nakagama[171], Bénédicte Neven[236], João Farela Neves[237], Lisa F. P. Ng[238], Yuk-Yung Ng[239], Hubert Nielly[105], Yeray Novoa Medina[210], Esmeralda Nuñez Cuadros[240], J. Gonzalo Ocejo-Vinyals[241], Keisuke Okamoto[109], Mehdi Oualha[33], Amani Ouedrani[22], Tayfun Özçelik[242], Aslinur Ozkaya-Parlakay[140], Michele Pagani[13], Qiang Pan-Hammarström[148], Maria Papadaki[310], Christophe Parizot[209], Philippe Parola[244], Tiffany Pascreau[245], Stéphane Paul[246], Estela Paz-Artal[247], Sigifredo Pedraza-Sánchez[248], José Luis Gálvez-Romero[248], Nancy Carolina González Pellecer[134], Silvia Pellegrini[249], Rebeca Pérez de Diego[127], Xosé Luis Pérez-Fernández[141], Aurélien Philippe[250], Quentin Philippot[116], Adrien Picod[251], Marc Pineton de Chambrun[85], Antonio Piralla[34], Laura Planas-Serra[252], Dominique Ploin[253], Julien Poissy[254], Géraldine Poncelet[70], Garyphallia Poulakou[175], Marie S. Pouletty[255], Persia Pourshahnazari[256], Jia Li Qiu-Chen[257], Paul Quentric[209], Thomas Rambaud[258], Didier Raoult[212], Violette Raoult[259], Anne-Sophie Rebillat[223], Claire Redin[260], Léa Resmini[261], Pilar Ricart[262], Jean-Christophe Richard[263], Raúl Rigo-Bonnin[264], Nadia rivet[46], Jacques G. Rivière[265], Gemma Rocamora-Blanch[25], Mathieu P. Rodero[266], Carlos Rodrigo[267], Luis Antonio Rodriguez[190], Carlos Rodriguez-Gallego[268], Agustí Rodriguez-Palmero[269], Carolina Soledad Romero[270], Anya Rothenbuhler[271], Damien Roux[272], Nikoletta Rovina[175], Flore Rozenberg[273], Yvon Ruch[90], Montse Ruiz[274], Maria Yolanda Ruiz del Prado[275], Juan Carlos Ruiz-Rodriguez[119], Joan Sabater-Riera[141], Kai Saks[276], Maria Salagianni[310], Oliver Sanchez[277], Adrián Sánchez-Montalvá[278], Silvia Sánchez-Ramón[279], Laire Schidlowski[280], Agatha Schluter[252], Julien Schmidt[281], Matthieu Schmidt[282], Catharina Schuetz[283], Cyril E. Schweitzer[284], Francesco Scolari[285], Anna Sediva[286], Luis Seijo[287], Analia Gisela Seminario[42], Damien Sene[23], Piseth Seng[221], Sevtap Senoglu[167], Mikko Seppänen[288], Alex Serra Llovich[289], Mohammad Shahrooei[97], Anna Shcherbina[290], Virginie Siguret[291], Eleni Siouti[310], David M. Smadja[293], Nikaia Smith[78], Ali Sobh[294], Xavier Solanich[25], Jordi Solé-Violán[295], Catherine Soler[296], Pere Soler-Palacín[297], Betül Sözeri[86], Giulia Maria Stella[2], Yuriy Stepanovskiy[298], Annabelle Stoclin[299], Fabio Taccone[219], Yacine Tandjaoui-Lambiotte[300], Jean-Luc Taupin[301], Simon J. Tavernier[302], Loreto Vidaur[112], Benjamin Terrier[303], Guillaume Thiery[304], Christian Thorball[260], Karolina Thorn[305], Caroline Thumerelle[158], Imran Tipu[306], Martin Tolstrup[307], Gabriele Tomasoni[308], Julie Toubiana[77], Josep Trenado Alvarez[309], Sophie Trouillet-Assant[311], Jesús Troya[312], Owen T. Y. Tsang[313], Liina Tserel[314], Eugene Y. K. Tso[315], Alessandra Tucci[316], Şadiye Kübra Tüter Öz[15], Matilde Valeria Ursini[125], Takanori Utsumi[225], Yurdagul Uzunhan[317], Pierre Vabres[318], Juan Valencia-Ramos[319], Ana Maria Van Den Rym[127], Isabelle Vandernoot[320], Valentina Velez-Santamaria[321], Silvia Patricia Zuniga Veliz[134], Mateus C. Vidigal[322], Sébastien Viel[253], Cédric Villain[323], Marie E. Vilaire-Meunier[223], Judit Villar-García[324], Audrey Vincent[57], Guillaume Vogt[325], Guillaume Voiriot[326], Alla Volokha[327], Fanny Vuotto[158], Els Wauters[328], Joost Wauters[329], Alan K. L. Wu[330], Tak-Chiu Wu[331], Aysun Yahşi[332], Osman Yesilbas[333], Mehmet Yildiz[168], Barnaby E. Young[187], Ufuk Yükselmiş[334], Mayana Zatz[63], Stefano Ghirardello[3], Valentina Zuccaro[62], Ana de Andrés[335], Jens Van Praet[336], Bart N. Lambrecht[337], Eva Van Braeckel[337], Cédric Bosteels[337], Levi Hoste[338], Eric Hoste[339], Fré Bauters[337], Jozefien De Clercq[337], Cathérine Heijmans[340], Hans Slabbynck[341], Leslie Naesens[342], Benoit Florkin[343], Cécile Boulanger[344], Dimitri Vanderlinden[345]

[1]Germans Trias i Pujol University Hospital and Research Institute, Badalona, Barcelona, Spain. [2]Respiratory Diseases Division, IRCCS Policlinico San Matteo Foundation, University of Pavia, Pavia, Italy. [3]Neonatal Intensive Care Unit, Fondazione IRCCS Policlinico San Matteo, Pavia, Italy. [4]Navarra Health Service Hospital, Pamplona, Spain. [5]Jeffrey Modell Diagnostic and Research Center for Primary Immunodeficiencies, Barcelona, Catalonia, Spain; Immunology Division, Genetics Department, Vall d'Hebron University Hospital (HUVH), Vall d'Hebron Research Institute (VHIR), Vall d'Hebron Barcelona Hospital Campus, Universitat Autònoma de Barcelona (UAB), Barcelona, Catalonia, Spain. [6]Immunohematology Unit, San Raffaele Hospital, Milan, Italy. [7]Ondokuz Mayıs University Medical Faculty Pediatrics, Samsun, Turkey. [8]Department of Infectious Diseases, Loghman Hakim Hospital, Shahid Beheshti University of Medical Sciences, Tehran, Iran. [9]Hospital Regional de Huehuetenango, "Dr. Jorge Vides de Molina," Guatemala. [10]Hospital Nacional Edgardo Rebagliati Martins, Lima, Peru. [11]Parc Sanitari Sant Joan de Déu, Sant Boi de Llobregat Spain. [12]Khyber Medical University, Khyber Pakhtunkhwa, Pakistan. [13]Anesthesia and Intensive Care, Rianimazione I, Fondazione IRCCS Policlinico San Matteo, Pavia, Italy. [14]Virology Research Center, National Research Institute of Tuberculosis and lung diseases, Shahid Beheshti University of Medical Sciences, Tehran, Iran. [15]Department of Pediatrics, Division of Pediatric Infectious Diseases, Selcuk University Faculty of Medicine, Konya, Turkey. [16]College of Medicine, Imam Abdulrahman Bin Faisal University, Dammam, Saudi Arabia; Department of Pediatrics, King Fahad Hospital of the University, Al-Khobar, Saudi Arabia. [17]Intensive care unit, Hôpital Européen, Marseille, France. [18]Immunology Department, Hospital 12 de Octubre, Research Institute imas12, Complutense University, Madrid, Spain. [19]Immunology Department, Asturias Central University Hospital, Biosanitary Research Institute of the Principality of Asturias (ISPA), Oviedo, Spain. [20]Emergency and Critical Care Medicine Departments, College of Medicine, Imam AbdulRahman Ben Faisal University, Dammam, Saudi Arabia. [21]Clinical Immunology and Primary Immunodeficiencias Unit, Hospital Sant Joan de Déu, Institut de Recerca Sant Joan de Déu, Barcelona; Universitat de Barcelona, Barcelona, Spain. [22]Department of Biological Immunology, Necker Hospital for Sick Children, APHP and INEM, Paris, France. [23]Internal medicine department, Hôpital Lariboisière, APHP; Université de Paris, Paris, France. [24]Internal medicine department, Pitié-Salpétrière Hospital, Paris, France. [25]Department of Internal Medicine, Hospital Universitari de Bellvitge, IDIBELL, Barcelona, Spain. [26]Service de Médecine Intensive Réanimation, Hôpitaux Universitaires Henri Mondor, Assistance Publique - Hôpitaux de Paris (AP-HP); Groupe de Recherche Clinique CARMAS, Faculté de Santé de Créteil, Université Paris Est Créteil, Créteil, France. [27]INSERM U1163, University of Paris, Imagine Institute, Paris, France & Pediatric Neurology Department, Necker-Enfants malades Hospital, APHP, Paris, France. [28]Hospital U. de Tarragona Joan XXIII. Universitat Rovira i Virgili (URV). IISPV, Tarragona, Spain. [29]Department of Propedeutics of Pediatrics and Medical Genetics, Danylo Halytsky Lviv National Medical University, Lviv, Ukraine. [30]Department of Immunology and Allergy, Konya City Hospital, Konya, Turkey. [31]Private practice, Paris, France. [32]INSERM U1109, University of Strasbourg, Strasbourg, France. [33]Necker Hospital for Sick Children, AP-HP, Paris, France. [34]Molecular Virology Unit, Microbiology and Virology Department, Fondazione IRCCS Policlinico San Matteo and Department of Clinical, Surgical, Diagnostic and Pediatric Sciences, University of Pavia, Pavia, Italy. [35]Department of Infectious Diseases, CHU de Caen, Caen, France. [36]Consorcio Hospital General Universitario, Valencia, Spain. [37]The Genetics Institute, Tel Aviv Sourasky Medical Center and Sackler Faculty of Medicine, Tel Aviv University, Tel Aviv, Israel. [38]Department Urology, Nephrology, Transplantation, APHP-SU, Sorbonne Université, INSERM U 1082, Paris, France. [40]Yildirim Beyazit University, Faculty of Medicine, Ankara City Hospital, Children's Hospital, Ankara, Turkey. [41]University of Lyon, CIRI, INSERM U1111, National referee centre RAISE, Pediatric Rheumatology, HFME, Hospices Civils de Lyon, Lyon, France. [42]Center for Clinical Immunology, CABA, Buenos Aires, Argentina. [43]Cruces University Hospital, Bizkaia, Spain. [44]Paediatric Immunology and Vaccinology Unit, Geneva University Hospitals and Faculty of Medicine, Geneva, Switzerland. [45]University Hospital and Research Institute "Germans Trias i Pujol," Badalona, Spain. [46]Hematology, Georges Pompidou Hospital, APHP, Paris, France. [47]Pediatric Infectious Diseases Unit, Instituto de Investigación Hospital 12 de Octubre (imas12), Hospital Universitario 12 de Octubre, Universidad Complutense, Madrid, Spain. [48]Infectious disease Unit, Pitié-Salpêtrière Hospital, AP-AP, Paris, France. [49]Department of Pediatrics, 1st Faculty of Medicine, Charles University and Thomayer University Hospital, Prague, Czech Republic; Department of Immunology, Motol University Hospital, 2nd

Faculty of Medicine, Charles University, Prague, Czech Republic. [50]Centro de Investigación Biomédica en Red de Enfermedades Hepàticas y Digestivas (Ciberehd). Hospital de Mataró, Consorci Sanitari del Maresme, Mataró, Spain. [51]Shupyk National Healthcare University of Ukraine, Kyiv, Ukraine. [52]Service de Pneumologie, Hopital Bichat, APHP, Paris, France. [53]Department of infectious diseases, CIC1408, GIMAP CIRI INSERM U1111, University Hospital of Saint-Étienne, Saint-Étienne, France. [54]Clinical immunology unit, pediatric infectious disease departement, Faculty of Medicine and Pharmacy, Averroes University Hospital. LICIA Laboratoire d'immunologie clinique, d'inflammation et d'allergie, Hassann Ii University., Casablanca, Morocco. [55]Bégin Military Hospital, Saint-Mandé, France. [56]Sorbonne Université, INSERM, Institut Pierre Louis d'Epidémiologie et de Santé Publique (iPLESP), AP-HP, Hôpital Pitié Salpêtrière, Service de Virologie, Paris, France. [57]Endocrinology unit, APHP Hôpitaux Universitaires Paris-Sud, Le Kremlin-Bicêtre, France. [58]Department of Children's Diseases and Pediatric Surgery, I. Horbachevsky Ternopil National Medical University, Ternopil, Ukraine. [59]Pneumology Unit, Tenon Hospital, AP-HP, Paris, France. [60]Department of Respiratory Diseases, Hospital Clínico y Universitario de Valencia, Valencia, Spain. [61]Intensive care unit, Réseau Hospitalier Neuchâtelois, Neuchâtel, Switzerland. [62]Infectious Diseases Unit, Fondazione IRCCS Policlinico San Matteo, Pavia, Italy. [63]Human Genome and Stem Cell Research Center, University of São Paulo, São Paulo, Brazil. [64]Department of Internal Medicine, College of Medicine, Imam Abdulrahman Bin Faisal University, Dammam, Saudi Arabia. [65]Hospital Insular, Las Palmas de Gran Canaria, Spain. [66]MS Center, Spedali Civili, Brescia, Italy. [67]Laboratoire d'ImmunoRhumatologie Moléculaire, plateforme GENOMAX, INSERM UMR_S 1109, Faculté de Médecine, ITI TRANSPLANTEX NG, Université de Strasbourg, Strasbourg, France. [68]Fondazione IRCCS Ca' Granda Ospedale Maggiore Policlinico, Milan, Italy. [69]Neuromuscular Unit, Neurology Department, Hospital Universitari de Bellvitge, IDIBELL and CIBERER, Barcelona, Spain. [70]Hopital Robert Debré, Paris, France. [71]Pediatric Immuno-Hematology Unit, Necker Enfants Malades Hospital, AP-HP, Paris, France. [72]Department of Infectious and Tropical Diseases, University of Brescia, ASST Spedali Civili di Brescia, Brescia, Italy. [73]Doctoral Health Care Center, Canarian Health System, Las Palmas de Gran Canaria, Spain. [74]Hôpital Foch, Suresnes, France. [75]Selcuk University Faculty of Medicine, Department of Anesthesiology and Reanimation, Intensive Care Medicine Unit, Konya, Turkey. [76]Division of Clinical Pharmacology and Toxicology, Institute of Pharmacological Sciences of Southern Switzerland, Ente Ospedaliero Cantonale and Faculty of Biomedical Sciences, Università della Svizzera italiana, Lugano, Switzerland. [77]Necker Hospital for Sick Children, Paris University, AP-HP, Paris, France. [78]Pasteur Institute, Paris, France. [79]McGill University Health Centre, Montreal, Canada. [80]University Hospital and Research Institute Germans Trias i Pujol, IrsiCaixa AIDS Research Institute, UVic-UCC, Badalona, Spain. [81]Clinical Biochemistry, Pathology, Paediatric Neurology and Molecular Medicine Departments and Biobank, Institut de Recerca Sant Joan de Déu and CIBERER-ISCIII, Esplugues, Spain. [82]AP-HP, Avicenne Hospital, Intensive Care Unit, Bobigny, France; University Sorbonne Paris Nord, Bobigny, France; INSERM, U942, F-75010, Paris, France. [83]Hospital Universitari Vall d'Hebron, Barcelona, Spain. [84]Pitié-Salpêtrière Hospital, Paris, France. [85]Service de médecine Intensive Réanimation, Groupe Hospitalier Pitié-Salpêtrière, Sorbonne Université, France. [86]Umraniye Training and Research Hospital, Istanbul, Turkey. [87]Faculty of Medical Sciences at University "Goce Delcev", Shtip, North Macedonia. [88]Department of Biochemistry, College of Medicine, Imam Abdulrahman Bin Faisal University, Dammam, Saudi Arabia. [89]Fundació Docencia i Recerca Mutua Terrassa, Barcelona, Spain. [90]Maladies Infectieuses et Tropicales, Nouvel Hôpital Civil, CHU Strasbourg, Strasbourg, France. [91]UNSW Medicine, St Vincent's Clinical School; Department of Thoracic Medicine, St Vincent's Hospital Darlinghurst, Sydney, Australia. [92]Intensive Care Unit, Montreuil Hospital, Montreuil, France. [93]CHU Saint-Pierre, Université Libre de Bruxelles (ULB), Brussels, Belgium. [94]Pediatric Intensive Care Unit, Robert-Debré University Hospital, APHP, Paris, France. [95]General Internal Medicine, University Hospitals Leuven, Belgium. [96]Hôpital Jean Verdier, APHP, Bondy, France. [97]Specialized Immunology Laboratory of Dr. Shahrooei, Sina Medical Complex, Ahvaz, Iran. [98]Centre de génétique humaine, CHU Besançon, Besançon, France. [99]Sorbonne Université Médecine and APHP Sorbonne Université site Pitié-Salpêtrière, Paris, France. [100]Pediatric Neurology Department, Necker-Enfants Malades Hospital, APHP, Paris, France. [101]Department of Internal Medicine, Fondazione IRCCS Policlinico San Matteo, University of Pavia, Pavia, Italy. [102]Intensive Care Unit, Georges Pompidou Hospital, APHP, Paris, France. [103]Department of Pneumology, AZ Delta, Roeselare, Belgium. [104]Molecular Diagnostic Unit, Fundación Rioja Salud,

Logroño, La Rioja, Spain. [105]Bégin Military Hospital, Saint-Mandé, France. [106]Department of Pediatrics, Institute of Clinical Sciences, Sahlgrenska Academy, University of Gothenburg, Gothenburg, Sweden; Department of Rheumatology and Inflammation Research, Institute of Medicine, Sahlgrenska Academy, University of Gothenburg, Gothenburg, Sweden. [107]Bursa City Hospital, Bursa, Turkey. [108]Department of Medical Biochemistry and Molecular Biology, Faculty of Medicine, Mansoura University, Mansoura, Egypt. [109]Tokyo Medical and Dental University, Tokyo, Japan. [110]Ondokuz Mayıs University Faculty of Medicine, Samsun, Turkey. [111]Necmettin Erbakan University, Meram Medical Faculty, Division of Pediatric Allergy and Immunology, Konya, Turkey. [112]Intensive Care Medicine, Donostia University Hospital, Biodonostia Institute of Donostia, CIBER Enfermedades Respiratorias ISCIII, Donostia, Spain. [113]Internal Medicine, University Hospital Edouard Herriot, Hospices Civils de Lyon, Lyon, France. [114]Centre de Génétique, CHU Dijon, Dijon, France. [115]Robert Debré Hospital, Paris, France. [116]APHP Tenon Hospital, Paris, France. [117]Sorbonne Universités, UPMC University of Paris, Paris, France. [118]Department of Clinical Immunology, Hospital Clínico San Carlos, Madrid, Spain. [119]Intensive Care Department, HUVH, Vall d'Hebron Barcelona Hospital Campus, Barcelona, Catalonia, Spain; Shock, Organ Dysfunction and Resuscitation Research Group, Vall d'Hebron Research Institute (VHIR), Vall d'Hebron Barcelona Hospital Campus, Barcelona, Catalonia, Spain. [120]Intensive Care Unit, Hospital Clínico y Universitario de Valencia, Valencia, Spain. [121]Genomics Division, Instituto Tecnológico y de Energías Renovables (ITER), Santa Cruz de Tenerife, Spain; CIBER de Enfermedades Respiratorias, Instituto de Salud Carlos III, Madrid, Spain; Research Unit, Hospital Universitario N.S. de Candelaria, Santa Cruz de Tenerife, Spain; Instituto de Tecnologías Biomédicas (ITB), Universidad de La Laguna, San Cristóbal de La Laguna, Spain, Santa Cruz de Tenerife, Spain. [122]CHU Limoges and INSERM CIC 1435 and UMR 1092, Limoges, France. [123]Infectious Diseases Unit, Department of Pediatrics, Hospital Sant Joan de Déu, Barcelona, Spain; Institut de Recerca Sant Joan de Déu, Spain; Universitat de Barcelona (UB), Barcelona, Spain. [124]Department of Pathology, United Christian Hospital, Hong Kong. [125]Institute of Genetics and Biophysics "Adriano Buzzati-Traverso," IGB-CNR, Naples, Italy. [126]Department of Pediatrics, Children's Hospital Zagreb, University of Zagreb School of Medicine, Zagreb, Josip Juraj Strossmayer University of Osijek, Medical Faculty Osijek, Osijek, Croatia. [127]Laboratory of Immunogenetics of Human Diseases, IdiPAZ Institute for Health Research, La Paz Hospital, Madrid, Spain. [128]Hematology, APHP, Hopital Européen Georges Pompidou and INSERM UMR-S1140, Paris, France. [129]Faculty of Medicine, Department of Pediatrics, Division of Pediatric Infectious Diseases, Karadeniz Technical University, Trabzon, Turkey. [130]Division of Immunology, Hospital General Universitario and Instituto de Investigación Sanitaria "Gregorio Marañón", Madrid, Spain. [131]Bégin military Hospital, Saint-Mandé, France. [132]Aix-Marseille University, IRD, AP-HM, SSA, VITROME, IHU Méditerranée Infection, Marseille, France, French Armed Forces Center for Epidemiology and Public Health (CESPA), Marseille, France. [133]Pediatric Intensive Care Unit, Hospital Sant Joan de Déu, Barcelona, Spain. [134]Gestión Integral en Salud, San José Pinula, Guatemala. [135]Department of Internal Medicine, Hôpital Erasme, Université Libre de Bruxelles, Brussels, Belgium. [136]Immunodeficiencies Unit, Research Institute Hospital, Madrid, Spain. [137]Primary Immunodeficiencies Unit, Pediatrics, University Hospital 12 octubre, Madrid, Spain; School of Medicine Complutense University of Madrid, Madrid, Spain. [138]Genomics Division, Instituto Tecnológico y de Energías Renovables (ITER), Santa Cruz de Tenerife, Spain. [139]Assistance Publique Hôpitaux de Paris, Paris, France. [140]Ankara City Hospital, Ankara, Turkey. [141]Department of Intensive Care, Hospital Universitari de Bellvitge, IDIBELL, Barcelona, Spain. [142]Immunodeficiency Outpatient Clinic, Institute for Medical Immunology, FOCIS Center of Excellence, Charité Universitätsmedizin Berlin, Germany. [143]Surgical Intensive Care Unit, University Hospitals Leuven, Leuven, Belgium. [144]CNAG-CRG, Barcelona Institute of Science and Technology, Barcelona, Spain. [145]Department of Internal Medicine, National Reference Center for Rare Systemic Autoimmune Diseases, AP-HP, APHP-CUP, Hôpital Cochin, Paris, France. [146]Department of Paediatric Immunology and Pulmonology, Center for Primary Immunodeficiency Ghent, Jeffrey Modell Diagnosis and Research Center, PID Research Lab, Ghent University Hospital, Ghent, Belgium. [147]Sharjah Institute of Medical Research, College of Medicine, University of Sharjah, Sharjah, UAE, Sharjah, UAE. [148]Department of Biosciences and Nutrition, SE14183, Huddinge, Karolinska Institutet, Stockholm, Sweden. [149]Department of Pediatrics (Infectious Diseases), Istanbul Faculty of Medicine, Istanbul University, Istanbul, Turkey. [150]I. Horbachevsky Ternopil National Medical University, Ternopil, Ukraine. [151]Pediatric Infectious

Diseases Unit, Bakirkoy Dr. Sadi Konuk Training and Research Hospital, University of Health Sciences, Istanbul, Turkey. [152]Health Sciences University, Darıca Farabi Education and Research Hospital, Kocaeli, Turkey. [153]Department of Immunology, Hospital Universitario de Gran Canaria Dr. Negrín, Canarian Health System, Las Palmas de Gran Canaria, Spain. [154]Department of Paediatrics, Queen Elizabeth Hospital, Hong Kong. [155]IntensivenCare Unit, Marqués de Valdecilla Hospital, Santander, Spain. [156]Hospital del Mar, Institut Hospital del Mar d'Investigacions Mèdiques (IMIM), UAB, UPF, Barcelona. [157]Intensive care unit, APHM, Marseille, France. [158] CHU Lille, unité de pneumologie et allergologie pédiatriques, Lille, France. [159]Department of Medicine, The University of Hong Kong, Hong Kong. [160]Department of Pediatrics, Columbia University, New York, NY, USA. [161]Centre hospitalier intercommunal Poissy Saint Germain en Laye, Poissy, France. [162]IHU Méditerranée Infection, Service de l'Information Médicale, Hôpital de la Timone, Marseille, France. [163]Health Science University Ankara City Hospital, Ankara, Turkey. [164]School of Medicine, General Surgery Department Fevzi Çakmak Mah, Marmara University, Istanbul, Turkey. [165]Mersin City Education and Research Hospital, Mersin, Turkey. [166]Division of Pediatric Infectious Diseases, Prof. Dr. Cemil Tascıoglu City Hospital, Istanbul, Turkey. [167]Departments of Infectious Diseases and Clinical Microbiology, Bakirkoy Dr. Sadi Konuk Training and Research Hospital, University of Health Sciences, Istanbul, Turkey. [168]Department of Pediatric Rheumatology, Istanbul University-Cerrahpasa, Istanbul, Turkey. [169]Department of Pediatrics, Tokyo Medical and Dental University, Tokyo, Japan. [170]Health Sciences University, Umraniye Education and Research Hospital, Istanbul, Turkey. [171]Department of Parasitology and Research Center for Infectious Disease Sciences, Graduate School of Medicine, Osaka City University, Osaka, Japan. [172]Pediatric Infectious Diseases Unit of Osman Gazi University Medical School in Eskişehir, Turkey. [173]Meram Medical Faculty, Necmettin Erbakan University, Konya, Turkey. [174]Department of Immunology, 2nd Faculty of Medicine, Charles University and University Hospital in Motol, Prague, Czech Republic. [175] National and Kapodistrian University of Athens, Medical School and "Sotiria" General Hospital of Chest Diseases, Athens, Greece. [176]Central Clinical Hospital of the Ministry of Interior and Administration, Warsaw, Poland. [177]Clinique des soins intensifs, HFR Fribourg, Fribourg, Switzerland. [178]Oncobiologie Génétique Bioinformatique, PC Bio, CHU Besançon, Besançon, France. [179]Department of Intensive Care, Tuen Mun Hospital, Hong Kong. [180]Paediatric Infectious Disease Unit, Hospital Authority Infectious Disease Center, Princess Margaret Hospital, Hong Kong (Special Administrative Region), China. [181]Department of Pathology, Queen Mary Hospital, Hong Kong. [182]Aix-Marseille Univ, IRD, MEPHI, IHU Méditerranée Infection, Marseille, France. [183]Department of Paediatrics, Tuen Mun Hospital, Hong Kong. [184]Pediatric Unit, IRCCS Azienda Ospedaliero-Universitaria di Bologna, Bologna, Italy.[185]Necker hospital, Paris, France. [186]Department of Paediatrics and Adolescent Medicine, The University of Hong Kong, Hong Kong, China. [187]National Centre for Infectious Diseases, Singapore. [188]Hospital Universitario Reina Sofía, Cordoba, Spain. [189]Imperial College, London, England. [190]Hospital General San Juan de Dios, Ciudad de Guatemala, Guatemala. [191]Endocrinology and Diabetes for Children, AP-HP, Bicêtre Paris-Saclay Hospital, Le Kremlin-Bicêtre, France. [192]Innate Immunity group, IdiPAZ Institute for Health Research, La Paz Hospital, Madrid, Spain. [193]Neurology unit, APHP Pitié-Salpêtrière Hospital, Paris University, Paris, France. [194]Department of Medicine, Pamela Youde Nethersole Eastern Hospital, Hong Kong. [195]Intensive care unit, APHP Pitié-Salpêtrière Hospital, Paris University, Paris, France. [196]National Centre for Infectious Diseases; Tan Tock Seng Hospital; Yong Loo Lin School of Medicine; Lee Kong Chian School of Medicine, Singapore. [197]Hospital de Niños Dr. Ricardo Gutierrez, Buenos Aires, Argentina. [198]Department of Clinical Immunology and Infectious Diseases, National Research Institute of Tuberculosis and Lung Diseases, Shahid Beheshti University of Medical Sciences, Tehran, Iran. [199]Neurooncology and Neuroinflammation Unit, IRCCS Mondino Foundation, Pavia, Italy. [200]Clinical Tuberculosis and Epidemiology Research Center, National Research Institute of Tuberculosis and Lung Diseases (NRITLD), Shahid Beheshti University of Medical Sciences, Tehran, Iran. [201]Coordenadora da Unidade de Infeciologia e Imunodeficiências do Serviço de Pediatria, Centro Materno-Infantil do Norte, Porto, Portugal. [202]Hospital Sant Joan de Déu and University of Barcelona, Barcelona, Spain. [203]Pediatric Infectious Diseases and Immunodeficiencies Unit, Hospital Universitari Vall d'Hebron, VHIR, Vall d'Hebron Barcelona Hospital Campus, Universitat Autònoma de Barcelona (UAB), Barcelona, Catalonia, Spain. [204]Hospital Universitari Mutua de Terrassa, Universitat de Barcelona, Barcelona, Spain. [205]IrsiCaixa AIDS Research Institute, ICREA, UVic-UCC, Research Institute Germans Trias i Pujol, Badalona, Spain.

[206]Department of Laboratory, Cruces University Hospital, Barakaldo, Bizkaia, Spain, Bizkaia, Spain. [207]Intensive Care Unit, Hospital General Universitario Gregorio Marañón, Madrid, Spain. [208]Department of Infectious Diseases, Kirby Institute, UNSW Sydney, Sydney, NSW 2052, Australia; St Vincent's Hospital Sydney, Darlinghurst, NSW, Australia. [209]APHP Pitié-Salpêtrière Hospital, Paris, France. [210]Department of Pediatrics, Complejo Hospitalario Universitario Insular-Materno Infantil, Canarian Health System, Las Palmas de Gran Canaria, Spain. [211]Medical Intensive Care Unit, University Hospitals Leuven, Leuven, Belgium. [212]Aix-Marseille University, APHM, Marseille, France. [213]Service de Médecine Intensive Réanimation, Hôpitaux Universitaires Henri Mondor, AP-HP. Groupe de Recherche Clinique CARMAS, Faculté de Santé de Créteil, Université Paris Est Créteil, France. [214]APHP Cohin Hospital, Paris, France. [215]Department of Critical Care Medicine, Ente Ospedaliero Cantonale, Bellinzona, Switzerland. [216]Necmettin Erbakan University, Meram Medical Faculty, Division of Pediatric Infectious Diseases, Konya, Turkey. [217]Department of Pediatrics, University Hospitals Leuven; KU Leuven, Department of Microbiology, Immunology and Transplantation; Laboratory for Inborn Errors of Immunity, KU Leuven, Leuven, Belgium. [218]Hospices Civils de Lyon, Hôpital de la Croix-Rousse, Lyon, France. [219]Center of Human Genetics, Hôpital Erasme, Brussels, Belgium. [220]Centre hospitalier de Gonesse, Gonesse, France. [221]Aix-Marseille University, IRD, AP-HM, MEPHI, IHU Méditerranée Infection, Marseille, France. [222]Vascular Medicine, Georges Pompidou Hospital, APHP, Paris, France. [223]Institut Jérôme Lejeune, Paris, France. [224]Division of Pulmonary and Critical Care, College of Medicine-Jacksonville, University of Florida, Jacksonville, FL, USA. [225]Department of Pediatrics, Hiroshima University Graduate School of Biomedical and Health Sciences, Hiroshima, Japan. [226]BC Children's Hospital Research Institute, University of British Columbia, Vancouver, Canada. [227]Médecine Intensive Réanimation, Hôpitaux Universitaires Henri Mondor, AP-HP, Créteil, France. [228]Guanarteme Health Care Center, Canarian Health System, Las Palmas de Gran Canaria, Spain. [229]Regional University Hospital of Malaga, Malaga, Spain. [230]Department of Immunology, Hospital Universitari de Bellvitge, IDIBELL, Barcelona, Spain. [231]Aix-Marseille University, INSERM, INRAE, C2VN, Marseille, France. [232]Department of General Paediatrics, Hôpital Bicêtre, AP-HP, University of Paris-Saclay, Le Kremlin-Bicêtre, France. [233]INSERM U1144, Université de Paris, DMU INVICTUS, APHP-Nord, Département de Médecine Interne, Lariboisière Hospital, Paris, France. [234]CHU de La Timone, Marseille, France. [235]Department of Epidemiology, Infectious Disease Control and Prevention, Graduate School of Biomedical and Health Sciences, Hiroshima University, Hiroshima, Japan. [236]Pediatric Immunology and rhumatology Department, Necker Hospital, AP-HP, Paris, France. [237]Centro Hospitalar Universitário de Lisboa Central, Lisbon, Portugal. [238]Infectious Diseases Horizontal Technlogy Centre, A*STAR; Singapore Immunology Network, A*STAR, Singapore. [239]Department of Medicine and Geriatrics, Tuen Mun Hospital, Hong Kong. [240]Regional Universitary Hospital of Malaga, Málaga, Spain. [241]Department of Immunology, Hospital Universitario Marqués de Valdecilla, Santander, Spain. [242]Bilkent University, Department of Molecular Biology and Genetics, Ankara, Turkey. [244]IHU Méditerranée Infection, Aix-Marseille Univ, IRD, AP-HM, SSA, VITROME, IHU Méditerranée Infection, Marseille, France. [245]L'Hôpital Foch, Suresnes, France. [246]Department of Immunology, CIC1408, GIMAP CIRI INSERM U1111, University Hospital of Saint-Étienne, Saint-Étienne, France. [247]Department of Immunology, Hospital Universitario 12 de Octubre, Instituto de Investigación Sanitaria Hospital 12 de Octubre (imas12), Madrid, Spain. [248]Instituto Nacional de Ciencias Médicas y Nutrición Salvador Zubirán, Mexico. [249]Diabetes Research Institute, IRCCS San Raffaele Hospital, Milan, Italy. [250]APHP Hôpitaux Universitaires Paris-Sud, Le Kremlin-Bicêtre, France. [251]AP-HP, Avicenne Hospital, Intensive Care Unit, Bobigny, France; INSERM UMR-S 942, Cardiovascular Markers in Stress Conditions (MASCOT), University of Paris, Paris, France. [252]Neurometabolic Diseases Laboratory, IDIBELL-Hospital Duran i Reynals, Barcelona; CIBERER U759, ISCiii Madrid, Spain. [253]Hospices Civils de Lyon, Lyon, France. [254]Univ. Lille, INSERM U1285, CHU Lille, Pôle de médecine intensive-réanimation, CNRS, UMR 8576-Unité de Glycobiologie Structurale et Fonctionnelle, Lille, France. [255]Department of General pediatrics, Robert Debre Hospital, Paris, France. [256]University of British Columbia, Vancouver, Canada. [257]Jeffrey Modell Diagnostic and Research Center for Primary Immunodeficiencies, Barcelona, Catalonia, Spain, Diagnostic Immunology Research Group, VHIR, HUVH, Vall d'Hebron Barcelona Hospital Campus, Barcelona, Catalonia, Spain. [258]AP-HP, Avicenne Hospital, Intensive Care Unit, Bobigny, France; University Sorbonne Paris Nord, Bobigny, France. [259]Centre Hospitalier de Saint-Denis, Saint-Denis, France. [260]Precision Medicine Unit, Lausanne University Hospital and University of Lausanne,

Lausanne, Switzerland. [261]Paris Cardiovascular Center, PARCC, INSERM, Université de Paris, Paris, France. [262]Germans Trias i Pujol Hospital, Badalona, Spain. [263]Medical intensive care unit. Hopital de la Croix-Rousse. Hospices Civils de Lyon, Lyon, France. [264]Department of Clinical Laboratory, Hospital Universitari de Bellvitge, IDIBELL, Barcelona, Spain. [265]Pediatric Infectious Diseases and Immunodeficiencies Unit, Hospital Universitari Vall d'Hebron, VHIR, Vall d'Hebron Barcelona Hospital Campus, Barcelona, Spain. [266]Université de Paris, CNRS UMR-8601; Team Chemistry & Biology, Modeling & Immunology for Therapy, CBMIT, Paris, France. [267]Germans Trias i Pujol University Hospital and Research Institute, Badalona, Badalona, Spain. [268]Department of Immunology, University Hospital of Gran Canaria Dr. Negrín, Canarian Health System, Las Palmas de Gran Canaria, Spain; Department of Clinical Sciences, University Fernando Pessoa Canarias, Las Palmas de Gran Canaria, Spain. [269]Neurometabolic Diseases Laboratory, Bellvitge Biomedical Research Institute (IDIBELL), 08908 L'Hospitalet de Llobregat; University Hospital Germans Trias i Pujol, Badalona, Barcelona, Catalonia, Spain. [270]Consorcio Hospital General Universitario, Valencia, Spain. [271]APHP Hôpitaux Universitaires Paris-Sud, Paris, France. [272]Intensive Care Unit, Louis-Mourier Hospital, Colombes, France. [273]Virology unit, Université de Paris, Cohin Hospital, APHP, Paris, France. [274]Neurometabolic Diseases Laboratory and CIBERER U759, Barcelona, Spain. [275]Hospital San Pedro, Logroño, Spain. [276]University of Tartu, Institute of Biomedicine and Translational Medicine, Tartu, Estonia. [277]Respiratory medicine, Georges Pompidou Hospital, APHP, Paris, France. [278]Infectious Diseases Department, International Health Program of the Catalan Insitute of Health (PROSICS), HUVH, Vall d'Hebron Barcelona Hospital Campus, Universitat Autónoma de Barcelona, Barcelona, Spain. [279]Hospital Clínico San Carlos and IdSSC, Madrid, Spain. [280]Faculdades Pequeno Príncipe, Instituto de Pesquisa Pelé Pequeno Príncipe, Curitiba, Brazil. [281]AP-HP, Avicenne Hospital, Intensive Care Unit, Bobigny, France. [282]Service de Médecine Intensive Réanimation, Institut de Cardiologie, Hopital Pitié-Salpêtrière, Paris, France. [283]Department of Pediatrics, Medizinische Fakultät Carl Gustav Carus, Technische Universität Dresden, Dresden, Germany. [284]CHRU de Nancy, Hôpital d'Enfants, Vandoeuvre, France. [285]Chair of Nephrology, University of Brescia, Brescia, Italy. [286]Department of Immunology, 2nd Faculty of Medicine, Charles University and Motol University Hospital, Prague, Czech Republic. [287]Clínica Universidad de Navarra and Ciberes, Madrid, Spain. [288]HUS Helsinki University Hospital, Children and Adolescents, Rare Disease Center, and Inflammation Center, Adult Immunodeficiency Unit, Majakka, Helsinki, Finland. [289]Fundació Docencia i Recerca Mutua Terrassa, Terrassa, Spain. [290]D. Rogachev National Medical and Research Center of Pediatric Hematology, Oncology, Immunoogy, Moscow, Russia. [291]Haematology Laboratory, Lariboisière Hospital, University of Paris, Paris, France. [293]INSERM U1140, University of Paris, European Georges Pompidou Hospital, Paris, France. [294]Department of Pediatrics, Faculty of Medicine, Mansoura University, Mansoura, Egypt. [295]Intensive Care Medicine, Hospital Universitario de Gran Canaria Dr. Negrín, Canarian Health System, Las Palmas de Gran Canaria, Spain. [296]CHU de Saint Etienne, Saint-Priest-en-Jarez, France. [297]Pediatric Infectious Diseases and Immunodeficiencies Unit, Hospital Universitari Vall d'Hebron, VHIR, Vall d'Hebron Barcelona Hospital Campus, Universitat Autònoma de Barcelona (UAB), Barcelona, Catalonia, Spain, EU, Barcelona, Spain. [298]Department of pediatric infectious diseases and pediatric immunology, Shupyk National Healthcare University of Ukraine, Kyiv, Ukraine. [299]Gustave Roussy Cancer Campus, Villejuif, France. [300]Intensive Care Unit, Avicenne Hospital, APHP, Bobigny, France. [301]Laboratory of Immunology and Histocompatibility, Saint-Louis Hospital, Paris University, Paris, France. [302]Center for Inflammation Research, Laboratory of Molecular Signal Transduction in Inflammation, VIB, Ghent, Belgium. [303]Department of Internal Medicine, Université de Paris, INSERM, U970, PARCC, F-75015, Paris, France. [304]Service de médecine intensive réanimation, CHU de Saint-Étienne, France. [305]Department of Rheumatology and Inflammation Research, Institute of Medicine, Sahlgrenska Academy, University of Gothenburg, Gothenburg, Sweden. [306]University of Management and Technology, Lahore, Pakistan. [307]Department of Infectious Diseases, Aarhus University Hospital, Aarhus, Denmark. [308]First Division of Anesthesiology and Critical Care Medicine, ASST Spedali Civili di Brescia, Brescia, Italy. [309]Intensive Care Department, Hospital Universitari MutuaTerrassa, Universitat Barcelona, Terrassa, Spain. [310]Laboratory of Immunobiology, Center for Clinical, Experimental Surgery and Translational Research, Biomedical Research Foundation of the Academy of Athens, Athens, Greece. [311]International Center of Research in Infectiology, Lyon University, INSERM U1111, CNRS UMR 5308, ENS, UCBL, Lyon, France; Hospices Civils de Lyon, Lyon Sud Hospital, Pierre-Bénite,

France. [312]Infanta Leonor University Hospital, Madrid, Spain. [313]Department of Medicine and Geriatrics, Princess Margaret Hospital, Hong Kong. [314]University of Tartu, Institute of Clinical Medicine, Tartu, Estonia. [315]Department of Medicine, United Christian Hospital, Hong Kong. [316]Hematology Department, ASST Spedali Civili di Brescia, Brescia, Italy. [317]Pneumologie, Hôpital Avicenne, APHP, INSERM U1272, Université Sorbonne Paris Nord, Bobigny, France. [318]Dermatology unit, Laboratoire GAD, INSERM UMR1231 LNC, Université de Bourgogne, Dijon, France. [319]University Hospital of Burgos, Burgos, Spain. [320]Center of Human Genetics, Hôpital Erasme, Université Libre de Bruxelles, Brussels, Belgium. [321]Bellvitge University Hospital, L'Hospitalet de Llobregat, Barcelona, Spain. [322]University of São Paulo, São Paulo, Brazil. [323]CHU de Caen, Caen, France. [324]Hospital del Mar-IMIM Biomedical Research Institute, Barcelona, Catalonia, Spain. [325]Neglected Human Genetics Laboratory, INSERM, University of Paris, Paris, France. [326]Sorbonne Université, Service de Médecine Intensive Réanimation, Hôpital Tenon, AP-HP, Paris, France. [327]Pediatric Infectious Disease and Pediatric Immunology Department, Shupyk National Healthcare University of Ukraine, Kyiv, Ukraine. [328]Department of Pneumology, University Hospitals Leuven, Leuven, Belgium. [329]Laboratory for Clinical Infectious and Inflammatory Disorders, Departement of Microbiology, Immunology and Transplantation, Leuven, Belgium. [330]Department of Clinical Pathology, Pamela Youde Nethersole Eastern Hospital, Hong Kong. [331]Department of Medicine, Queen Elizabeth Hospital, Hong Kong. [332]Ankara City Hospital, Children's Hospital, Ankara, Turkey. [333]Division of Pediatric Infectious Disease, Department of Pediatrics, Faculty of Medicine, Karadeniz Technical University, Department of Pediatrics, Division of Pediatric Critical Care Medicine, Trabzon, Turkey. [334]Health Sciences University, Lütfi Kırdar Kartal Education and Research Hospital, İstanbul, Turkey. [335]Department of Immunology, Hospital Ramón y Cajal, Madrid, Spain. [336]Department of Nephrology and Infectiology, AZ Sint-Jan, Bruges, Belgium. [337]Department of Pulmonology, Ghent University Hospital, Belgium. [338]Department of Pediatric Pulmonology and Immunology, Ghent University Hospital, Belgium. [339]Department of Intensive Care Unit, Ghent University Hospital, Belgium. [340]Department of Pediatric Hemato-Oncology, Jolimont Hospital; Department of Pediatric Hemato-Oncology, HUDERF, La Louvière, Belgium. [341]Department of Pulmonology, ZNA Middelheim, Antwerp, Belgium. [342]Department of Internal Medicine, Ghent University Hospital, Belgium. [343]Department of Pediatric Immuno-Hémato-Rhumatology, CHR Citadelle, Liége, Belgium. [344]Department of Pediatric Hemato-Oncology, UCL Louvain, Brussels, Belgium. [345]Department of Pediatrics, Saint Luc, UCL Louvain, Brussels, Belgium.

Members of the COVID-STORM Clinicians: Giuseppe Foti[1], Giacomo Bellani[1], Giuseppe Citerio[1], Ernesto Contro[1], Alberto Pesci[2], Maria Grazia Valsecchi[3], Marina Cazzaniga[4]

[1]Department of Emergency, Anesthesia and Intensive Care, School of Medicine and Surgery, University of Milano-Bicocca, San Gerardo Hospital, Monza, Italy. [2]Department of Pneumology, School of Medicine and Surgery, University of Milano-Bicocca, San Gerardo Hospital, Monza, Italy. [3]Center of Bioinformatics, Biostatistics and Bioimaging, School of Medicine and Surgery, University of Milano-Bicocca, San Gerardo Hospital, Monza, Italy. [4]Phase I Research Center, School of Medicine and Surgery, University of Milano-Bicocca, San Gerardo Hospital, Monza, Italy.

Members of the NIAID Immune Response to COVID Group: Jeffrey J. Danielson[1], Kerry Dobbs[1], Anuj Kashyap[1], Li Ding[1], Clifton L. Dalgard[2], Alessandra Sottini[3], Virginia Quaresima[3], Eugenia Quiros-Roldan[4], Camillo Rossi[5], Laura Rachele Bettini[6], Mariella D'Angio[6], Ilaria Beretta[7], Daniela Montagna[8], Amelia Licari[9], Gian Luigi Marseglia[9]

[1]Laboratory of Clinical Immunology and Microbiology, Division of Intramural Research, NIAID, NIH, Bethesda, MD, USA. [2]Department of Anatomy, Physiology and Genetics, Uniformed Services University of the Health Sciences; The American Genome Center, Uniformed Services University of the Health Sciences, Bethesda, MD, USA. [3]CREA Laboratory, Diagnostic Department, ASST Spedali Civili di Brescia, Brescia, Italy. [4]Department of Infectious and Tropical Diseases, University of Brescia and ASST Spedali Civili di Brescia, Brescia, Italy. [5]Chief Medical Officer, ASST Spedali Civili di Brescia, Brescia, Italy. [6]Pediatric Departement and Centro Tettamanti-European Reference Network PaedCan, EuroBloodNet, MetabERN-University of Milano-Bicocca-Fondazione MBBM-

Ospedale, San Gerardo, Monza, Italy. [7]Department of Infectious Diseases, University of Milano-Bicocca, San Gerardo Hospital, Monza, Italy. [8]Laboratory of Immunology and Transplantation, Fondazione IRCCS Policlinico San Matteo, Pavia, Italy; Department of Clinical, Surgical, Diagnostic and Pediatric Sciences, University of Pavia, Pavia, Italy. [9]Pediatric Clinic, Fondazione IRCCS Policlinico San Matteo, Pavia, Italy; Department of Clinical, Surgical, Diagnostic and Pediatric Sciences, University of Pavia, Pavia, Italy.

Members of the NH-COVAIR Study Group: Isabella Batten[1], Conor Reddy[1], Matt McElheron[1], Claire Noonan[1], Emma Connolly[1], Aoife Fallon[1]

[1]Department of Age-Related Healthcare, Tallaght University Hospital and Department of Medical Gerontology, School of Medicine, Trinity College Dublin.

Members of the Danish CHGE: Merete Storgaard[1], Sofie Jørgensen[1], Martin Tolstrup[1]

[1]Department of Infectious Diseases, Aarhus University Hospital, Aarhus, Denmark.

Members of the The Danish Blood Donor Study (DBDS): Christian Erikstrup[1], Ole Birger Pedersen[2], Erik Sørensen[3], Susan Mikkelsen[1], Khoa Manh Dinh[1], Margit Anita Hørup Larsen[3], Isabella Worlewenut Paulsen[2], Jakob Hjorth Von Stemann[3], Morten Bagge Hansen[3], Sisse Rye Ostrowski[3]

[1]Department of Clinical Immunology, Aarhus University Hospital, Aarhus, Denmark. [2]Department of Clinical Immunology, Zeeland University Hospital, Køge, Denmark. [3]Department of Clinical Immunology, Rigshospitalet, Copenhagen University Hospital, Copenhagen, Denmark.

Members of the St James's Hospital, SARS CoV2 Interest group: Liam Townsend[1], Cliona Ni Cheallaigh[1], Colm Bergin[1], Ignacio Martin-Loeches[2], Jean Dunne[3], Niall Conlon[3], Nollaig Bourke[4], Cliona O'Farrelly[5]

[1]Department of Infectious Diseases, St James's Hospital; Department of Clinical Medicine, School of Medicine, Trinity Translational Medicine Institute, Trinity College Dublin, Dublin, Ireland. [2]Department of Intensive Care Medicine, St James's Hospital, Dublin, Ireland. [3]Department of Immunology, St James's Hospital; Department of Immunology, School of Medicine, Trinity College Dublin, Ireland. [4]Department of Medical Gerontology, School of Medicine, Trinity Translational Medicine Institute, Trinity College Dublin, Dublin, Ireland. [5]School of Biochemistry and Immunology, Trinity Biomedical Sciences Institute, Trinity College Dublin; School of Medicine, Trinity College Dublin, Dublin, Ireland.

Members of the French COVID Cohort Study Group: Laurent Abel[1], Clotilde Allavena[2], Claire Andrejak[3], François Angoulvant[4], Cecile Azoulay[5], Delphine Bachelet[6], Marie Bartoli[7], Romain Basmaci[8], Sylvie Behillill[9], Marine Beluze[10], Nicolas Benech[11], Dehbia Benkerrou[12], Krishna Bhavsar[6], Laurent Bitker[11], Lila Bouadma[6], Maude Bouscambert-Duchamp[13], Pauline Caraux Paz[14], Minerva Cervantes-Gonzalez[6], Anissa Chair[6], Catherine Chirouze[15], Alexandra Coelho[16], Hugues Cordel[17], Camille Couffignal[6], Sandrine Couffin-Cadiergues[18], Eric d'Ortenzio[7], Etienne De Montmollin[6], Alexa Debard[19], Marie-Pierre Debray[6], Dominique Deplanque[20], Diane Descamps[6], Mathilde Desvallée[21], Alpha Diallo[7], Jean-Luc Diehl[22], Alphonsine Diouf[16], Céline Dorival[12], François Dubos[23], Xavier Duval[6], Philippine Eloy[6], Vincent Enouf[9], Olivier Epaulard[24], Hélène Esperou[18], Marina Esposito-Farase[6], Manuel Etienne[25], Denis Garot[26], Nathalie Gault[6], Alexandre Gaymard[13], Jade Ghosn[6], Tristan Gigante[27], Morgane Gilg[27], François Goehringer[28], Jérémie Guedj[29], Alexandre Hoctin[16], Isabelle Hoffmann[6], Ikram Houas[18], Jean-Sébastien Hulot[22], Salma Jaafoura[18], Ouifiya Kafif[6], Florentia Kaguelidou[30], Sabrina Kali[6], Younes Kerroumi[31], Antoine Khalil[6], Coralie Khan[21], Antoine Kimmoun[32], Fabrice Laine[33], Cédric Laouénan[6], Samira Laribi[6], Minh Le[6], Cyril Le Bris[34], Sylvie Le Gac[6], Quentin Le Hingrat[6], Soizic Le Mestre[7], Hervé Le Nagard[35], Adrien Lemaignen[26], Véronique Lemee[25], François-Xavier Lescure[6], Sophie Letrou[6], Yves Levy[6], Bruno Lina[13], Guillaume Lingas[35], Jean Christophe Lucet[6], Moïse Machado[37], Denis Malvy[38], Marina

Mambert[16], Aldric Manuel[39], France Mentré[6], Amina Meziane[12], Hugo Mouquet[9], Jimmy Mullaert[6], Nadège Neant[35], Duc Nguyen[38], Marion Noret[40], Aurélie Papadopoulos[18], Christelle Paul[7], Nathan Peiffer-Smadja[6], Vincent Peigne[41], Ventzislava Petrov-Sanchez[7], Gilles Peytavin[6], Huong Pham[6], Olivier Picone[8], Valentine Piquard[6], Julien Poissy[23], Oriane Puéchal[42], Manuel Rosa-Calatrava[13], Bénédicte Rossignol[27], Patrick Rossignol[28], Carine Roy[6], Marion Schneider[6], Richa Su[6], Coralie Tardivon[6], Marie-Capucine Tellier[6], François Téoulé[12], Olivier Terrier[13], Jean-François Timsit[6], Christelle Tual[43], Sarah Tubiana[6], Sylvie Van Der Werf[9], Noémie Vanel[44], Aurélie Veislinger[43], Benoit Visseaux[6], Aurélie Wiedemann[45], Yazdan Yazdanpanah[6]

[1]INSERM UMR 1163, Paris, France. [2]CHU Nantes, France. [3]CHU Amiens, France. [4]Hôpital Necker, Paris, France. [5]Hopitâl Cochin, Paris, France. [6]Hôpital Bichat, Paris, France. [7]ANRS, Paris, France. [8]Hôpital Louis Mourier, Colombes, France. [9]Pasteur Institute, Paris, France. [10]F-CRIN Partners Platform, Paris, France. [11]CHU Lyon, France. [12]INSERM UMR 1136, Paris, France. [13]INSERM UMR 1111, Lyon, France. [14]CH Villeneuve Saint Georges, France. [15]CHRU Jean Minjoz, Besançon, France. [16]INSERM UMR 1018, Paris, France. [17]Hôpital Avicenne, Bobigny, France. [18]INSERM Pôle Recherche Clinique, Paris, France. [19]CHU Toulouse, France. [20]Hôpital Calmette, Lille, France. [21]INSERM UMR 1219, Bordeaux, France. [22]Hôpital Européen Georges Pompidou, Paris, France. [23]CHU Lille, France. [24]CHU Grenoble, France. [25]CHU Rouen, France. [26]CHU Tours, France. [27]F-CRIN INI-CRCT, Nancy, France. [28]CHU Nancy, France. [29]Université de Paris, INSERM, IAME, F-75018 Paris, France. [30]Hôpital Robert Debré, Paris, France. [31]GH Diaconesses, Paris, France. [32]Université de Lorraine, CHRU de Nancy, Service de Médecine Intensive et Réanimation Brabois, INSERM U116, Nancy, France. [33]CHU Rennes, France. [34]CH Beziers, France. [35]INSERM UMR 1137, Paris, France. [36]Vaccine Research Insitute (VRI), INSERM U955, Créteil, France. [37]Grand Hôpital de l'Est Francilien, Marne-la-Vallée, France. [38]CHU Bordeaux, France. [39]CH Annecy, France. [40]RENARCI, Annecy, France. [41]CH Métropole Savoie, Cambery, France. [42]REACTing, Paris, France. [43]INSERM CIC-1414, Rennes, France. [44]Hôpital la Timone, Marseille, France. [45]Vaccine Research Insitute (VRI), INSERM UMR 955, Créteil, France.

Members of the Imagine COVID-Group: Jean-Philippe Annereau[1], Luis Briseño-Roa[1], Olivier Gribouval[2], Jean-Philippe Jaïs[2,3], Anna Pelet[2]

[1]Medetia Pharmaceuticals, Paris, France. [2]Imagine Institute, Université de Paris, INSERM UMR 1163, Paris, France. [3]Department of Biostatistics, Hôpital Necker, University of Paris, Paris, France.

Members of the The Milieu Intérieur Consortium: Laurent Abel[1], Andres Alcover[2], Hugues Aschard[2], Philippe Bousso[2], Nollaig Bourke[3], Petter Brodin[4], Pierre Bruhns[2], Nadine Cerf-Bensussan[5], Ana Cumano[2], Christophe D'Enfert[2], Ludovic Deriano[2], Marie-Agnès Dillies[2], James Di Santo[2], Françoise Dromer[2], Gérard Eberl[2], Jost Enninga[2], Jacques Fellay[6], Ivo Gomperts-Boneca[2], Milena Hasan[2], Gunilla Karlsson Hedestam[4], Serge Hercberg[7], Molly A. Ingersoll[2], Olivier Lantz[8], Rose Anne Kenny[3], Mickaël Ménager[5], Frédérique Michel[2], Hugo Mouquet[2], Cliona O'Farrelly[3], Etienne Patin[2], Sandra Pellegrini[2], Antonio Rausell[5], Frédéric Rieux-Laucat[5], Lars Rogge[2], Magnus Fontes[9], Anavaj Sakuntabhai[2], Olivier Schwartz[2], Benno Schwikowski[2], Spencer Shorte[2], Frédéric Tangy[2], Antoine Toubert[10], Mathilde Touvier[7], Marie-Noëlle Ungeheuer[2], Christophe Zimmer[2], Matthew L. Albert[11], Darragh Duffy[2], Lluis Quintana-Murci[2]

[1]Hôpital Necker, Paris, France. [2]Institut Pasteur, Paris, France. [3]Trinity College, Dublin, Ireland. [4]Karolinska Institutet, Stockholm, Sweden. [5]INSERM U1163, Institut Imagine, Paris, France. [6]EPFL, Lausanne, Switzerland. [7]Sorbonne Paris Nord University, INSERM U1153, INRAE U1125, CNAM, Nutritional Epidemiology Research Team (EREN), Epidemiology and Statistics Research Center – University of Paris (CRESS), Bobigny, France.[8]Institut Curie, Paris, France. [9]Institut Roche, Paris, France. [10]Hôpital Saint-Louis, Paris, France. [11]In Sitro, San Francisco, USA.

Members of the CoV-Contact Cohort: Loubna Alavoine[1], Sylvie Behillil[2], Charles Burdet[3], Charlotte Charpentier[3,4], Aline Dechanet[5], Diane Descamps[3,6], Xavier Duval[1,3], Jean-Luc Ecobichon[1], Vincent Enouf[8], Wahiba Frezouls[1], Nadhira Houhou[5], Ouifiya Kafif[5], Jonathan Lehacaut[1], Sophie Letrou[1], Bruno Lina[9], Jean-Christophe Lucet[10], Pauline Manchon[5], Mariama

Nouroudine[1], Valentine Piquard[5], Caroline Quintin[1], Michael Thy[11], Sarah Tubiana[1], Sylvie van der Werf[8], Valérie Vignali[1], Benoit Visseaux[3,10], Yazdan Yazdanpanah[3,10], Abir Chahine[12], Nawal Waucquier[12], Maria-Claire Migaud[12], Dominique Deplanque[12], Félix Djossou[13], Mayka Mergeay-Fabre[14], Aude Lucarelli[15], Magalie Demar[13], Léa Bruneau[16], Patrick Gérardin[17], Adrien Maillot[16], Christine Payet[18], Bruno Laviolle[19], Fabrice Laine[19], Christophe Paris[19], Mireille Desille-Dugast[19], Julie Fouchard[19], Denis Malvy[20], Duc Nguyen[20], Thierry Pistone[20], Pauline Perreau[20], Valérie Gissot[21], Carole Le Goas[21], Samatha Montagne[22], Lucie Richard[23], Catherine Chirouze[24], Kévin Bouiller[24], Maxime Desmarets[25], Alexandre Meunier[26], Marilou Bourgeon[26], Benjamin Lefèvre[27], Hélène Jeulin[28], Karine Legrand[29], Sandra Lomazzi[30], Bernard Tardy[31], Amandine Gagneux-Brunon[32], Frédérique Bertholon[33], Elisabeth Botelho-Nevers[32], Christelle Kouakam[34], Nicolas Leturque[34], Layidé Roufai[34], Karine Amat[35], Sandrine Couffin-Cadiergues[34], Hélène Espérou[36], Samia Hendou[34].

[1]Centre d'Investigation Clinique, INSERM CIC 1425, Hôpital Bichat Claude Bernard, APHP, Paris, France. [2]Institut Pasteur, Paris, France. [3]Université de Paris, IAME, INSERM U1137, Paris, France, Hôpital Bichat Claude Bernard, APHP, Paris, France. [4]Service de Virologie, Université de Paris, INSERM, IAME, UMR 1137, AP-HP, Hôpital Bichat-Claude Bernard, F-75018 Paris, France. [6]IAME INSERM U1140, Hôpital Bichat Claude Bernard, APHP, Paris, France. [7]Centre d'Investigation Clinique, INSERM CIC 1425, APHP, IAME, Paris University, Paris, France. [8]Institut Pasteur, U3569 CNRS, Université de Paris, Paris, France. [9]Virpath Laboratory, International Center of Research in Infectiology, Lyon University, INSERM U1111, CNRS U5308, ENS, UCBL, Lyon, France. [10]IAME INSERM U1138, Hôpital Bichat Claude Bernard, APHP, Paris, France. [11]Center for Clinical Investigation, Assistance Publique-Hôpitaux de Paris, Bichat-Claude Bernard University Hospital, Paris, France. [12]Centre d'Investigation Clinique, INSERM CIC 1403, Centre Hospitalo Universitaire de Lille, Lille, France. [13]Center of Biological Resource (CRB Amazonie), Centre Hospitalier de Cayenne Andrée Rosemon, Guiana, France. [14]Centre d'Investigation Clinique, INSERM CIC 1424, Centre Hospitalier de Cayenne, Cayenne, Guyane Française. [15]Service Hôpital de jour Adulte, Centre Hospitalier de Cayenne, Guyane, France. [16]Centre d'Investigation Clinique, INSERM CIC 1410, Centre Hospitalo universitaire de la Réunion, La Réunion, France. [17]Centre d'Investigation Clinique, INSERM CIC 1410, CHU Reunion, Saint-Pierre, Reunion island. [18]Centre de Ressources Biologiques, Centre Hospitalo universitaire de la Réunion, La Réunion, France. [19]CRB Santé, INSERM U1241, Université de Rennes 1, Centre hospitalier universitaire de Rennes, Rennes, France. [20]Service des maladies infectieuses, Centre Hospitalo universitaire de Bordeaux, Bordeaux, France. [21]Centre d'Investigation Clinique, INSERM CIC 1415, CHRU Tours, Tours, France. [22]CRBT, Centre Hospitalo universitaire de Tours, Tours, France. [23]Pole de Biologie Médicale, Centre Hospitalo universitaire de Tours, Tours, France. [24]Service des maladies infectieuses, Centre Hospitalo universitaire de Besançon, Besançon, France. [25]Service des maladies infectieuses, Centre d'investigation clinique, INSERM CIC1431, Centre Hospitalier Universitaire de Besançon, Besançon, France. [26]Centre de Ressources Biologiques - Filière Microbiologique de Besançon, Centre Hospitalier Universitaire, Besançon, France. [27]Université de Lorraine, CHRU-Nancy and APEMAC, Infectious and tropical diseases, Nancy, France. [28]Laboratoire de Virologie, CHRU de Nancy Brabois, Vandoeuvre-lès-Nancy, France. [29]INSERM CIC-EC 1433, Centre Hospitalo universitaire de Nancy, Nancy, France. [30]Centre de ressources Biologiques, Centre Hospitalo universitaire de Nancy, Nancy, France. [31]Centre d'Investigation Clinique, INSERM CIC 1408, Centre Hospitalo universitaire de Saint-Étienne, Saint-Étienne, France. [32]Service des maladies infectieuses, Centre Hospitalo universitaire de Saint-Étienne, Saint-Étienne, France. [33]Service des maladies infectieuses, CRB[42]-BTK, Centre Hospitalo Universitaire de Saint-Étienne, Saint-Étienne, France. [34]Pole Recherche Clinique, INSERM, Paris France. [35]IMEA Fondation Léon M'Ba, Paris, France. [36]INSERM Pôle Recherche Clinique, Paris, France.

Members of the Amsterdam UMC Covid-19 Biobank: Michiel van Agtmael[2], Anne Geke Algera[1], Brent Appelman[2], Frank van Baarle[1], Diane Bax[3], Martijn Beudel[4], Harm Jan Bogaard[5], Marije Bomers[2], Peter Bonta[5], Lieuwe Bos[1], Michela Botta[1], Justin de Brabander[2], Godelieve de Bree[2], Sanne de Bruin[1], David T. P. Buis[1], Marianna Bugiani[5], Esther Bulle[1], Osoul Chouchane[2] Alex Cloherty[3], Mirjam Dijkstra[12], Dave A. Dongelmans[1], Romein W. G. Dujardin[1], Paul Elbers[1], Lucas Fleuren[1], Suzanne Geerlings[2] Theo Geijtenbeek[3], Armand Girbes[1], Bram Goorhuis[2], Martin P.

Grobusch[2], Florianne Hafkamp[3], Laura Hagens[1], Jorg Hamann[7], Vanessa Harris[2], Robert Hemke[8], Sabine M. Hermans[2] Leo Heunks[1], Markus Hollmann[6], Janneke Horn[1], Joppe W. Hovius[2], Menno D. de Jong[9], Rutger Koning[4], Endry H. T. Lim[1], Niels van Mourik[1], Jeaninne Nellen[2], Esther J. Nossent[5], Frederique Paulus[1], Edgar J.G. Peters[2], Dan A. I. Pina-Fuentes[4], Tom van der Poll[2], Bennedikt Preckel[6], Jan M. Prins[2], Jorinde Raasveld[1], Tom Reijnders[2], Maurits C. F. J. de Rotte[12], Michiel Schinkel[2], Marcus J. Schultz[1], Femke A. P. Schrauwen[12], Alex Schuurman[10], Jaap Schuurmans[1], Kim Sigaloff[1], Marleen A. Slim[1,2], Patrick Smeele[5], Marry Smit[1], Cornelis S. Stijnis[2], Willemke Stilma[1], Charlotte Teunissen[11], Patrick Thoral[1], Anissa M. Tsonas[1], Pieter R. Tuinman[2], Marc van der Valk[2], Denise Veelo[6], Carolien Volleman[1], Heder de Vries[1], Lonneke A. Vught[1,2], Michèle van Vugt[2], Dorien Wouters[12], A. H. (Koos) Zwinderman[13], Matthijs C. Brouwer[4], W. Joost Wiersinga[2], Alexander P. J. Vlaar[1], Diederik van de Beek[4]

[1]Department of Intensive Care, Amsterdam UMC, Amsterdam, Netherlands. [2]Department of Infectious Diseases, Amsterdam UMC, Amsterdam, Netherlands. [3]Experimental Immunology, Amsterdam UMC, Amsterdam, Netherlands. [4]Department of Neurology, Amsterdam UMC, Amsterdam Neuroscience, Amsterdam, Netherlands. [5]Department of Pulmonology, Amsterdam UMC, Amsterdam, Netherlands. [6]Department of Anesthesiology, Amsterdam UMC, Amsterdam, Netherlands. [7]Amsterdam UMC Biobank Core Facility, Amsterdam UMC, Amsterdam, Netherlands. [8]Department of Radiology, Amsterdam UMC, Amsterdam, Netherlands. [9]Department of Medical Microbiology, Amsterdam UMC, Amsterdam, Netherlands. [10]Department of Internal Medicine, Amsterdam UMC, Amsterdam, Netherlands. [11]Neurochemical Laboratory, Amsterdam UMC, Amsterdam, Netherlands. [12]Department of Clinical Chemistry, Amsterdam UMC, Amsterdam, Netherlands. [13]Department of Clinical Epidemiology, Biostatistics and Bioinformatics, Amsterdam UMC, Amsterdam, Netherlands.

Members of the COVID Human Genetic Effort: Laurent Abel[1], Alessandro Aiuti[2], Saleh Al-Muhsen[3], Fahd Al-Mulla[4], Mark S. Anderson[5], Evangelos Andreakos[6], Andrés A. Arias[7], Hagit Baris Feldman[8], Alexandre Belot[9], Catherine M. Biggs[10], Dusan Bogunovic[11], Alexandre Bolze[12], Anastasiia Bondarenko[13], Ahmed A. Bousfiha[14], Petter Brodin[15], Yenan Bryceson[16], Carlos D. Bustamante[17], Manish J. Butte[18], Giorgio Casari[19], Samya Chakravorty[20], John Christodoulou[21], Antonio Condino-Neto[22], Stefan N. Constantinescu[23], Megan A. Cooper[24], Clifton L. Dalgard[25], Murkesh Desai[26], Beth A. Drolet[27], Jamila El Baghdadi[28], Sara Espinosa-Padilla[29], Jacques Fellay[30], Carlos Flores[31], José Luis Franco[7], Antoine Froidure[32], Peter K. Gregersen[33], Filomeen Haerynck[34], David Hagin[35], Rabih Halwani[36], Lennart Hammarström[37], James R. Heath[38], Sarah E. Henrickson[39], Elena W. Y. Hsieh[40], Eystein S. Husebye[41], Kohsuke Imai[42], Yuval Itan[43], Erich D. Jarvis[44], Timokratis Karamitros[45], Kai Kisand[46], Cheng-Lung Ku[47], Yu-Lung Lau[48], Yun Ling[49], Carrie L. Lucas[50], Tom Maniatis[51], Davood Mansouri[52], László Maródi[53], Isabelle Meyts[54], Joshua D. Milner[55], Kristina Mironska[56], Trine H. Mogensen[57], Tomohiro Morio[58], Lisa F. P. Ng[59], Luigi D. Notarangelo[60], Antonio Novelli[61], Giuseppe Novelli[62], Cliona O'Farrelly[63], Satoshi Okada[64], Tayfun Ozcelik[65], Qiang Pan-Hammarström[37], Rebeca Perez de Diego[66], Anna M. Planas[67], Jordi Perez-Tur[99], Lisa M. Arkin[100], Takaki Asano[101], Roger Colobran Oriol[102], Carolina Prando[68], Aurora Pujol[69], Lluis Quintana-Murci[70], Laurent Renia[59], Igor Resnick[71], Carlos Rodríguez-Gallego[72], Vanessa Sancho-Shimizu[73], Anna Sediva[74], Mikko R.J. Seppänen[75], Mohammed Shahrooei[76], Anna Shcherbina[77], Ondrej Slaby[78], Andrew L. Snow[79], Pere Soler-Palacín[80], András N. Spaan[81], Ivan Tancevski[82], Stuart G. Tangye[83], Ahmad Abou Tayoun[84], Sathishkumar Ramaswamy[84], Stuart E. Turvey[85], K. M. Furkan Uddin[86], Mohammed J. Uddin[87], Diederik van de Beek[88], Donald C. Vinh[89], Horst von Bernuth[90], Mayana Zatz[91], Pawel Zawadzki[92], Bodo Grimbacher[93], Keisuke Okamoto[94], Jean W. Pape[95], David S. Perlin[96], Graziano Pesole[97], Joost Wauters[98], Helen C. Su[60], Jean-Laurent Casanova[103]

[1]INSERM U1163, University of Paris, Imagine Institute, Paris, France. [2]San Raffaele Telethon Institute for Gene Therapy, IRCCS Ospedale San Raffaele, and Vita Salute San Raffaele University, Milan, Italy. [3]Immunology Research Laboratory, Department of Pediatrics, College of Medicine and King Saud University Medical City, King Saud University, Riyadh, Saudi Arabia. [4]Dasman Diabetes Institute, Department of Genetics and Bioinformatics, Dasman, Kuwait. [5]Diabetes Center, University of California San Francisco, San Francisco, CA, USA. [6]Laboratory of Immunobiology, Center for

Clinical, Experimental Surgery and Translational Research, Biomedical Research Foundation of the Academy of Athens, Athens, Greece. [7]Group of Primary Immunodeficiencies, University of Antioquia UdeA, Medellin, Colombia. [8]The Genetics Institute, Tel Aviv Sourasky Medical Center and Sackler Faculty of Medicine, Tel Aviv University, Tel Aviv, Israel. [9]Pediatric Nephrology, Rheumatology, Dermatology, HFME, Hospices Civils de Lyon, National Referee Centre RAISE, and INSERM U1111, Université de Lyon, Lyon, France. [10]Department of Pediatrics, British Columbia Children's Hospital, The University of British Columbia, Vancouver, BC, Canada [11]Icahn School of Medicine at Mount Sinai, New York, NY, USA. [12]Helix, San Mateo, CA, USA. [13]Shupyk National Medical Academy for Postgraduate Education, Kiev, Ukraine. [14]Clinical Immunology Unit, Department of Pediatric Infectious Disease, CHU Ibn Rushd and LICIA, Laboratoire d'Immunologie Clinique, Inflammation et Allergie, Faculty of Medicine and Pharmacy, Hassan II University, Casablanca, Morocco. [15]SciLifeLab, Department of Women's and Children's Health, Karolinska Institutet, Stockholm, Sweden [16]Department of Medicine, Center for Hematology and Regenerative Medicine, Karolinska Institutet, Stockholm, Sweden. [17]Stanford University, Stanford, CA, USA. [18]Division of Immunology, Allergy, and Rheumatology, Department of Pediatrics and the Department of Microbiology, Immunology, and Molecular Genetics, University of California, Los Angeles, CA, USA. [19]Clinical Genomics, IRCCS San Raffaele Scientific Institute and Vita-Salute San Raffaele University, Milan, Italy [20]Department of Pediatrics and Children's Healthcare of Atlanta, Emory University, Atlanta, GA, USA. [21]Murdoch Children's Research Institute and Department of Paediatrics, University of Melbourne, Australia [22]Department of Immunology, Institute of Biomedical Sciences, University of São Paulo, São Paulo, Brazil. [23]de Duve Institute and Ludwig Cancer Research, Brussels, Belgium [24]Washington University School of Medicine, St. Louis, MO, USA. [25]Department of Anatomy, Physiology & Genetics, Uniformed Services University of the Health Sciences, Bethesda, MD, USA. [26]Bai Jerbai Wadia Hospital for Children, Mumbai, India. [27]School of Medicine and Public Health, University of Wisconsin, Madison, WI, USA. [28]Genetics Unit, Military Hospital Mohamed V, Rabat, Morocco. [29]Instituto Nacional de Pediatria (National Institute of Pediatrics), Mexico City, Mexico. [30]School of Life Sciences, Ecole Polytechnique Fédérale de Lausanne, Lausanne, Switzerland; Precision Medicine Unit, Lausanne University Hospital and University of Lausanne, Lausanne, Switzerland. [31]Genomics Division, Instituto Tecnológico y de Energías Renovables (ITER), Santa Cruz de Tenerife, Spain; Research Unit, Hospital Universitario N.S. de Candelaria, Santa Cruz de Tenerife, Spain; Instituto de Tecnologías Biomédicas (ITB), Universidad de La Laguna, San Cristóbal de La Laguna, Spain; CIBER de Enfermedades Respiratorias, Instituto de Salud Carlos III, Madrid, Spain. [32]Pulmonology Department, Cliniques Universitaires Saint-Luc; Institut de Recherche Expérimentale et Clinique (IREC), Université Catholique de Louvain, Brussels, Belgium. [33]Feinstein Institute for Medical Research, Northwell Health USA, Manhasset, NY, USA. [34]Department of Paediatric Immunology and Pulmonology, Centre for Primary Immunodeficiency Ghent (CPIG), PID Research Laboratory, Jeffrey Modell Diagnosis and Research Centre, Ghent University Hospital, Ghent, Belgium. [35]The Genetics Institute Tel Aviv Sourasky Medical Center, Tel Aviv, Israel. [36]Sharjah Institute of Medical Research, College of Medicine, University of Sharjah, Sharjah, United Arab Emirates. [37]Department of Biosciences and Nutrition, Karolinska Institutet, Stockholm, Sweden. [38]Institute for Systems Biology, Seattle, WA, USA. [39]Department of Pediatrics, Division of Allergy Immunology, Children's Hospital of Philadelphia, Philadelphia, PA, USA; Department of Microbiology, Perelman School of Medicine, University of Pennsylvania, Philadelphia, PA, USA. [40]Departments of Pediatrics, Immunology and Microbiology, University of Colorado, School of Medicine, Aurora, Colorado, USA. [41] Department of Clinical Science and K.G. Jebsen Center for Auoimmune Diseases, University of Bergen, Bergen, Norway. [42]Department of Community Pediatrics, Perinatal and Maternal Medicine, Tokyo Medical and Dental University (TMDU) [43]Institute for Personalized Medicine, Icahn School of Medicine at Mount Sinai, New York, NY, USA; Department of Genetics and Genomic Sciences, Icahn School of Medicine at Mount Sinai, New York, NY, USA. [44]Laboratory of Neurogenetics of Language and Howard Hughes Medical Institute, The Rockefeller University, New York, NY, USA. [45]Bioinformatics and Applied Genomics Unit, Hellenic Pasteur Institute, Athens, Greece [46]Molecular Pathology, Department of Biomedicine, Institute of Biomedicine and Translational Medicine, University of Tartu, Tartu Estonia. [47]Chang Gung University, Taoyuan County, Taiwan. [48]Department of Paediatrics & Adolescent Medicine, The University of Hong Kong, Hong Kong, China. [49]Shanghai Public Health Clinical Center, Fudan University, Shanghai, China. [50]Department of Immunobiology, Yale

University School of Medicine, New Haven, CT, USA. [51]Columbia University Zuckerman Institute, New York, NY, USA. [52]Department of Clinical Immunology and Infectious Diseases, National Research Institute of Tuberculosis and Lung Diseases, The Clinical Tuberculosis and Epidemiology Research Center, National Research Institute of Tuberculosis and Lung Diseases (NRITLD), Masih Daneshvari Hospital, Shahid Beheshti University of Medical Sciences, Tehran, Iran. [53]Primary Immunodeficiency Clinical Unit and Laboratory, Department of Dermatology, Venereology and Dermatooncology, Semmelweis University, Budapest, Hungary. [54]Department of Pediatrics, University Hospitals Leuven, Department of Microbiology, Immunology and Transplantation, and Laboratory for Inborn Errors of Immunity, KU Leuven, Leuven, Belgium. [55]Department of Pediatrics, Columbia University Irving Medical Center, New York, NY, USA. [56]University Clinic for Children's Diseases, Department of Pediatric Immunology, Medical Faculty, University "St.Cyril and Methodij" Skopje, North Macedonia. [57]Department of Biomedicine, Aarhus University, Aarhus, Denmark [58]Tokyo Medical & Dental University Hospital, Tokyo, Japan. [59]A*STAR Infectious Disease Labs, Agency for Science, Technology and Research, Singapore; Lee Kong Chian School of Medicine, Nanyang Technology University, Singapore. [60]National Institute of Allergy and Infectious Diseases, National Institutes of Health, Bethesda, MD, USA. [61]Laboratory of Medical Genetics, IRCCS Bambino Gesù Children's Hospital, Rome, Italy. [62]Department of Biomedicine and Prevention, Tor Vergata University of Rome, Rome, Italy. [63]Comparative Immunology Group, School of Biochemistry and Immunology, Trinity Biomedical Sciences Institute, Trinity College Dublin, Ireland. [64]Department of Pediatrics, Graduate School of Biomedical and Health Sciences, Hiroshima University, Hiroshima, Japan. [65]Department of Molecular Biology and Genetics, Bilkent University, Bilkent, Ankara, Turkey. [66]Laboratory of Immunogenetics of Human Diseases, Innate Immunity Group, IdiPAZ Institute for Health Research, La Paz Hospital, Madrid, Spain. [67]IIBB-CSIC, IDIBAPS, Barcelona, Spain. [68]Faculdades Pequeno Príncipe, Instituto de Pesquisa Pelé Pequeno Príncipe, Curitiba, Brazil. [69]Neurometabolic Diseases Laboratory, Bellvitge Biomedical Research Institute (IDIBELL), L'Hospitalet de Llobregat, Barcelona, Spain; Catalan Institution of Research and Advanced Studies (ICREA), Barcelona, Spain; Center for Biomedical Research on Rare Diseases (CIBERER), ISCIII, Barcelona, Spain. [70]Human Evolutionary Genetics Unit, CNRS U2000, Institut Pasteur, Paris, France; Human Genomics and Evolution, Collège de France, Paris, France. [71]Department of Medical Genetics, Medical University, Varna and Department Hematology and BMT, University Hospital St. Marina, Bulgaria. [72]Department of Immunology, University Hospital of Gran Canaria Dr. Negrín, Canarian Health System, Las Palmas de Gran Canaria, Spain; Department of Clinical Sciences, University Fernando Pessoa Canarias, Las Palmas de Gran Canaria, Spain [73]Department of Paediatric Infectious Diseases and Virology, Imperial College London, London, UK; Centre for Paediatrics and Child Health, Faculty of Medicine, Imperial College London, London, UK. [74]Department of Immunology, Second Faculty of Medicine Charles University, V Uvalu, University Hospital in Motol, Prague, Czech Republic. [75]Adult Immunodeficiency Unit, Infectious Diseases, Inflammation Center, University of Helsinki and Helsinki University Hospital, Helsinki, Finland; Rare Diseases Center and Pediatric Research Center, Children's Hospital, University of Helsinki and Helsinki University Hospital, Helsinki, Finland [76]Saeed Pathobiology and Genetics Lab, Tehran, Iran; Department of Microbiology and Immunology, Clinical and Diagnostic Immunology, KU Leuven, Leuven, Belgium. [77]Department of Immunology, Dmitry Rogachev National Medical Research Center of Pediatric Hematology, Oncology and Immunology, Moscow, Russia. [78]Central European Institute of Technology & Department of Biology, Faculty of Medicine, Masaryk University, Brno, Czech Republic. [79]Department of Pharmacology & Molecular Therapeutics, Uniformed Services University of the Health Sciences, Bethesda, MD, USA. [80]Pediatric Infectious Diseases and Immunodeficiencies Unit, Vall d'Hebron Barcelona Hospital Campus, Barcelona, Spain. [81]St. Giles Laboratory of Human Genetics of Infectious Diseases, Rockefeller Branch, The Rockefeller University, New York, NY, USA; Department of Medical Microbiology, University Medical Center Utrecht, Utrecht, Netherlands [82]Department of Internal Medicine II, Medical University of Innsbruck, Innsbruck, Austria. [83]Garvan Institute of Medical Research, Darlinghurst, NSW, Australia; St Vincent's Clinical School, Faculty of Medicine, UNSW Sydney, NSW, Australia. [84]Al Jalila Children's Hospital, Dubai, UAE [85]BC Children's Hospital, The University of British Columbia, Vancouver, Canada [86]Centre for Precision Therapeutics, Genetic and Genomic Medicine Centre, NeuroGen Children Healthcare, Dhaka, Bangladesh; Holy Family Red Crescent Medical College, Dhaka, Bangladesh. [87]College of Medicine, Mohammed Bin Rashid University of Medicine and Health

Sciences, Dubai, UAE; Cellular Intelligence (Ci) Lab, GenomeArc Inc., Toronto, ON, Canada [88]Department of Neurology, Amsterdam Neuroscience, Amsterdam University Medical Center, University of Amsterdam, Amsterdam, The Netherlands. [89]Department of Medicine, Division of Infectious Diseases, McGill University Health Centre, Montréal, Québec, Canada; Infectious Disease Susceptibility Program, Research Institute, McGill University Health Centre, Montréal, Québec, Canada. [90]Department of Pediatric Pneumology, Immunology and Intensive Care, Charité Universitätsmedizin, Berlin University Hospital Center, Berlin, Germany; Labor Berlin GmbH, Department of Immunology, Berlin, Germany; Berlin Institutes of Health (BIH), Berlin-Brandenburg Center for Regenerative Therapies, Berlin, Germany. [91]Biosciences Institute, University of São Paulo, São Paulo, Brazil. [92]Molecular Biophysics Division, Faculty of Physics, A. Mickiewicz University, Poznań, Poland. [93]Center for Chronic Immunodeficiency & Institute for Immunodeficiency, Medical Center, Faculty of Medicine, University of Freiburg, Freiburg, Germany. [94]Tokyo Medical and Dental University, Tokyo, Japan. [95]Haitian Study Group for Kaposi's Sarcoma and Opportunistic Infections (GHESKIO), Port-au-Prince, Haiti. [96]Center for Discovery and Innovation, Hackensack Meridian Health, Nutley, NJ, USA. [97]Department of Biosciences, Biotechnology and Biopharmaceutics, University of Bari A. Moro, Bari,Italy. [98]Department of General Internal Medicine, Medical Intensive Care Unit, University Hospitals Leuven, Leuven, Belgium. [99]Institut de Biomedicina de València-CSIC, CIBERNED, Unitat Mixta de Neurologia i Genètica, IIS La Fe, Vallencia, Spain. [100]Pediatric Dermatology, University of Wisconsin School of Medicine, Madison, WI, USA. [101]St. Giles Laboratory of Human Genetics of Infectious Diseases, Rockefeller Branch, The Rockefeller University, New York, NY, USA. [102]Hospital Universitari Vall d'Hebron (HUVH); Vall d'Hebron Institut de Recerca (VHIR), Barcelona, Spain [103]The Rockefeller University & Howard Hughes Medical Institute, New York, NY, USA; Necker Hospital for Sick Children & INSERM, Paris, France.

Members of the CP-COVID-19 Group: Paula Andrea Gaviria García[1,] Gustavo Andrés Salguero López[1,] Adriana Rojas-Villaraga[2,] Verónica Posada Vélez[3,] Lina Marcela Acevedo Landinez[1,] Luisa Paola Duarte Correales[1,] Oscar Gómez[4,] Jeser Santiago Grass Guaqueta[1,] Cristian Alejandro Ricaurte Pérez[1,] Jorge Carrillo[5,] José Alejandro Daza Vergara[5,] Sandra Landinez[5,] Rubén D. Mantilla[6,] Jairo David Torres Yepes[3,] Oscar Andrés Briceño Ricaurte[3,] Carlos E. Pérez-Díaz[7,] Yady Nataly Mateus[8,] Laura Mancera Navarro[8,] Yhojan Rodríguez[6,] Yeny Acosta-Ampudia[6,] Diana M. Monsalve[6,] Manuel Rojas[6]

[1]Instituto Distrital de Ciencia Biotecnología e Investigación en Salud, IDCBIS, Bogota, Colombia.[,] [2]Fundación Universitaria de Ciencias de la Salud, FUCS Bogota, Colombia.[,] [3]Internal Medicine Department, Universidad CES, Medellin, Colombia.[,] [4]Genoma CES, Universidad CES, Medellin, Colombia.[,] [5]Hospital Universitario Mayor Méderi, Bogota, Colombia.[,] [6]Center for Autoimmune Disease Research (CREA), School of Medicine and Health Sciences, Universidad del Rosario, Bogota, Colombia.[,] [7]Infectious diseases, Clínica de Marly, Bogota, Colombia.[,] [8]Clínica del Occidente, Bogota, Colombia.

Members of the CONSTANCES cohort: Rachel Nadif[1], Marcel Goldberg[2], Anna Ozguler[2], Joseph Henny[2], Sylvie Lemonnier[2], Mireille Coeuret-Pellicer[3], Stéphane Le Got[2], Marie Zins[2]

[1]Université de Paris-Saclay, UVSQ, Université Paris-Sud, Inserm, Equipe d'Epidémiologie Respiratoire Intégrative, Inserm CESP, Villejuif, France. [2]Université de Paris, Université Paris-Saclay, UVSQ, Inserm UMS11, Villejuif, France. [3]Inserm U011 Constances cohort, Villejuif, France.

Members of the 3C-Dijon Study: Christophe Tzourio[1], Stéphanie Debette[2], Carole Dufouil[1], Aïcha Soumaré[1], Morgane Lachaize[2], Nathalie Fievet[3], Amandine Flaig[3]

[1]University of Bordeaux; Bordeaux Population Health Center, INSERM U1219, Bordeaux, France. [2]University of Bordeaux; Bordeaux Population Health Center, INSERM U1219; Bordeaux University Hospital, Department of Neurology, Institute of Neurodegenerative Diseases, Bordeaux, France. [3]Laboratoire d'Analyses Génomiques - Centre de Ressources Biologiques; Institut Pasteur de Lille, Lille, France.

Member of the Cerba HealthCare: Fernando Martin[1]

[1]Cerba HealthCare, Issy-les-Moulineaux, France.

Members of the Etablissement Français du Sang study group: Brigitte Bonneaudeau[1], Fabrice Cognasse[5,6], Dorothée Cannet[2], Pierre Gallian[3], Michel Jeanne[4], Pascal Morel[1], Magali Perroquin[4], Pascale Richard[1], Pierre Tiberghien[1], Hind Hamzeh-Cognasse[5,6]

[1]Etablissement Français du Sang, La Plaine St-Denis, France. [2]Etablissement Français du Sang, Dijon, France. [3]Etablissement Français du Sang, Marseille, France. [4]Etablissement Français du Sang, Bordeaux, France. [5]Etablissement Français du Sang, Saint-Étienne, France. [6]SAINBIOSE, INSERM, U1059, University of Lyon, Université Jean-Monnet Saint-Étienne.
